# Modelling time‐course relationships with multiple treatments: Model‐based network meta‐analysis for continuous summary outcomes

**DOI:** 10.1002/jrsm.1351

**Published:** 2019-05-29

**Authors:** Hugo Pedder, Sofia Dias, Margherita Bennetts, Martin Boucher, Nicky J. Welton

**Affiliations:** ^1^ Department of Population Health Sciences, Bristol Medical School University of Bristol Bristol UK; ^2^ Pharmacometrics Pfizer Ltd Kent UK

## Abstract

**Background:**

Model‐based meta‐analysis (MBMA) is increasingly used to inform drug‐development decisions by synthesising results from multiple studies to estimate treatment, dose‐response, and time‐course characteristics. Network meta‐analysis (NMA) is used in Health Technology Appraisals for simultaneously comparing effects of multiple treatments, to inform reimbursement decisions. Recently, a framework for dose‐response model‐based network meta‐analysis (MBNMA) has been proposed that combines, often nonlinear, MBMA modelling with the statistically robust properties of NMA. Here, we aim to extend this framework to time‐course models.

**Methods:**

We propose a Bayesian time‐course MBNMA modelling framework for continuous summary outcomes that allows for nonlinear modelling of multiparameter time‐course functions, accounts for residual correlation between observations, preserves randomisation by modelling relative effects, and allows for testing of inconsistency between direct and indirect evidence on the time‐course parameters. We demonstrate our modelling framework using an illustrative dataset of 23 trials investigating treatments for pain in osteoarthritis.

**Results:**

Of the time‐course functions that we explored, the E_max_ model gave the best fit to the data and has biological plausibility. Some simplifying assumptions were needed to identify the ET_50_, due to few observations at early follow‐up times. Treatment estimates were robust to the inclusion of correlations in the likelihood.

**Conclusions:**

Time‐course MBNMA provides a statistically robust framework for synthesising evidence on multiple treatments at multiple time points. The use of placebo‐controlled studies in drug‐development means there is limited potential for inconsistency. The methods can inform drug‐development decisions and provide the rigour needed in the reimbursement decision‐making process.

## 1 INTRODUCTION

In drug development, a key decision is whether to proceed to a further clinical trial and if so, which follow‐up outcomes to measure and which comparator agent(s) to include. To inform this decision, it is important to understand the onset and duration of action of not just the agent under development, but also competitor agents. In addition, understanding the time‐course of pharmaceutical agents is useful for licencing agencies when making decisions on safety and efficacy of a new agent, and for reimbursement agencies making policy decisions based on manufacturer submissions where different studies and agents have reported results at different follow‐up times.

Model‐based meta‐analysis (MBMA) is a technique for synthesising results of multiple studies, usually randomised controlled trials (RCTs), to gain understanding of the pharmacodynamic profile of different agents during drug development.^1^ Key characteristics within this profile are the dose‐response and time‐course relationships of an agent, and how they compare with competitors. MBMA has been used to understand these relationships across studies.^2^, ^3^, ^4^ Some MBMAs have used indirect evidence to compare several agents simultaneously.^5^, ^6^, ^7^ However, these types of analyses typically model each agent separately, based on pooling individual study arms. Whilst this approach allows the inclusion of nonrandomised and/or single‐arm studies and has been proposed in the meta‐analysis literature,^8^ it breaks the randomisation within RCTs and ignores within‐study comparisons, effectively losing the advantages of the RCT design and potentially inducing bias in the resulting estimates.^9^

Network meta‐analysis (NMA) allows evidence on multiple treatments to be combined provided they form a connected network of treatment comparisons (where treatment refers to a distinct formulation, such as agent and dose combination).^10^, ^11^, ^12^, ^13^, ^14^ NMA pools evidence from RCTs in a way that respects the randomisation in the design of the included studies. It strengthens inference by combining all evidence (both direct and indirect) on the comparisons of interest, under the assumption of consistency—agreement between direct and indirect evidence on the same treatment comparison. When conducting an NMA, it is essential to test for inconsistency where possible and consider results accordingly, as the validity of the resulting estimates rests upon the consistency assumption. Methods have been developed that formally test for inconsistency in NMA,^15^, ^16^ something which has not previously been possible when making indirect comparisons in MBMA.

However, recently, MBMA has been extended to incorporate a formal consistency framework by combining ideas from NMA and MBMA in the context of dose‐response models with multiple agents.^17^ This model‐based network meta‐analysis (MBNMA) approach respects the randomisation in the included RCTs and allows for formal testing of inconsistency in the network of evidence.

Mawdsley et al^17^ described their method for dose‐response models with an outcome at a single time‐point. In phase II and III trials, there are often multiple follow‐up times reported within a study, which represent repeated measures on the same individuals and so will be correlated. This correlation needs to be accounted for either by modelling the time‐course,^18^, ^19^, ^20^ or with a multivariate likelihood,^21^, ^22^, ^23^, ^24^ or both.^2^, ^25^ Typically for meta‐analysis, only aggregated data are available in published RCTs. This can lead to additional complexities when accounting for correlations between repeated measurements, as the correlation structure may not be known.

Whilst repeated observations over time may be considered discrete observations, from the perspective of drug development, where the focus is on predicting relative efficacy at different time points, it is desirable to estimate a continuous function that describes the relationship between relative effects over time. In this paper, we describe the MBNMA approach with time‐course models for repeated observations within the included studies. We also present methods to assess whether the included evidence exhibits inconsistency. Note that in this paper we focus on the time‐course relationship only, and so the estimates are obtained for each distinct treatment regimen (agent and dose combination).

The paper is organised as follows. We begin by describing the MBNMA framework to incorporate multiple follow‐up times for continuous summary outcomes using models for time‐course. We present a general framework, indicating some of the common functional relationships typically seen in practice and accounting for residual correlation between time points. We also outline an approach for model selection and critique and present methods to assess consistency in the time‐course setting. We illustrate the methods with an example dataset of treatments for pain in osteoarthritis. Finally, we end with a discussion of the methods in the context of earlier work and indicate areas for further developments.

## 2 METHODS

### 2.1 Likelihood for continuous summary outcomes

Although individual patient data (IPD) may be available from a manufacturer's own study, it is likely that only aggregate level data from publications are available from other studies. We therefore develop our model at the level of study summaries (eg, mean differences). We assume that for each study we have a continuous summary outcome, such as mean outcome or log‐odds of response, *y*_*i*,*k*,*m*_, together with standard errors, *se*_*i*,*k*,*m*_, reported for each study *i*, arm *k* = 1, … ,*K*_*i*_, and at time point *m* = 1, … ,*M*_*i*_, where study *i* has *K*_*i*_ arms and reports at *M*_*i*_ time points and *s*_*i*,*m*_ gives the actual time corresponding to the *m*^*th*^ time point in study *i*. This formulation allows for different studies to report at different times. Typically, *m* = 1 represents a baseline observation at time *s*_*i*,1_ = 0. The treatment given in study *i*, arm *k*, is indicated by *t*_*i*,*k*_.

Because we have repeated measures from the same individuals within each study, the observations may be correlated, which can be captured with a multivariate normal likelihood:

(1) $$y_{i,k}\;\sim \;\mathrm{MVN}\left(\theta_{i,k},\Sigma_{i,k}\right)$$

where ***y***_*i*,*k*_ is a vector of the observed summary measures over time points, **θ**_*i*,*k*_ is a vector of modelled outcomes, and **Σ**_*i*,*k*_ is an *M*_*i*_ × *M*_*i*_ covariance matrix:

$$\Sigma_{i,k}=\left(\begin{array}{cccc} {\mathrm{se}}_{i,k,1}^{2} & \rho_{i,k,1,2}{\mathrm{se}}_{i,k,1}{\mathrm{se}}_{i,k,2} & \ldots & \rho_{i,k,1,M_{i}}{\mathrm{se}}_{i,k,1}{\mathrm{se}}_{i,k,M_{i}}\\ \rho_{i,k,1,2}{\mathrm{se}}_{i,k,1}{\mathrm{se}}_{i,k,2} & {\mathrm{se}}_{i,k,2}^{2} & \ldots & \rho_{i,k,2,M_{i}}{\mathrm{se}}_{i,k,2}{\mathrm{se}}_{i,k,M_{i}}\\ \vdots & \vdots & \ddots & \vdots \\ \rho_{i,k,1,M_{i}}{\mathrm{se}}_{i,k,1}{\mathrm{se}}_{i,k,M_{i}} & \ldots & \ldots & {\mathrm{se}}_{i,k,M_{i}}^{2} \end{array}\right)$$

where $\rho_{i,k,m_{1},m_{2}}$ is the within‐study correlation between summary measures at time points *m*_1_ and *m*_2_ for study *i* arm *k*. In practice, correlations are rarely reported in the literature and will only be available from studies where we have IPD. In addition, the correlations estimated from any available IPD will be at the individual level, which may be different to correlations at the summary level.^26^

One approach to deal with unknown within‐study correlations at the summary level, if IPD are available or information on this correlation can be obtained from external data, is to assume that the correlations seen between time points for individual patients are the same as those seen for summary measures, and also that correlations in the study for which correlation information is available also apply in the aggregate data trials.^27^, ^28^ However, this might lead to ecological bias as there is no guarantee that correlations at the individual level will be the same as at the aggregate level. An alternative is to estimate within‐study correlations based on the aggregate data summaries by giving prior distributions to the $\rho_{i,k,m_{1},m_{2}}$. This approach allows the possibility of using informative prior distributions based on information gained from external data. In order to identify correlation parameters, some constraints will be required, such as assuming a particular covariance structure. For example, a compound symmetry structure can be assumed, in which a single parameter, *ρ*, is estimated for the correlation between all time points (assumed to be the same across all studies): $\rho_{i,k,m_{1},m_{2}}=\rho$. Alternatively, it might be more reasonable to assume an autoregressive AR(1) structure in which covariances are dependent on the amount of time between observations where $\rho_{i,k,m_{1},m_{2}}=\rho^{\frac{s_{i,m_{2}}-s_{i,m_{1}}}{s_{i,2}-s_{i,1}}}$.

When studies that report mean change from baseline and final values at each time point are included, they can contribute information to $\rho_{i,k,m_{1},m_{2}}$ by modelling separate likelihoods for both pieces of data (see below).^29^

#### 2.1.1 Mean change from baseline by time

Where aggregated summaries are reported as mean change from baseline (baseline corresponding to m = 1), we have summaries for time points *m* = 2, … ,*M*_*i*_ defined as $y_{i,k,m}^{\text{change}}=\left(y_{i,k,m}-y_{i,k,1}\right)$ and their standard errors ${\mathrm{se}}_{i,k,m}^{\text{change}}$. Covariances between mean changes from baseline across time‐points *m*_1_ and *m*_2_ (dropping the *i*,*k* subscripts for ease of exposition) are

$$\mathrm{Cov}\left(\left(y_{m_{1}}-y_{1}\right),\left(y_{m_{2}}-y_{1}\right)\right)=\rho_{m_{1},m_{2}}{\mathrm{se}}_{m_{1}}{\mathrm{se}}_{m_{2}}-\rho_{1,m_{1}}{\mathrm{se}}_{1}{\mathrm{se}}_{m_{1}}-\rho_{1,m_{2}}{\mathrm{se}}_{1}{\mathrm{se}}_{m_{2}}+{\mathrm{se}}_{1}^{2}$$

which gives the (*m*_1_, *m*_2_)^*th*^ element of the covariance matrix for the mean change from baselines, $\Sigma_{i,k}^{\text{change}}$.

We can then give a multivariate normal likelihood to the aggregate mean outcomes for all time points:

$$y_{i,k}^{\text{change}}\;\sim \;\mathrm{MVN}\left(\theta_{i,k}^{\text{change}},\Sigma_{i,k}^{\text{change}}\right)$$

where

$$\theta_{i,k}^{\text{change}}=\left(\begin{array}{c} \theta_{i,k,2}-\theta_{i,k,1}\\ \theta_{i,k,3}-\theta_{i,k,1}\\ \vdots \\ \theta_{i,k,M_{i}}-\theta_{i,k,1} \end{array}\right).$$

By writing the model for mean change from baseline in terms of the model for mean outcomes, we can combine studies where some report mean outcomes and some report mean change from baseline by giving each type of data the appropriate likelihood and using a shared‐parameter model.^30^ If some studies report both mean outcomes and mean change from baseline, then both pieces of data can be included. Modelling both outcomes simultaneously provides sufficient evidence to estimate the correlations, $\rho_{i,k,m_{1},m_{2}}$.^29^

### 2.2 Time‐course model

We put the time‐course model on the aggregate‐level means:

$$\theta_{i,k,m}=f\left(s_{i,m},\lambda_{i,k}\right)$$

where *f* defines a functional relationship over time *s*, and **λ**_*i*,*k*_ = (*λ*_0,*i*_,*λ*_1,*i*,*k*_,*λ*_2,*i*,*k*_,…) are a set of parameters that describe the relationship in mean outcomes over time. In all time‐course models, there will be a “nuisance parameter” *λ*_0,*i*_ which represents the “intercept” at time, common across arms. Note that for many time‐course models the *λ*_0,*i*_ parameters will cancel out when using change from baseline data. We put our modelling assumptions on the remaining parameters, *λ*_1,*i*,*k*_,*λ*_2,*i*,*k*_,…, leaving the *λ*_0,*i*_ unconstrained (achieved in a Bayesian analysis by giving independent vague prior distributions to the *λ*_0,*i*_ parameters).

#### 2.2.1 Exponential model

One of the most commonly used models is the exponential model, which has intercept *λ*_0,*i*_, and a single parameter of interest, *λ*_1,*i*,*k*_, which represents the rate at which the mean outcome falls over time, assuming a constant rate of growth/decay:

(2) $$\theta_{i,k,m}=\lambda_{0,i}\exp\left(\lambda_{1,i,k}s_{i,k,m}\right).$$

#### 2.2.2 Linear model

Another model with a single parameter of interest is the linear model:

(3) $$\theta_{i,k,m}=\lambda_{0,i}+\lambda_{1,i,k}s_{i,k,m}$$

where *λ*_0,*i*_ is the intercept and *λ*_1,*i*,*k*_ the fall in mean outcome for a unit increase in time.

#### 2.2.3 E_max_ model

A functional form commonly used in pharmacometrics, which has two parameters of interest, is the E_max_ model:

(4) $$\theta_{i,k,m}=\lambda_{0,i}+\frac{\lambda_{1,i,k}\times s_{i,m}}{\lambda_{2,i,k}+s_{i,m}}$$

where the intercept *λ*_0,*i*_, often referred to as E_0_, is the initial outcome at baseline (time = 0), *λ*_1,*i*,*k*_, typically referred to as E_max_, is the maximum possible effect of a treatment relative to baseline, and *λ*_2,*i*,*k*_, typically referred to as ET_50_, is the time point at which 50% of the maximum treatment effect has been achieved.

#### 2.2.4 Piecewise linear model

Piecewise models can allow for considerable flexibility, though they may not so accurately resemble true biological relationships and may not be appropriate when the intention is to predict values close to where the pieces meet (the “knots”). The simplest example of this is a two‐piece linear model with a single knot at *s* = *S*:

(5) $$\theta_{i,k,m}=\left\{\begin{array}{cc} \lambda_{0,i}+\lambda_{1,i,k}s_{i,m} & 0\leq s\leq S\\ \left(\lambda_{0,i}+\lambda_{1,i,k}S\right)+\lambda_{2,i,k}\left(s_{i,m}-S\right) & s>S \end{array}\right.$$

where *λ*_0,*i*_ is the intercept, *λ*_1,*i*,*k*_ the change in mean outcome for a unit change in time during time period (0,*S*), and *λ*_2,*i*,*k*_ the change in mean outcome for a unit change in time during time period after *S*. The intercept for the second piece (*λ*_0,*i*_+*λ*_1,*i*,*k*_*S*) ensures that the two regression lines meet at the knot.

### 2.3 Network meta‐analysis model

The NMA model describes the impact of treatments on one or more of the parameters of the time‐course model, *λ*_1,*i*,*k*_,*λ*_2,*i*,*k*_,…. If the NMA model is given for a single time‐model parameter, *λ*_1,*i*,*k*_, we have

$$g\left(\lambda_{1,i,k}\right)=\mu_{i}+\delta_{i,k}$$

for a given link function *g* which transforms the outcome to a scale where relative treatment effects may be expected to be additive. *μ*_*i*_ is the time‐course model parameter (on the transformed scale) for arm 1 of study *i*, and *δ*_*i*,*k*_ the study‐specific relative effect for the treatment used in arm *k* relative to arm 1 of study *i*.

For example, for an exponential time‐course model (Equation 2), it would be natural to put the NMA model on the log‐scale:

$$\log\left(\lambda_{1,i,k}\right)=\mu_{i}+\delta_{i,k}$$

where *μ*_*i*_ is the log growth/decay rate on arm 1, and *δ*_*i*,*k*_ is the log rate‐ratio for treatment arm *k* compared with treatment arm 1, of study *i*.

The *μ*_*i*_ are nuisance parameters and given independent vague prior distributions in a Bayesian analysis to allow these to be unconstrained. By treating these as nuisance parameters, the focus of this modelling strategy is on estimating relative treatment effects rather than on characterising the time‐course on the reference treatment (eg, placebo effect). In fact, because different studies may have included different control (arm 1) treatments, the *μ*_*i*_ do not have a consistent interpretation across studies.

Treatment effects can be either assumed common (“fixed”) or similar/exchangeable (“random”) across studies. For the random effects model, study‐specific treatment effects are assumed to be normally distributed around a mean treatment effect that adheres to the consistency relationships, with common between‐studies variance *τ*^2^ across treatment comparison:

(6) $$\delta_{i,k}\;\sim \;N\left(d_{1,t_{i,k}}-d_{1,t_{i,1}},\tau^{2}\right).$$

The consistency relationships reflect the comparison made between the treatment *t*_*i*,*k*_ used on arm *k* and the treatment *t*_*i*,1_ used on arm 1 of each study. The fixed effect model is obtained by setting *τ*^2^ = 0. The model estimates “basic parameters” *d*_1,*k*_, the pooled mean relative effect for treatment *k* relative to treatment 1 (the reference treatment for the NMA). The *d*_1,*k*_ are each given independent vague normal priors in a Bayesian analysis. All other relative effects for treatment *k* relative to treatment *c*, *d*_*c*,*k*_, can then be derived from the consistency relationships^12^, ^30^:

(7) $$d_{c,k}=d_{1,k}-d_{1,c}.$$

Time‐course functions with multiple (nonintercept) time‐course parameters may have NMA models specified for one or more of these parameters, although a relatively rich dataset is required to estimate NMA models with more than one treatment effect parameter.

Suppose we expect the treatments to influence two parameters of the time‐course model, *λ*_1,*i*,*k*_ and *λ*_2,*i*,*k*_ (for example these could represent E_max_ and ET_50_). The NMA model proceeds as for a single parameter; however, for a random effects model, we need to allow for correlations between the study‐specific treatment effects on the two time‐course parameters. Note that the link functions *g* do not have to be the same for the different parameters:

(8) $$\begin{array}{l} g_{1}\left(\lambda_{1,i,k}\right)=\mu_{1,i}+\delta_{1,i,k}\\ g_{2}\left(\lambda_{2,i,k}\right)=\mu_{2,i}+\delta_{2,i,k}\\ \mathrm{etc}. \end{array}$$

The random effects model for *δ*_1,*i*,*k*_, *δ*_2,*i*,*k*_, etc needs to be multivariate to allow for correlations between relative effects on the different time‐course parameters.

For example, for the E_max_ model, the E_max_ parameter *λ*_1,*i*,*k*_ can be positive or negative, and so we can put the model on the natural scale, whereas the ET_50_ parameter *λ*_2,*i*,*k*_ may only take positive values, and so it makes sense to model this on the log scale, giving

$$\lambda_{1,i,k}=\mu_{1,i}+\delta_{1,i,k}\text{log}\left(\lambda_{2,i,k}\right)=\mu_{2,i}+\delta_{2,i,k}$$

with a bivariate random effects distribution:

(9) $$\left(\begin{array}{c} \delta_{1,i,k}\\ \delta_{2,i,k} \end{array}\right)\;\sim \;N\left(\left(\begin{array}{c} d_{1,1,t_{i,k}}-d_{1,1,t_{i,1}}\\ d_{2,1,t_{i,k}}-d_{2,1,t_{i,1}} \end{array}\right),\left(\begin{array}{cc} \tau_{1}^{2} & \rho_{\delta }\tau_{1}\tau_{2}\\ \rho_{\delta }\tau_{1}\tau_{2} & \tau_{2}^{2} \end{array}\right)\right)$$

where all parameters are as before, with an extra subscript to indicate whether they relate to *λ*_1,*i*,*k*_ or *λ*_2,*i*,*k*_. The correlation between the treatment effects on the two parameters is given by *ρ*_*δ*_. Different parameterisations are available for the between‐studies covariance matrix that may be more computationally stable, such as a Cholesky parameterization or a spherical parameterization.^31^ Fixed effect models can be obtained by setting the between‐study variance parameters to 0.

### 2.4 Multi‐arm trials

When including multi‐arm trials, it is important to account for correlation between relative effects within a trial, as all relative effects will have the same comparator. For a common between‐study variance, the correlation between these relative effects will be 0.5.^11^ For MBNMAs with a single nonintercept time‐course parameter, this can be done either using a multivariate normal distribution to model a vector of random effects,^30^ or, for the purposes of writing more generic code, using a conditional univariate distributions formulation for the random effect of arm *k* > 2, given all arms from 2 to *k* − 1 32:

$$\left.\delta_{i,k}\right|\left(\begin{array}{c} \delta_{i,2}\\ \vdots \\ \delta_{i,\left(k-1\right)} \end{array}\right)\;\sim \;N\left(\left(d_{1,t_{i,k}}-d_{1,t_{i,1}}\right)+\frac{1}{k-1}\sum_{j=1}^{k-1}\left[\delta_{i,j}-\left(d_{1,t_{i,j}}-d_{1,t_{i,1}}\right)\right],\frac{k}{2\left(k-1\right)}\sigma^{2}\right).$$

When modelling multiple nonintercept time‐course parameters, the correlation between relative effects can be modelled simultaneously to the correlation between the parameters *ρ*_*δ*_ using a multivariate normal distribution on a vector of random effects, ***δ***_*i*_, whose length is equal to the number of parameters multiplied by the number of arms, *K*_*i*_, in study *i*. For models with two parameters of interest, this is

$$\delta_{i}=\left(\begin{array}{c} \delta_{1,i,k}\\ \vdots \\ \delta_{1,i,K}\\ \delta_{2,i,k}\\ \vdots \\ \delta_{2,i,K} \end{array}\right)\;\sim \;N\left(\left(\begin{array}{c} d_{1,1,t_{i,k}}-d_{1,1,t_{i,1}}\\ \vdots \\ d_{1,1,t_{i,K}}-d_{1,1,t_{i,1}}\\ d_{2,1,t_{i,k}}-d_{2,1,t_{i,1}}\\ \vdots \\ d_{2,1,t_{i,K}}-d_{2,1,t_{i,1}} \end{array}\right),\Omega_{i}\right).$$

The covariance matrix, Ω_*i*_, is a 2*K*_*i*_ × 2*K*_*i*_ matrix:

$$\Omega_{i}=\left(\begin{array}{ccccc} \tau_{1}^{2} & \frac{\tau_{1}^{2}}{2} & \cdots & 2\rho_{\delta }\tau_{1}\tau_{2} & \rho_{\delta }\tau_{1}\tau_{2}\\ \frac{\tau_{1}^{2}}{2} & \tau_{1}^{2} & \cdots & \rho_{\delta }\tau_{1}\tau_{2} & 2\rho_{\delta }\tau_{1}\tau_{2}\\ \vdots & \vdots & \ddots & \vdots & \cdots \\ 2\rho_{\delta }\tau_{1}\tau_{2} & \rho_{\delta }\tau_{1}\tau_{2} & \cdots & \tau_{2}^{2} & \frac{\tau_{2}^{2}}{2}\\ \rho_{\delta }\tau_{1}\tau_{2} & 2\rho_{\delta }\tau_{1}\tau_{2} & \cdots & \frac{\tau_{2}^{2}}{2} & \tau_{2}^{2} \end{array}\right).$$

### 2.5 Simplifying modelling assumptions

For models with many parameters, there may be insufficient data to be able to estimate all parameters (ie, the parameters may not be identifiable). To aid identifiability when there are two or more non‐nuisance parameters, simplifying assumptions can be made to constrain the parameters. In this way, a model can be constrained to be as complex or simple as the data allow, provided there is biological plausibility for any simplification. Note that any shared parameters will be more influenced by studies/treatments with more information (ie, those with more observations within studies). One or more of the following may be considered:

#### 2.5.1 Fixed effect models

One or more of the modelled parameters could be modelled as a fixed treatment effect, reflecting an assumption of homogeneity where different studies of the same comparison estimate a common effect. So, if there is a fixed effect model on the second parameter, Equation 9 becomes

$$\begin{array}{l} \delta_{1,i,k}\;\sim \;N\left(d_{1,1,t_{i,k}}-d_{1,1,t_{i,1}},\tau_{1}^{2}\right)\\ \delta_{2,i,k}=d_{2,1,t_{i,k}}-d_{2,1,t_{i,1}} \end{array}$$

and if there is a fixed effect model on both parameters, Equation 9 becomes

$$\begin{array}{l} \delta_{1,i,k}=d_{1,1,t_{i,k}}-d_{1,1,t_{i,1}}\\ \delta_{2,i,k}=d_{2,1,t_{i,k}}-d_{2,1,t_{i,1}}. \end{array}$$

#### 2.5.2 Class‐effect models

Relative treatment effects for one (or more) of the parameters could be assumed to come from a hierarchical model with a common mean, which may depend on class^13^, ^33^, ^34^, ^35^:

(10) $$d_{2,1,k}\;\sim \;\left(D_{2,\text{class}},\tau_{\text{class}}^{2}\right)\;\text{for}\;k\in \left\{\text{class}\right\}.$$

For example, in the E_max_ model, it may be that it is reasonable to assume that treatments within the same class might have a similar onset of action (and so have similar, exchangeable treatment effects on ET_50_) but reach different maximum effects (E_max_).

An even more constrained model fixes the treatment effects within a class to be equal:

(11) $$d_{2,1,k}=D_{2,\text{class}}\;\text{for}\;k\in \left\{\text{class}\right\}.$$

#### 2.5.3 Constrain the baseline effect

To further aid identifiability of treatment effects, it may be necessary to impose further constrains on the baseline for one (or more) of the time‐course parameters, for example with an exchangeable model:

(12) $$\mu_{2,i}\;\sim \;N\left(\eta_{2},\sigma_{\mu_{2}}^{2}\right).$$

This would imply that the reference treatment effects for each study were assumed to be distributed about a single common mean effect, *η*_2_, and would therefore only be suitable in networks for which all included trials have the same reference treatment (eg, placebo).

#### 2.5.4 Reduce to a single treatment effect

For models with multiple (nonintercept) time‐course parameters, a further simplification is to only model treatment effects on one of the time‐course parameters. The other parameters are assumed to be treatment independent and modelled on an absolute, rather than relative, scale:

$$g_{2}\left(\lambda_{2,i,k}\right)=\mu_{2,i,k}.$$

The *μ*_2,*i*,*k*_ could be left unconstrained or assumed exchangeable for each treatment within a class.

### 2.6 Testing for inconsistency

To test whether the consistency assumption (Equation 7) holds, several approaches have been proposed for identifying inconsistency between direct and indirect evidence that arises within a closed “loop” of treatments for which independent sources of information are available.^15^, ^36^ It is important to note that available loops of treatments to test for inconsistency will depend on the choice of reference treatment used in the network.^36^, ^37^ In addition, the evidence provided by studies with three or more arms is not independent (due to the common reference arm), and within‐study relative effects must be internally consistent. Therefore, loops of evidence consisting only of studies with three or more arms will always be consistent.

Furthermore, the choice of reference treatment for a study with three or more arms can affect whether it is possible to test for inconsistency. For example, suppose we have three different studies providing evidence on P (Placebo) vs N (Naproxen 1000 mg/d), P vs C (Celebrex 200 mg/d), and PvsNvsC, respectively. If we take P as the reference for the three‐arm trial, then the three‐arm trial provides estimates of PvsN and PvsC, so the model only estimates PvsN and PvsC directly (see Figure S3A ‐ Supporting Information). The NvsC effect is derived from the PvsN and PvsC estimates. If, on the other hand, we take N as the reference for the three‐arm trial, then the three‐arm trial provides estimates of PvsN and NvsC, which together with the two‐arm evidence provides independent estimates of PvsN, PvsC, and NvsC, and we can test for inconsistency. We use the convention that we take placebo as reference for all studies that include a placebo arm. In studies without placebo, we use the first drug alphabetically, at its lowest dose.

An unrelated mean effects (UME) model does not include constraints forced by the consistency equations and is equivalent to fitting separate pairwise meta‐analyses to each direct comparison whilst sharing treatment‐independent parameters across all the comparisons,^16^ such as between‐study heterogeneity or treatment‐independent time‐course parameters. The results from this model can then be compared with those from the MBNMA. A better model fit (lower deviance) or lower standard deviations (SD) for exchangeable parameters or random treatment effects would suggest that inconsistency may be present in the network.

A more explicit method for testing inconsistency for specific comparisons is the node‐splitting method.^15^ This technique involves splitting the evidence for a given comparison within a loop of treatments into “direct” evidence from head‐to‐head RCT comparisons and “indirect” evidence that arises from the consistency relationships. A Bayesian *P*‐value can be calculated for the treatment effects estimated using the direct and indirect evidence, which represents the proportion of the two posterior distributions that overlap.

Note that when performing pairwise meta‐analyses to estimate direct evidence, the sharing of parameters across direct comparisons for which limited information is available can make tests for inconsistency conservative, and this should be borne in mind when interpreting them.

For models with multiple time‐course parameters (Equation 8), it is important to consider that inconsistency may be present for treatment effects on either *or both* of the time‐course parameters.

Donegan et al^38^ present inconsistency models to explore consistency on two parameters. We suggest testing for inconsistency on each time‐course parameter separately, because if inconsistency is identified in either parameter for a given comparison, then this should be a cause for concern and should prompt further investigation of the included studies to identify the potential cause.

Multiple testing may also be an issue here, as the number of tests in a typical network will be multiplied by the number of time‐course parameters in the MBNMA model. However, inconsistency tests are typically underpowered, and we advise erring on the side of caution as it is better to incorrectly identify inconsistency when there is none present than to incorrectly fail to identify inconsistency when true inconsistency is present.

### 2.7 Treatment ranking for time‐course relationships

In NMA, it is common to calculate ranking probabilities (probability of being first best, second best, etc) for each treatment within a network, as this is an easily interpretable measure for decision‐makers to use. In time‐course MBNMA, we can rank on any function of the time‐course model, which can allow for an extremely flexible decision‐making framework. This could include ranking based on any one of multiple time‐course parameters or ranking on the predicted response at a desired follow‐up time. For time‐course functions with multiple parameters, note that the ranking of treatment effects may differ for different time‐course parameters. For example, we could have a treatment that ranks highest for ET_50_ indicating that it acts more quickly than other treatments but ranks lowest for E_max_ indicating that the overall response is lower than for other treatments.

For models with less easily interpretable time‐course parameters, it may also be beneficial to have an overall ranking that takes into account the full time‐course relationship. Calculating the Area Under the Curve (AUC) for the time‐course relationship for each treatment using parameters estimated from the model is a pragmatic way of doing this. However, care must be taken when choosing the duration of time‐course over which to calculate AUC, as treatment rankings may be sensitive to this choice.

### 2.8 Measures of model fit

Models are implemented using a Bayesian approach, and therefore we use the posterior mean of the deviance to compare the goodness‐of‐fit of the models,^39^ where smaller values of deviance are preferred. Model selection is based on the Deviance Information Criterion (DIC) which represents a compromise between model fit and model complexity,^39^, ^40^ defined as the sum of the posterior mean deviance (a measure of fit) and the effective number of parameters (a measure of complexity). We use pD calculated using the Kullback‐Leibler information divergence as the effective number of parameters.^41^ For the selected final model (based on a univariate likelihood), we also report the posterior mean of the residual deviance (defined as the deviance for the model minus the deviance for a saturated model), which can be compared with the number of unconstrained data points to give an overall measure of model fit. Lack of fit is explored by plotting an appropriate posterior summary (median if skewed) of the contribution to the residual deviance for each data‐point against time. Note, we do not compute residual deviances for models with a multivariate likelihood in which the correlation between time points is estimated from the data, as the saturated model is not uniquely defined.

### 2.9 Model selection strategy

We propose a step‐by‐step approach for model selection of time‐course relationships in MBNMA, recognising that the available evidence may not be sufficient to be able to estimate some of the more complex, but less restrictive, models.

1. Plot study summaries (mean outcome) against time to visually identify potential time‐course function candidates and obtain expert opinion to assess their biological plausibility if necessary.
2. Fit candidate time‐course models with fixed treatment effects using a univariate likelihood that does not account for correlations over time (univariate models). Use simplifying modelling assumptions, described above, if necessary to estimate the models given the available data.
  For each of these fitted models, plot the posterior median of the contribution of each data‐point to the residual deviance against time to check fit and to identify alternative time‐course relationships to explore. Compare model fit statistics (posterior mean deviance and DIC) and select a time‐course model with adequate balance between fit and complexity (lowest DIC) that also has biological plausibility.
3. For the selected univariate time‐course model, fit random treatment effects models (if possible) with the available data. Use model fit statistics and inspection of between studies SD parameters to assess presence of heterogeneity and choose between fixed and random treatment effect models.
4. Check fit of the selected univariate model by comparing posterior mean residual deviance to the total number of data points
5. For the selected time‐course and treatment effects model, fit models with multivariate likelihoods that account for correlations over time with different covariance structures. Select between the univariate and multivariate formulations based on estimated correlations and robustness of treatment effects obtained (preferring the simpler models with lower pV). Note the deviance statistics are not directly comparable for models with different likelihoods and so cannot be used for model selection.
6. Check for consistency in final selected model (where possible)
  - Run UME model
  - If suggestive of possible inconsistency, perform node splitting of closed loops

### 2.10 Illustrative example—Pain in osteoarthritis

The methodology is illustrated using a dataset of RCTs investigating treatments for pain in patients with osteoarthritis. Pain was measured on the Western Ontario and McMaster Universities Arthritis Index (WOMAC) scale^42^ and was recorded at multiple time points up to a maximum of 24 weeks. In order to maintain a consistent imputation method for missing data across studies, only those with last observation carried forward analyses (LOCF) were included, as this was the imputation method reported in the majority of papers. Agents with multiple doses were split to form the network of treatments, meaning that each combination of agent and dose was considered to be a separate treatment. Although SDs were typically available at baseline, they were missing for 269 out of 345 observations and were therefore imputed accounting for changes over time using the method of Boucher.^43^ We acknowledge that this is a high proportion of data points for which to impute SD, but our aim here is to illustrate the method rather than to provide clinically useful treatment estimates. We note however that in pharmacometrics, SD is not always reported as weighting is often performed using sample size—in practice, we would always recommend that SDs be reported and measured.

**Figure 1:**
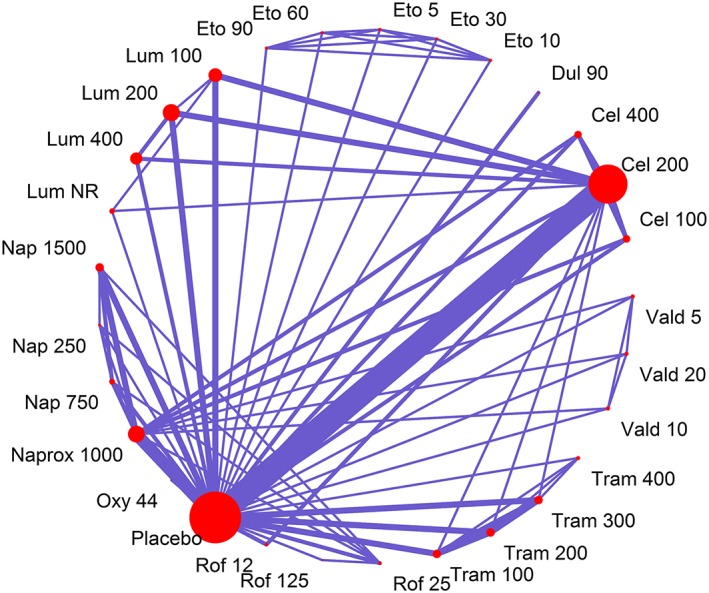
Network of treatment comparisons within the MBNMA for the illustrative dataset of 24 RCTs for pain in osteoarthritis. Each treatment is represented by a node. Where direct RCT evidence exists for a particular comparison, the nodes are connected by a line, the thickness of which is proportional to the number of comparisons. All numbers represent doses (total daily dose in mg). Abbreviations: Cel = Celebrex, Dul = Duloxetine, Eto = Etoricoxib, Lum = Lumiracoxib, Naprox = Naproxcinod, Nap = Naproxen, Oxy = Oxycodone, Rof = Rofecoxib, Tram = Tramadol, Vald = Valdecoxib, NR = Dose not reported [Colour figure can be viewed at wileyonlinelibrary.com]

**Figure 2:**
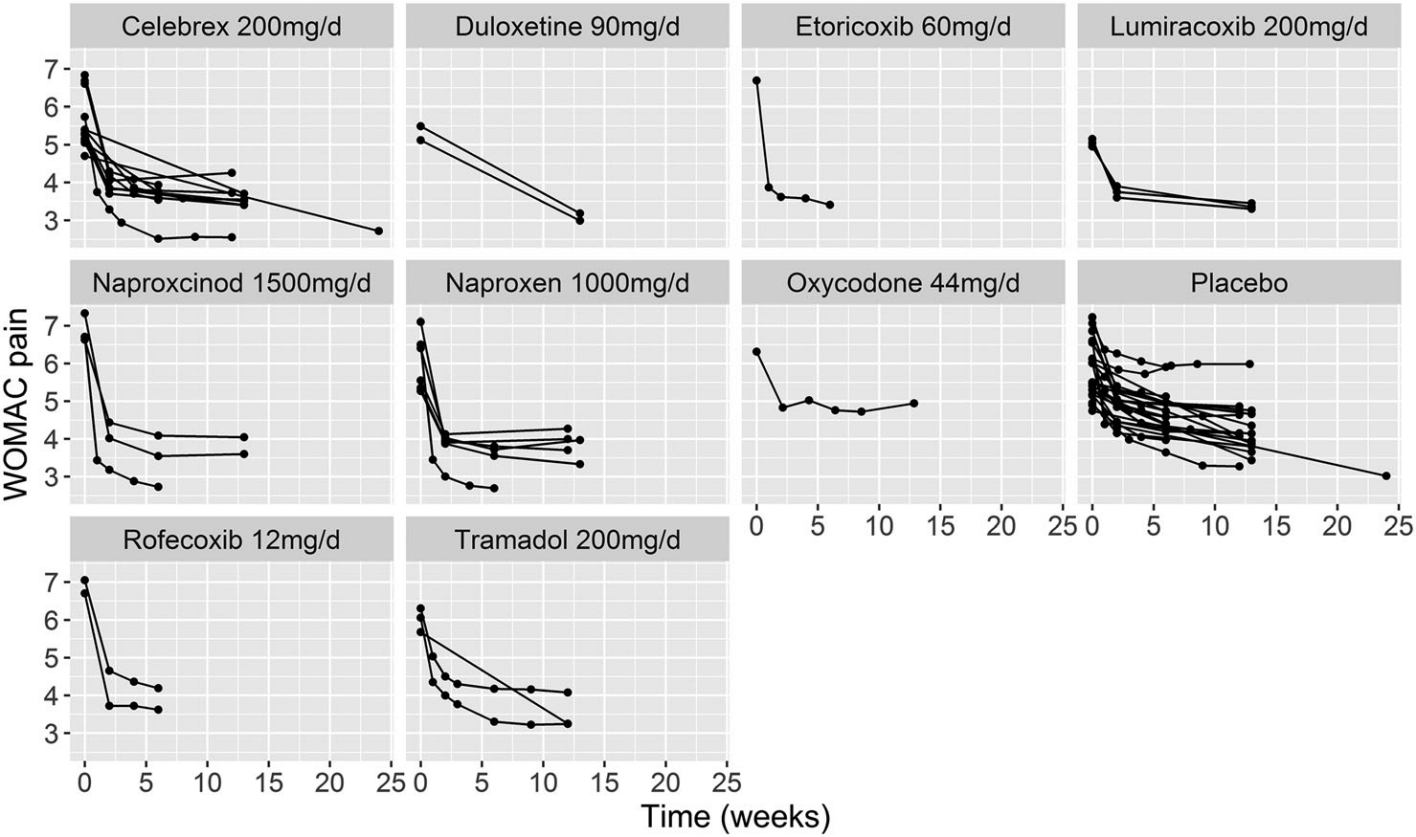
Plots of the mean WOMAC pain score for each of the studies in the pain in osteoarthritis dataset showing the most commonly reported dose for each agent, plotted over time

The illustrative dataset consists of 23 RCTs comparing 29 treatments. Each study has a median of 3.5 (range: 2‐7) follow‐up measurements, and all studies use LOCF imputation for analyses. Figure 1 shows the network of comparisons in the data, and Figure 2 shows mean WOMAC pain in each study arm plotted over time for each treatment. The dataset is freely available in the Supporting Information (OsteoarthritisData.csv).^44^

### 2.11 Implementation

Models were estimated using JAGS 4.2.0 (codes in the Supporting Information). All fixed treatment effect models were run on three independent chains for 20 000 iterations following 80 000 burn‐in iterations with a thinning parameter of 10. For random treatment effect models, convergence took more iterations—models were run for 500 000 iterations following 400 000 burn‐in iterations with a thinning parameter of 15. Gelman's r statistic^45^ and visual inspection of the chains were used to assess convergence. Vague normal prior distributions (*N*(0,1000)) were given to the basic parameters *d*_1,*k*_, nuisance parameters *μ*_*i*_, and treatment effect‐independent time‐course parameters. For ET_50_ parameters in E_max_ models, it was necessary to ensure that they only took positive values so priors for these were specified on the log‐scale. The between‐study SD and SDs on exchangeable parameters were given uniform prior distributions (*U*(0,5)). We inspect the posterior for the SDs to ensure they are not being constrained by the prior. We also assessed sensitivity of results to the prior by fitting the same models using half‐normal prior distributions (*N*(0,6.25)). In models with a multivariate likelihood, *ρ* was given a uniform prior distribution (*U*(0,1)) to reflect the belief that outcomes at different time points in the same study are likely to be positively correlated. For bivariate models with two nonintercept parameters, the correlation between these parameters, *ρ*_*δ*_, was given a uniform prior distribution (*U*(−1,1)). For piecewise time‐course models, knot location was selected through trial‐and‐error by fitting models with different knot values (allowing a minimum of 0.1 weeks between knots in different models) and selecting the value from the best fitting (lowest mean posterior deviance) model.

## 3 RESULTS

Step 1. **Visually inspect the data plotted against time and consider biological plausibility of time‐course functions**

For all treatments, including placebo, visual inspection of the data shows that WOMAC scores decrease over time in a nonlinear fashion, with a rapid decline in pain during the first 1 to 2 weeks that quickly levels out (Figure 2). This suggests that a simple linear model will not be a good fit for the data, but that exponential, piecewise linear, or E_max_ models may be more suitable. Both exponential and E_max_ models have good biological plausibility and are frequently used for modelling pharmacodynamic properties of drugs. However, the limited number of observations at earlier follow‐up times suggests that there may not be enough information to identify parameters that model the rapid decline, such as ET_50_ parameters or parameters in the first piece of piecewise models.

For two treatments (Duloxetine 90 mg/d and Lumiracoxib (NR—dose not reported)), there is only data from studies with baseline measurement and a single follow‐up time compared with placebo, which is insufficient information to be able to identify any nonlinear candidate time‐course function. We assumed that these treatments followed the same time‐course function (though with different time‐course parameter values) as the other treatments in the network, and we consider the impact of this assumption in the discussion.

Step 2. **Compare time‐course models with univariate likelihood and fixed treatment effects**

**Figure 3:**
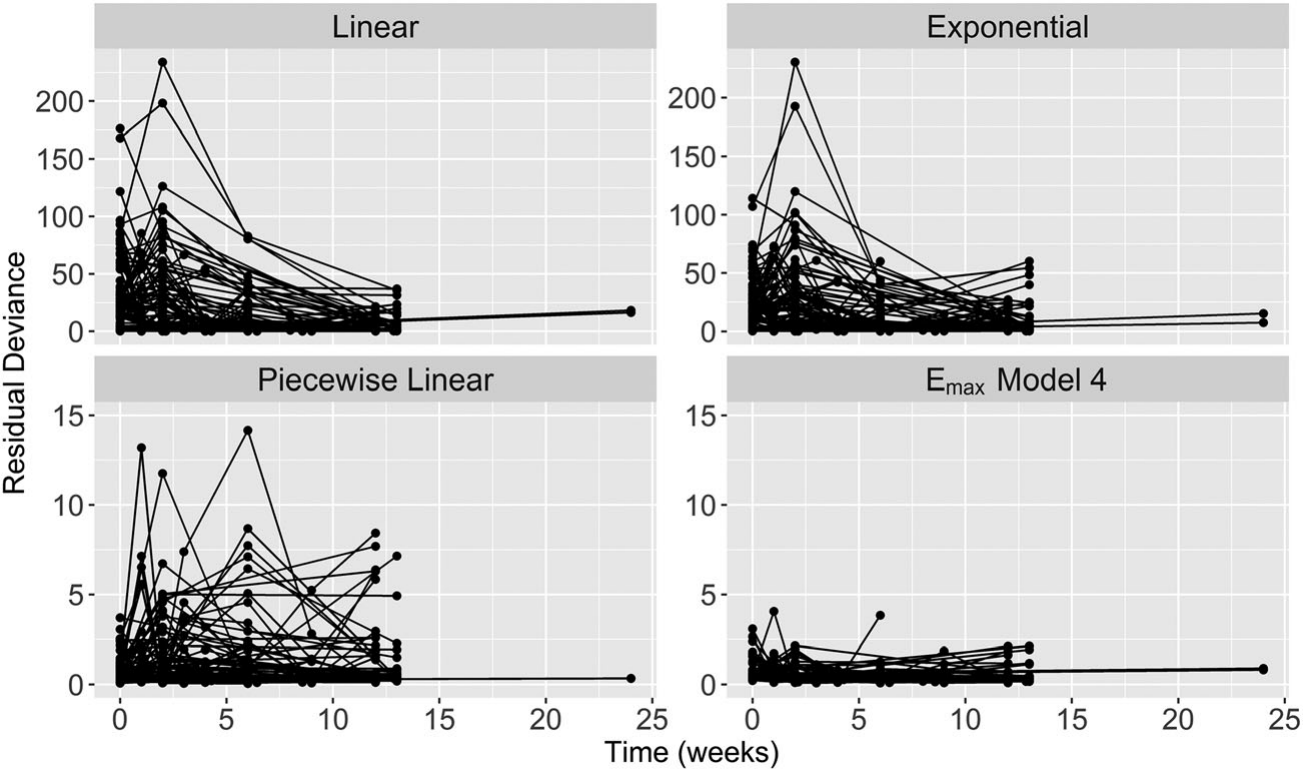
Median posterior residual deviance contributions over time from univariate fixed treatment effects models with linear, exponential, piecewise linear, and E_max_ (model 4) time‐course relationships in the pain in osteoarthritis dataset. Residual deviances closer to 0 indicate a better fitting model. Nonlinearity in these plots indicates that the effect of time has not been properly accounted for. The scales for residual deviance vary between the upper and lower panels

Table 1 shows model fit statistics for linear, exponential, piecewise linear, and E_max_ models (with various simplifying assumptions). As expected, a linear time‐course model gave a very poor fit to the data (Equation 3, Figure 3, posterior mean deviance = 6935.2—Table 1). An exponential time‐course was also a poor fit (Equation 2, Figure 3, posterior mean deviance = 5856.3—Table 1), as it did not capture the fast rate of decline in WOMAC scores that occurred within the first 2 weeks. A piecewise linear time‐course with a knot at week one showed substantially better model fit (Equation 5, Figure 3, posterior mean deviance = −189.3—Table 1) than the linear or exponential models. However, by far, the best fitting time‐course appeared to be an E_max_ model (Equation 4, posterior mean deviances less than −441—Table 1). Figure 3 shows that whilst the posterior mean contribution to the deviance displays a pattern for the linear and exponential models (suggesting the time‐course is not adequately captured), there is no systematic pattern discernible for the piecewise linear models and best‐fitting E_max_ model (see below), and the deviance contributions are much lower for the E_max_ model than the piecewise linear model.

**Table 1:** Model fit statistics for time‐course models with univariate likelihood, fitted to the osteoarthritis pain dataset. For exchangeable models, the heterogeneity parameter is reported as standard deviation (SD) = posterior mean SD (95% credible interval)

	Model for λ_1,i,k_ (Linear Slope, Exponential Decay, or E_max_)		Model for λ_2,i,k_ (Linear Slope, or ET_50_)				
Time‐course model	Arm 1 effect, μ_1,i_	Relative treatment effects, δ_1,i,k_	Arm 1 effect, μ_2,i_	Relative treatment effects, δ_2,i,k_	DIC^a^	Posterior mean deviance^b^	pD^c^
Linear (*λ*_1,*i*,*k*_= slope)	Unconstrained	Fixed effect			7009.1	6935.2	73.9
Exponential (*λ*_1,*i*,*k*_= decay rate)	Unconstrained	Fixed effect			5931.8	5856.3	75.5
Piecewise linear (*λ*_1,*i*,*k*_= slope period 1, *λ*_2,*i*,*k*_= slope period 2, knot = 0.1 wks)	Unconstrained	Fixed effect	Unconstrained	Fixed effect	−69.1	−189.3	120.2
E_max_ model 1 (*λ*_1,*i*,*k*_= E_max_, *λ*_2,*i*,*k*_= ET_50_)	Unconstrained	Fixed effect	Exchangeable (Equation 12), SD = 0.53 (0.25, 1.10)	Fixed effect *d*_2,1,*k*_ has an exchangeable class effect with an agent‐specific mean, SD = 0.11 (0.01, 0.48)	−274.5	−441.2	166.7
E_max_ model 2 (*λ*_1,*i*,*k*_= E_max_, *λ*_2,*i*,*k*_= ET_50_)	Unconstrained	Fixed effect	Exchangeable (Equation 12), SD = 0.50 (0.24, 0.97)	Fixed effect *d*_2,1,*k*_has a fixed class effect for treatments of same agent	−281.8	−443.1	161.3
E_max_ model 3 (*λ*_1,*i*,*k*_= E_max_, *λ*_2,*i*,*k*_= ET_50_)	Unconstrained	Fixed effect	Exchangeable (Equation 12), SD = 0.61 (0.34, 1.10)	Fixed effect *d*_2,1,*k*_ has an exchangeable class effect with common mean across all treatments, SD = 0.13 (0.01, 0.48)	−284.3	−444.0	159.6
E_max_ model 4 (*λ*_1,*i*,*k*_= E_max_, *λ*_2,*i*,*k*_= ET_50_)	Unconstrained	Fixed effect	Exchangeable (Equation 12), SD = 0.64 (0.39, 1.15)	Fixed effect *d*_2,1,*k*_ has a fixed class effect constrained to be equal for all treatments	−289.9	−441.9	152.0
E_max_ model 5 (*λ*_1,*i*,*k*_= E_max_, *λ*_2,*i*,*k*_= ET_50_)	Unconstrained	Random effects (Equation 10), SD = 0.09 (0.00, 0.23)	Exchangeable (Equation 12), SD = 0.65 (0.39, 1.16)	Fixed effect *d*_2,1,*k*_ has a fixed class effect constrained to be equal for all treatments	−287.9	−448.8	160.8

a DIC (= deviance + pD): It is a measure of model fit that penalises complexity.

b Deviance (= −2(log‐likelihood)): A measure of how closely the fitted values of the model fit the observed data.

c pD: The total number of effective parameters in the model, calculated using the Kullback‐Leibler information divergence.^41^

It was not possible to fit an E_max_ model with separate treatment effects on the ET_50_ parameter, due to the limited number of observations at earlier time points in the dataset. We were therefore obliged to make some simplifying assumptions for ET_50_. We fitted class‐effect models with exchangeable ET_50_ treatment effects (Equation 10) with agent‐specific means (E_max_ model 1) or with a common mean for all treatments (E_max_ model 3). We also fitted class‐effect models with fixed ET_50_ treatment effects (Equation 11) equal to agent‐specific values (E_max_ model 2) or with an equal value for all treatments (E_max_ model 4). For all of E_max_ models 1 to 4, we were also obliged to further assume an exchangeable model for the reference treatment effect (placebo in all studies), $\mu_{{\mathrm{ET}}_{50},i}$ (Equation 12), to allow estimation.

Model fit was similar for E_max_ models 1 to 4, but the model that assumed an equal treatment effect for ET_50_ for all treatments compared with placebo, common across studies (E_max_ model 4), had the lowest DIC.

Step 3. **Compare random and fixed treatment effect models for selected time‐course model**

Table 1 shows model fit statistics for a model that is identical to E_max_ model 4, but with a random treatment effects model for E_max_ parameters (Equation 6) (E_max_ model 5). The between‐study SD for treatment effects on E_max_ was very low (0.09; 95%CrI: 0.00, 0.23), and although model fit was slightly improved compared with the fixed effects model (E_max_ model 4), the added complexity resulted in a higher DIC. There was insufficient data to be able to estimate a bivariate random treatment effects model for E_max_ and ET_50_ parameters (Equation 9). Nonetheless, we provide the JAGS code for this model in the Supporting Information. Based on these results, we select the fixed effects E_max_ model 4.

Step 4. **Assess overall fit of selected univariate model**

The posterior mean residual deviance for E_max_ model 4 was 288.1, which is lower than the number of data points (341), indicating a good fit to the data.

Step 5. **Fit the selected time‐course model using a multivariate likelihood**

Accounting for residual correlation between time points using a multivariate likelihood (Equation 1) for E_max_ model 4 gave an estimated correlation of *ρ* = 0.28 (95%CrI 0.10, 0.41) when using a multivariate compound symmetry covariance structure, and *ρ* = 0.50 (95%CrI 0.19, 0.65) when using a multivariate AR(1) covariance structure (Table 2). Figure S1 (Supporting Information) compares the univariate E_max_ model 4 treatment effects with those from the equivalent multivariate specifications with compound symmetry and autoregressive AR(1) covariance structures. Estimates and their 95% CrIs appear to be reasonably consistent between these models, indicating that accounting for correlation leads to only marginal differences in treatment estimates. Although the differences are very slight, it is interesting to note that use of a multivariate likelihood with compound symmetry covariance structure typically leads to increased precision of treatment estimates compared with the univariate likelihood model, whilst use of a multivariate likelihood with AR(1) covariance structure has more of an effect on the point estimate.

**Table 2:** Model fit statistics for the E_max_ model 4 time‐course model (see Table 1), comparing univariate and multivariate likelihoods, fitted to the osteoarthritis pain dataset. For the exchangeable baseline parameters, standard deviations (SD) are reported as posterior mean SD (95% credible interval). Correlation is reported as posterior mean (95% credible interval)

	Model for λ_1,i,k_ (E_max_)		Model for λ_2,i,k_ (ET_50_)					
Time‐course model	Arm 1 effect, μ_1,i_	Relative treatment effects, δ_1,i,k_	Arm 1 effect, μ_2,i_	Relative treatment effects, δ_2,i,k_	Correlation, ρ	DIC^a^	Posterior mean deviance^b^	pD^c^
E_max_ model 4 univariate likelihood	Unconstrained	Fixed effect	Exchangeable (Equation 12), SD = 0.64 (0.39, 1.15)	Fixed effect, *d*_2,1,*k*_ equal for all treatments	0	−289.9	−441.9	152.0
E_max_ model 4 multivariate likelihood, compound symmetry	Unconstrained	Fixed effect	Exchangeable (Equation 12), SD = 0.68 (0.43, 1.18)	Fixed effect, *d*_2,1,*k*_ equal for all treatments	0.28 (0.10, 0.41)	−266.2	−425.8	159.6
E_max_ model 4 multivariate likelihood, AR(1)	Unconstrained	Fixed effect	Exchangeable (Equation 12), SD = 0.66 (0.41, 1.17)	Fixed effect, *d*_2,1,*k*_ equal for all treatments	0.50 (0.19, 0.65)	−278.9	−437.0	158.1

a DIC (= deviance + pD): It is a measure of model fit that penalises complexity.

b Deviance (= −2(log‐likelihood)): A measure of how closely the fitted values of the model fit the observed data.

c pD: The total number of effective parameters in the model, calculated using the Kullback‐Leibler information divergence.^41^

**Figure 4:**
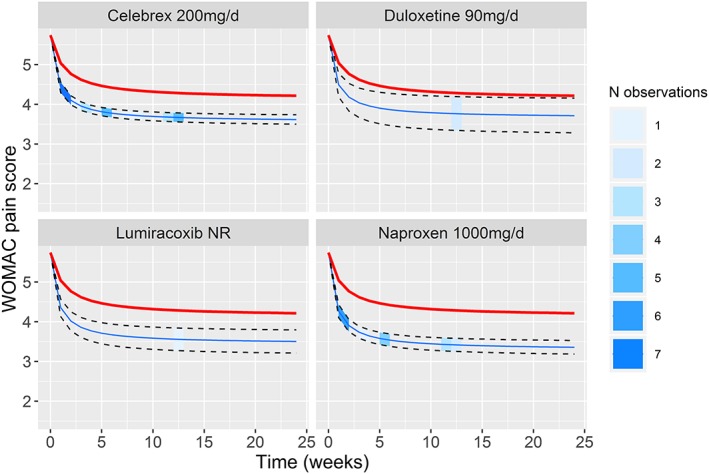
Predicted means and 95% CrI from the final model (E_max_ model 4) for the pain in osteoarthritis dataset for Celebrex 200 mg/d, Duloxetine 90 mg/d, Lumiracoxib (dose not reported), and Naproxen 1000 mg/d, plotted over time. The thicker red line indicates the assumed placebo response (calculated from the data). The shading of the 95% CrI indicates observations present in the dataset at each time point [Colour figure can be viewed at wileyonlinelibrary.com]

**Predictions from the selected model (E_max_ model 4)**

Figure 4, 46 shows the predicted values from E_max_ model 4 for four illustrative treatments (others are given in Figure S2 ‐ Supporting Information). There are many observations for treatments such as Celebrex 200 mg/d and Naproxen 1000 mg/d, providing rich information on the time‐course parameters, whilst for Duloxetine 90 mg/d and Lumiracoxib (NR—dose not reported), the time‐course is largely extrapolated and interpolated.

**AUC for time‐course relationships (E_max_ model 4)**

Table 3 shows the median rank and their 95%CrI from E_max_ model 4 for E_max_ treatment effects for each treatment, and the AUC for each treatment calculated over 24 weeks follow‐up (the maximum latest follow‐up in any of the included studies). As ET_50_ was constrained to be equal across all treatments, the rankings are only dependent on E_max_, and therefore E_max_ rankings match the AUC rankings. Etoricoxib 60 mg/d was the highest median ranked treatment for both AUC and E_max_.

Step 6 **Test for inconsistency**

**Table 3:** Median (95%CrI) rankings (1 = best) for AUC and E_max_ treatment effects for E_max_ model 4. Simplifying assumptions on ET_50_ that constrain it to be equal across all treatments mean that the rankings for AUC are identical to the rankings for E_max_ treatment effects

Treatment	Median AUC Rank (95% CrI)	Median E_max_ Rank (95% CrI)
Etoricoxib 60 mg/d	1 (1, 3)	1 (1, 3)
Etoricoxib 90 mg/d	2 (1, 4)	2 (1, 4)
Rofecoxib 125 mg/d	3 (1, 6)	3 (1, 6)
Etoricoxib 30 mg/d	4 (3, 12)	4 (3, 12)
Oxycodone 44 mg/d	5 (1, 25)	5 (1, 25)
Rofecoxib 25 mg/d	6 (4, 15)	6 (4, 15)
Naproxcinod 1500 mg/d	7 (5, 11)	7 (5, 11)
Naproxen 1000 mg/d	10 (6, 14)	10 (6, 14)
Celebrex 400 mg/d	11 (6, 21)	11 (6, 21)
Etoricoxib 10 mg/d	12 (5, 27)	12 (5, 27)
Naproxcinod 750 mg/d	13 (7, 23)	13 (7, 23)
Etoricoxib 5 mg/d	14 (5, 28)	14 (5, 28)
Lumiracoxib ( not reported)	14 (7, 24)	14 (7, 24)
Valdecoxib 20 mg/d	15 (6, 25)	15 (6, 25)
Rofecoxib 12 mg/d	16 (7, 25)	16 (7, 25)
Lumiracoxib 100 mg/d	17 (11, 23)	17 (11, 23)
Lumiracoxib 400 mg/d	17 (10, 24)	17 (10, 24)
Tramadol 300 mg/d	17 (8, 24)	17 (8, 24)
Valdecoxib 10 mg/d	17 (7, 26)	17 (7, 26)
Celebrex 200 mg/d	18 (13, 23)	18 (13, 23)
Lumiracoxib 200 mg/d	19 (12, 24)	19 (12, 24)
Valdecoxib 5 mg/d	19 (8, 26)	19 (8, 26)
Tramadol 400 mg/d	20 (8, 27)	20 (8, 27)
Duloxetine 90 mg/d	22 (8, 28)	22 (8, 28)
Celebrex 100 mg/d	25 (17, 27)	25 (17, 27)
Tramadol 200 mg/d	25 (17, 27)	25 (17, 27)
Tramadol 100 mg/d	27 (22, 28)	27 (22, 28)
Placebo 0 mg/d	28 (27, 29)	28 (27, 29)
Naproxcinod 250 mg/d	29 (26, 29)	29 (26, 29)

In the osteoarthritis dataset, all studies included a placebo arm. Within the contrast‐based NMA approach, the relative effects within a study are only estimated for each treatment versus the study reference treatment—it is not necessary to estimate relative effects between nonreference treatments within a multi‐arm study because these will be defined by the difference between the relative effects for each treatment versus the study reference, as each study must be internally consistent. Therefore, there were no closed loops of treatments in the network that were made up of independent sources of evidence, and as a result it was not possible to test for inconsistency (Figure S3A ‐ Supporting Information).

For illustrative purposes, to create a dataset in which it is possible to test for inconsistency, we expanded our inclusion criteria to all studies irrespective of their method of imputation. This added an extra seven studies to create an “augmented dataset” (30 studies in total). The augmented dataset is freely available in the Supporting Information (AugmentedInconsistencyData.csv).^44^ One of these additional studies^47^ compared Celebrex 200 mg/d, Rofecoxib 25 mg/d, and Naproxen 100 mg/d but not Placebo. This created two loops in the network in which direct and indirect estimates came from independent sources, meaning it was possible to test for inconsistency (Figure S3B ‐ Supporting Information).

In the augmented dataset, results from the UME model were very similar to the MBNMA model (E_max_ model 4). Posterior mean residual deviance was 374.0 for the UME model compared with −370.3 for the MBNMA model, whilst the between‐study SD for the reference treatment effect for ET_50_ was almost identical for both UME (0.69 (95%CrI: 0.43, 1.18) and MBNMA models (0.69 (95%CrI: −0.44, 1.18). There is therefore no evidence to invalidate the consistency assumption.

A node splitting model was fitted for the two closed loops of treatments in the network that comprised independent data sources, giving two comparisons on which to node split (Figure S3B ‐ Supporting Information).

**Figure 5:**
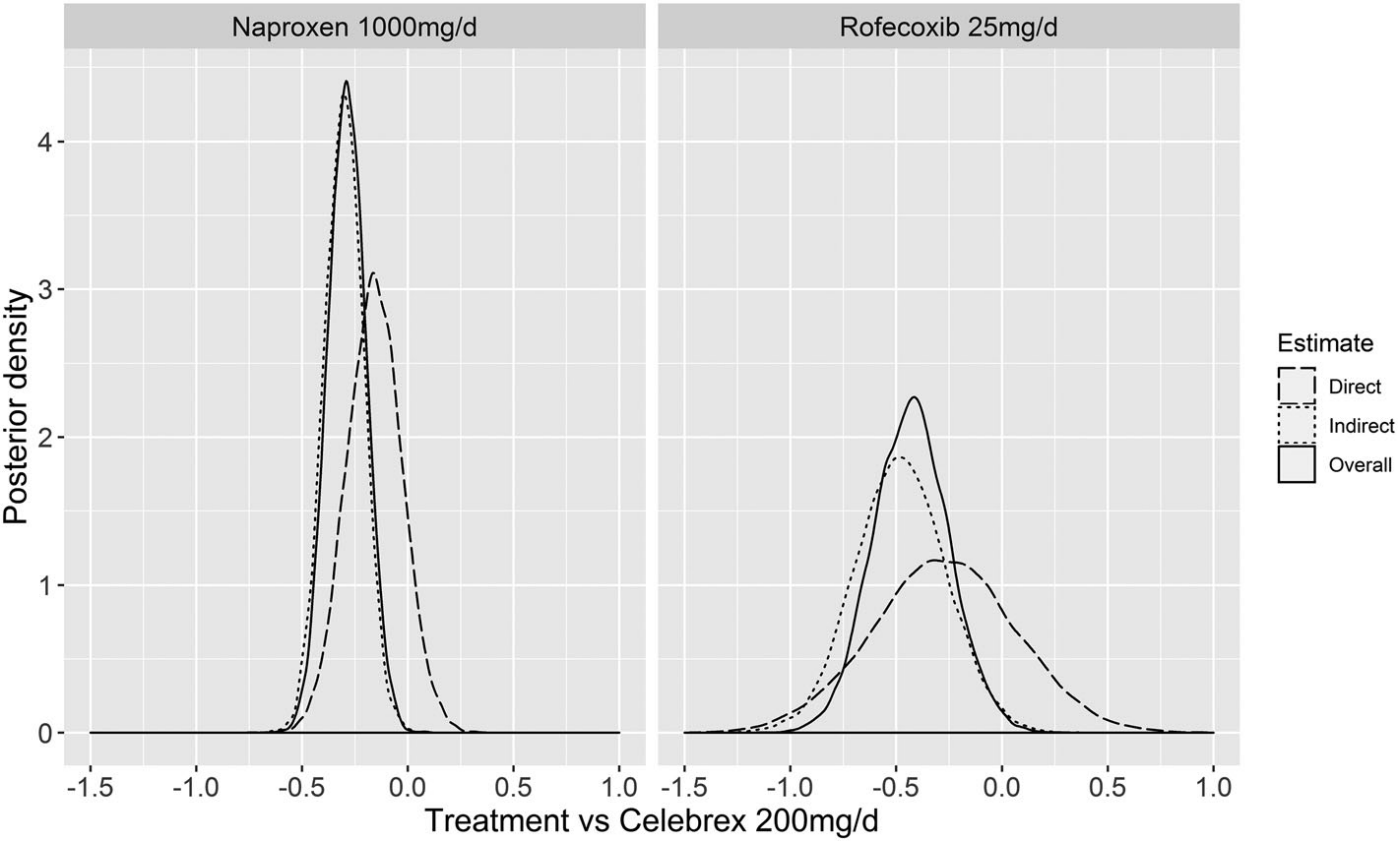
Posterior densities for the effect of naproxen (1000 mg/d) versus Celebrex (200 mg/d) and Rofecoxib (25 mg/d) versus Celebrex (200 mg/d) on E_max_ for the direct and indirect evidence arising from node splitting when testing for inconsistency using E_max_ model 4 for the augmented dataset. Bayesian P‐value of 0.69 and 0.79, respectively, representing the proportion of the densities that overlap

For both comparisons, the MBNMA estimate was effectively a weighted average of the direct and indirect estimates, as would be expected, though in both the indirect evidence is more precise and therefore has the greatest influence on the MBNMA result (Figure 5). The Bayesian *P*‐value representing the overlap of the posterior distributions for the direct and indirect evidence was 0.69 for Celebrex 200 mg/d vs Naproxen 1000 mg/d and 0.79 for Celebrex 200 mg/d vs Rofecoxib 25 mg/d indicating no evidence of inconsistency in either loop of treatments.

### 3.1 Model checking

For all models for which results are reported, Gelman's r statistic and visual inspection of the chains were indicative of convergence. Convergence diagnostic plots for the final model (E_max_ model 4) and for a nonconverging “ideal” E_max_ model with no simplifying assumptions on ET_50_ are given in the Supporting Information.

Posterior densities were not constrained or strongly influenced by priors. In the final model (E_max_ model 4), the SD for the exchangeable study reference treatment effect was 0.64 (95%CrI: 0.39, 1.15) when using a uniform (*U*(0,5)) prior and 0.64 (95%CrI: 0.39, 1.16) when using a half‐normal () prior (Figure S4 ‐ Supporting Information). For the random E_max_ treatment effects model (E_max_ model 5), the between‐study SD for random E_max_ treatment effects was 0.09 (95%CrI: 0.00, 0.23) when using either a uniform (*U*(0,5)) prior or a half‐normal () prior, suggesting the choice of prior is not influential.

## 4 DISCUSSION

We have presented a method to pool evidence from trials that form a network of comparisons across multiple treatments, reporting continuous summary outcome measures over multiple time points. The method respects the randomisation in RCTs, can include a variety of different functional forms for the time‐course, allows for testing of consistency of the evidence, and demonstrates how a multivariate likelihood can be used to account for residual correlation between time points.

In the pain in osteoarthritis dataset, we found that the E_max_ model provided the best fit and allowed for the greatest degree of flexibility, both in the time‐course shape and in the specification of various time‐course parameters (E_max_ and ET_50_). The E_max_ model with the lowest DIC (E_max_ model 4) assumed a fixed effect across all nonplacebo treatments on ET_50_ (Equation 12). In this model, we estimated AUC of the time‐course function over 24 weeks follow‐up and found Etoricoxib 60 mg/d to have the highest ranked AUC.

Whilst WOMAC pain in this dataset was measured as a continuous outcome, MBNMA can also be performed on any data provided it can be *summarised* as a continuous outcome that can be assumed to have a normal likelihood. This therefore allows for binary data (ie, % respondents at each follow‐up time) to be analysed using MBNMA if they are summarised as log‐odds and are not near the boundary probabilities (0% or 100%).

### 4.1 Time‐course function

Previous methods for performing NMA on longitudinal data have typically accounted for the dependency between different follow‐up times by either modelling an appropriate time‐course function^18^, ^19^ or using nonparametric approaches to account for responses at each time point.^21^, ^34^ To our knowledge, none of the methods for modelling longitudinal continuous data in NMA have described methods for testing inconsistency.

Jansen et al^19^ used fractional polynomials to model a nonlinear trend, with an inflated variance for each time point to approximate correlations between observations. These allow for a very flexible shape for modelling the time‐course relationship. However, fractional polynomials are difficult to interpret and may not have a clear biological justification, making them less desirable for use in pharmacometric studies, where the objective is to define how the efficacy of a treatment changes over time rather than to simply account for it. For modelling time‐course in pharmacometrics, exponential and E_max_ functions are typically used, as these have a biological basis in describing the mechanism of drug action and elimination from the body.^48^ Taking this into account, Ding and Fu^18^ modelled an exponential shape in NMA and described how their model could be adapted for a sigmoid shape similar to that of an E_max_ function.

Nonparametric models that do not specify a particular time‐course relationship have also been proposed, although these do not allow for any interpolation or extrapolation of treatment effects at unmeasured points in time, which makes them less applicable to drug development. Ishak et al^21^ used a multivariate likelihood to account for the dependency between different time points. Dakin et al^34^ used a saturated model to estimate treatment effects separately for different time bins, although for this method more data are required to reliably estimate the treatment effects for each bin.

One of the strengths of our MBNMA framework is that information on time‐course characteristics can be inferred from other treatments or agents by assuming varying degrees of similarity and thus improving identifiability. However, sharing information on time‐course parameters across a network assumes exchangeability, and it is important to be aware of the implications of this assumption and to consider whether it holds across different agents or classes of treatments.

### 4.2 Correlation between time points

Having selected an appropriate time‐course relationship in the pain in osteoarthritis dataset, we found that estimated residual correlation was reasonably high. This was in contrast to our expectation that explicitly modelling the time‐course should have generated conditional independence. However, accounting for this correlation had only a slight impact on treatment effect estimates or 95% CrIs. This suggests that for MBNMA where the focus is on summary estimates, whilst accurately characterising the within‐study correlation and covariance structure may be important, it is likely to be less critical than accurately characterising the time‐course. With regards to the importance of modelling within‐study correlation, there is some debate in the literature. A simulation study by Ishak et al^49^ suggests that the impact of ignoring within‐study correlation on treatment estimates may typically be small, even in cases where there has been no specific modelling of a time‐course function. However, Riley^50^ has shown that this is only the case when between‐study variation is large relative to within‐study variation, or when there are complete data with only small differences in the within‐study covariance matrices across studies. Ahn and French^2^ support this position, demonstrating that ignoring correlation in longitudinal MBMA led to inflated residual variance. We are currently performing a simulation study to further examine the relationship between time‐course fit and correlation in MBNMA.

Note that the estimated covariance matrix in the multivariate likelihood will depend on the time‐course model fitted, and the strength and importance of correlations between time points are also likely to depend considerably on how close together follow‐up measurements are in time, with closer measurements expected to be more strongly correlated.

Previous longitudinal MBMA methods that account for correlated residuals have been developed in a frequentist framework, using NONMEM software to allow for modelling of interarm variability in addition to interstudy variability.^2^ The authors used an exponential model for the time‐course and also accounted for nonlinear dose‐response in their model. Although we follow a Bayesian approach and our model is formulated somewhat differently, our multivariate model with a compound symmetry covariance structure is similar to their method. However, the key difference is that our approach respects randomisation and allows for inconsistency testing. Without these features, the methods are unlikely to meet the requirements of reimbursement agencies.

### 4.3 Modelling assumptions

For our selected E_max_ model in the pain in osteoarthritis dataset (E_max_ model 4), assuming a fixed effect across all nonplacebo treatments for ET_50_, whilst allowing all treatment effects to be different for E_max_ implies that the onset of action is the same for the different treatments relative to placebo, but that treatments differ in the maximal change in outcome achieved. In practice, this might be considered an unusual modelling assumption, as one might expect ET_50_ to differ between active treatments, particularly for those acting via different biological pathways. However, the onset for all these treatments was very rapid, and there were insufficient observations at early time points to reliably estimate this. In fact, none of the included studies report WOMAC scores within a week from baseline. Given that this is later than the estimated ET_50_ (approximately 0.6 weeks), it is not surprising that this parameter is difficult to estimate. For this example, we would therefore caution against making inferences at very early time points. Using informative prior distributions for the $\tau_{{\mathrm{ET}}_{50}}^{2}$ or for $d_{{\mathrm{ET}}_{50},1,k}$ parameters for which information is sparse may improve estimation. Information from noncomparative pharmacodynamic studies of different agents may be useful to provide support for specific prior distributions. Another approach may be to incorporate information from other treatments in a more biologically plausible manner, such as by using the dose‐response relationships between treatments within an agent.^17^

Explicitly modelling the bivariate correlation between E_max_ and ET_50_ may in some circumstances also provide additional information to help identify ET_50_ and reduce the need for such strong simplifying assumptions. This is likely to be the case when correlation between E_max_ and ET_50_ is high. However, in the pain in osteoarthritis example, this still was not sufficient to help identify ET_50_, even when alternative parameterizations for the covariance matrix were used.^31^

Within time‐course MBNMA, it is necessary to assume the same time‐course function for the included set of treatments. Whilst the mean responses over time for most treatments supported the use of an E_max_ function, there were only two observations (baseline and one follow‐up measurement) for studies comparing Lumiracoxib (NR—dose not reported) vs Placebo and Duloxetine 90 mg/d vs Placebo.

For Lumiracoxib NR, it was reasonable to assume that the time‐course function will be similar to other doses of Lumiracoxib for which there are more observations. However, there are no other doses of Duloxetine to make an equivalent assumption, and as the mechanism of Duloxetine is also different to any other agent in the dataset, it may follow a different time‐course function. Yet, as this treatment did not contribute any indirect evidence to the rest of the network (which could induce bias in other treatment estimates if modelled appropriately) and there was no evidence to suggest a different time‐course function would be applicable for this treatment, we feel it is reasonable to assume a similar time‐course function to other treatments provided treatment effect estimates for Duloxetine 90 mg/d are interpreted with caution. This case highlights the importance of understanding the underlying pharmacometrics of treatments in the data, and of dialogue between clinicians, pharmacometricians, and analysts.

An additional assumption made in all the E_max_ models due to the inclusion of Lumiracoxib NR and Duloxetine 90 mg/d was that of an exchangeable placebo (Equation 12), as the lack of multiple follow‐up measurements made separate estimation of all three parameters for the time‐course (E_0_, ET_50_, and E_max_) impossible for these comparisons. This is likely to have caused a certain degree of shrinkage and may therefore induce bias in treatment estimates for ET_50_ due to back‐propagation of the information on the reference treatment.^9^

### 4.4 Inconsistency

In NMA, two approaches are typically used for dealing with longitudinal studies. A single consistent time point may be used for analysis across studies, ignoring evidence from other time points. Alternatively, the final time point from studies with different follow‐up times may be “lumped” together to allow for networks to be connected, yet this lumping can often be a source of inconsistency and/or heterogeneity.^16^ Whilst MBNMA solves the issue of lumping together studies with different follow‐up times or discarding information on multiple time points, the choice of model will affect the presence of inconsistency. We suspect that a poorly fitting time‐course model may induce inconsistency. It is therefore important to explore different functional forms and identify a good model *before* testing for inconsistency.

For the purposes of drug development, the potential for inconsistency testing in MBNMA may in fact be relatively rare. The typical design of Phase II trials is multi‐arm placebo‐controlled, meaning that there are no closed loops of treatments that are not made up of multi‐arm trials (as in the illustrative osteoarthritis dataset). As these trials must inherently be internally consistent, this provides no means to test for inconsistency. However, we are still relying on the consistency assumptions to make indirect comparisons, so although in these cases we cannot formally test for inconsistency it is important to consider whether these assumptions are valid.

For the pain in osteoarthritis augmented dataset, we did not find any evidence of inconsistency in the augmented dataset when including non‐LOCF studies, and parameter estimates were robust to their inclusion. In practice, we would recommend careful consideration of inclusion criteria to ensure only studies on which the consistency assumption is expected to hold are included.^13^

It is worth noting that a standard NMA performed by “lumping” the latest time point in each of the studies (an approach frequently used but not one that we would recommend) highlights the benefit of performing MBNMA when dealing with different follow‐up times. In terms of DIC, a random treatment effects NMA was preferred over a fixed treatment effects NMA, with a nonzero between‐study SD (0.25 (95%CrI: 0.17, 0.36)). A Bayesian *P*‐value for the node‐split of Celebrex 200 mg/d vs Naproxen 1000 mg/d was 0.011 and for Celebrex 200 mg/d vs Rofecoxib 25 mg/d was 0.100 suggesting reasonable evidence of inconsistency in both comparisons that was particularly concerning given that the direct and indirect evidence for both showed opposite directions of effect. Therefore, by accounting for time‐course using MBNMA, we have explained heterogeneity and inconsistency that can arise when using standard NMA methodology.

### 4.5 Limitations

There are a few limitations to the methodology that we seek to investigate further in simulation studies. The first is that the quantity of data required for MBNMA may be significant, particularly for more complex time‐course functions. This therefore means that analyses may typically require strong simplifying assumptions within the modelling that are difficult to test. This could relate to the assumption that time‐course functions are the same across all treatments within the network, but also to the need to interpolate or extrapolate over the time‐course when few observations are available for a particular treatment.

We also do not fully understand the importance of correctly accounting for the correlation between time points, and how failure to do this might affect estimates for different parameters in the model. We believe that the impact of this is likely to depend on the key parameters of interest in the model. Simulation can help to shed light on this issue.

### 4.6 Future direction

In future work, we plan to incorporate simultaneous dose‐response and time‐course modelling into the MBNMA framework,^17^ and to develop simulation studies to explore the robustness and data requirements of dose‐response, time‐course, and methods for assessing inconsistency in MBNMA. We are also developing an R package for MBNMA to facilitate its implementation.

## 5 CONCLUSION

MBNMA combines the strengths of both MBMA and NMA, leading to a statistically robust framework for synthesising evidence on multiple treatments at multiple time points whilst preserving randomisation and allowing for assessment of consistency. By unifying these statistical techniques, the methods can provide both the information needed to inform drug‐development decisions, and also the rigour required by reimbursement agencies to incorporate valuable evidence from drug development into the decision‐making process.

### 5.1 Highlights

Within drug development, MBMA is increasingly used to inform drug decisions such as whether to proceed to further clinical trials, and if so, what the design of the study should be. However, these types of analyses typically model each drug separately by pooling individual study arms, which breaks the randomisation and ignores within‐study comparisons, effectively losing the advantages of the RCT design and potentially inducing bias in the resulting estimates.

Our MBNMA framework preserves randomisation by modelling relative effects and allows for testing of inconsistency between direct and indirect evidence. This manuscript extends previous methodology on dose‐response MBNMA to allow the modelling of nonlinear time‐course characteristics, incorporating multiple study time points and accounting for correlation between them.

MBNMA combines techniques from two different disciplines, pharmacometrics and evidence synthesis, thereby acting as a bridge between early phase clinical research and Health Technology Appraisal.

#### Glossary

Agent = an intervention/compound/drug

Identifiability = the capacity for parameters in a model to be reliably estimated

Treatment = a specific dose and agent combination.

## CONFLICT OF INTEREST

The author reported no conflict of interest.

## DATA AVAILABILITY STATEMENT

The data that support the findings of this study are openly available in “figshare” at https://doi.org/10.6084/m9.figshare.8138045.v1.

## Supporting information

Data S1:
Supporting Information

Data S2: Supporting Information

Data S3: Supporting Information

Data S4: Supporting Information

Data S5: Supporting Information

Data S6: Supporting Information

Data S7: Supporting Information

Data S8: Supporting Information

Data S9: Supporting Information

Data S10: Supporting Information

Data S11: Supporting Information

Data S12: Supporting Information

Data S13: Supporting Information

Data S14: Supporting Information

Data S15: Supporting Information

Data S16: Supporting Information

Data S17: Supporting Information

Data S18: Supporting Information

Data S19: Supporting Information

Data S20: Supporting Information

Data S21: Supporting Information

Data S22: Supporting Information

Data S23: Supporting Information

Data S24: Supporting Information

Data S25: Supporting Information

Data S26: Supporting Information

Data S27: Supporting Information

Data S28: Supporting Information

Data S29: Supporting Information

Supplementary Figure 3: Illustrates the potential for testing for consistency in the original dataset comprised entirely of placebo‐controlled trials (A) and in the augmented dataset including one study comparing Celebrex 200 mg/d, Naproxen 1000 mg/d, and Rofecoxib 25 mg/d (B). Only contrasts for which within‐study relative effects (delta [i,k]) are estimated are included in the diagrams. Connected lines indicated multi‐arm studies. For ease of illustration, contrasts for other treatments in the network have not been included here. The single contrasts for each active treatment versus placebo therefore can represent either a two‐arm study or a larger multi‐arm study including other treatments. Other treatments are not shown here as this would not affect our overall potential for inconsistency testing in these selected treatments.

Data S31: Supporting Information

Data S32: Supporting Information
