# Supplementary material for: Modelling time‐course relationships with multiple treatments: Model‐based network meta‐analysis for continuous summary outcomes
Source: Res Synth Methods. 2019 May 29;10(2):267–86. doi: 10.1002/jrsm.1351 (PMC6563489; doi:10.1002/jrsm.1351)

Convergence diagnostics for model with consistency equations on Emax and ET50

Table of Contents

- d.Emax
- d.ET50
- deviance

Plots for d.Emax

d.Emax[1]. Error in bw.SJ(x, method = "ste"): sample is too sparse to find TD

Diagnostics for d.Emax[2]

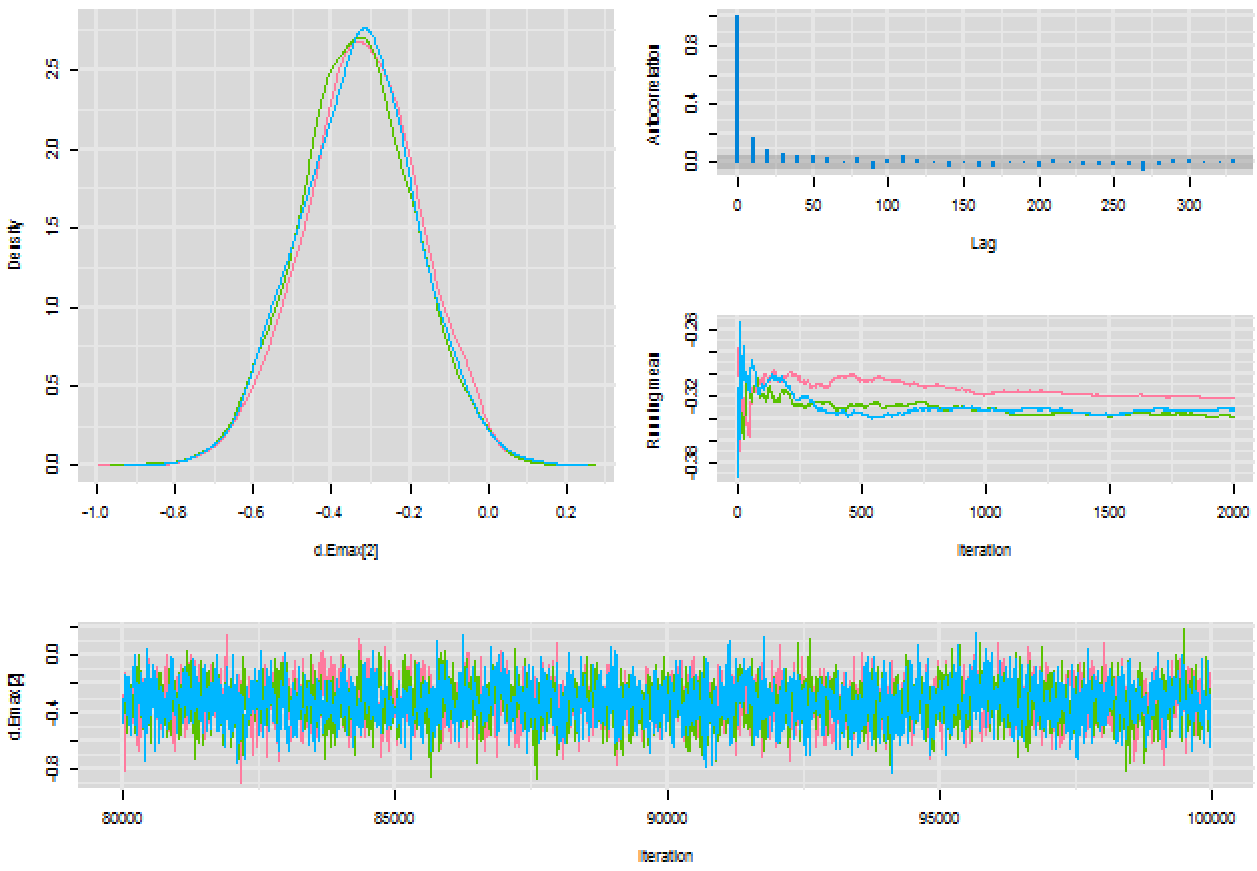

Diagnostics for d.Emax[3]

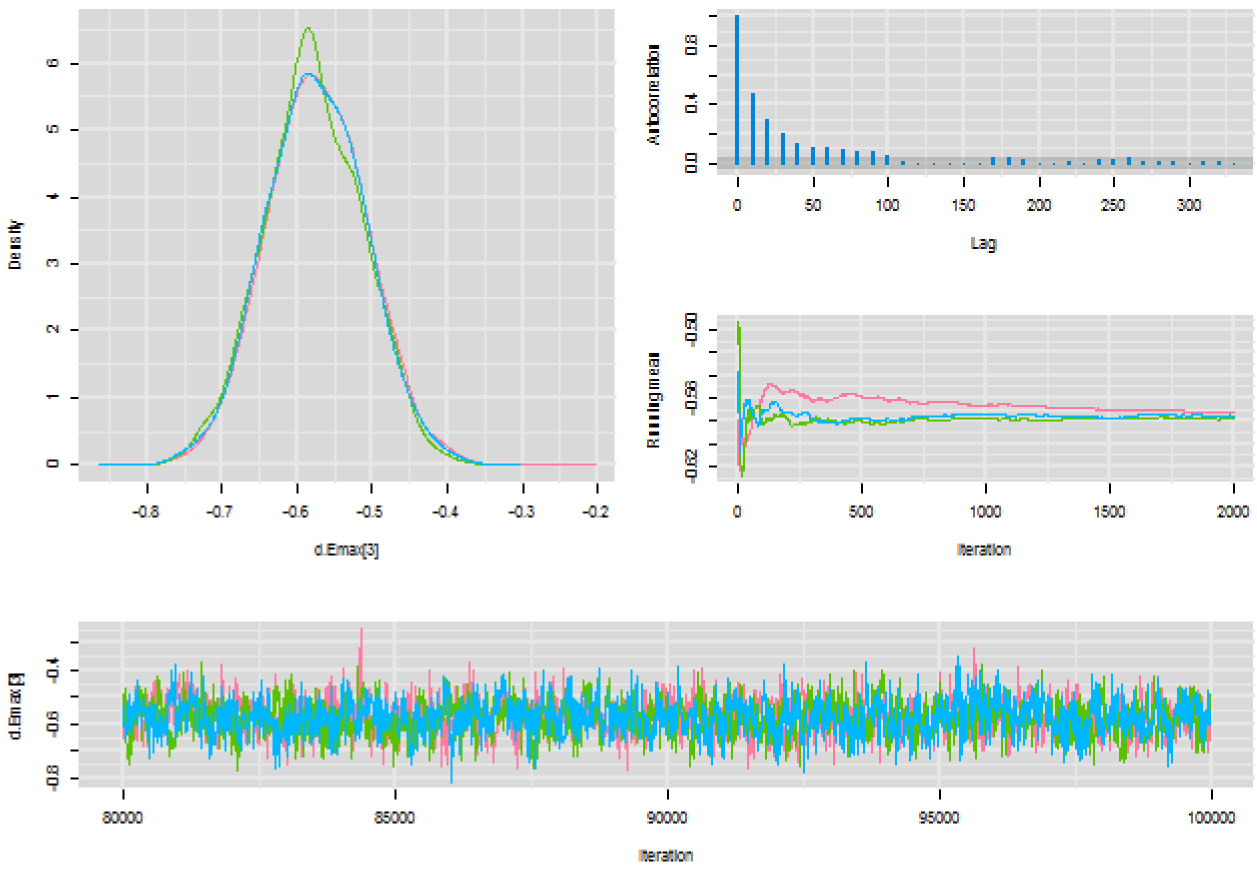

Diagnostics for d.Emax[4]

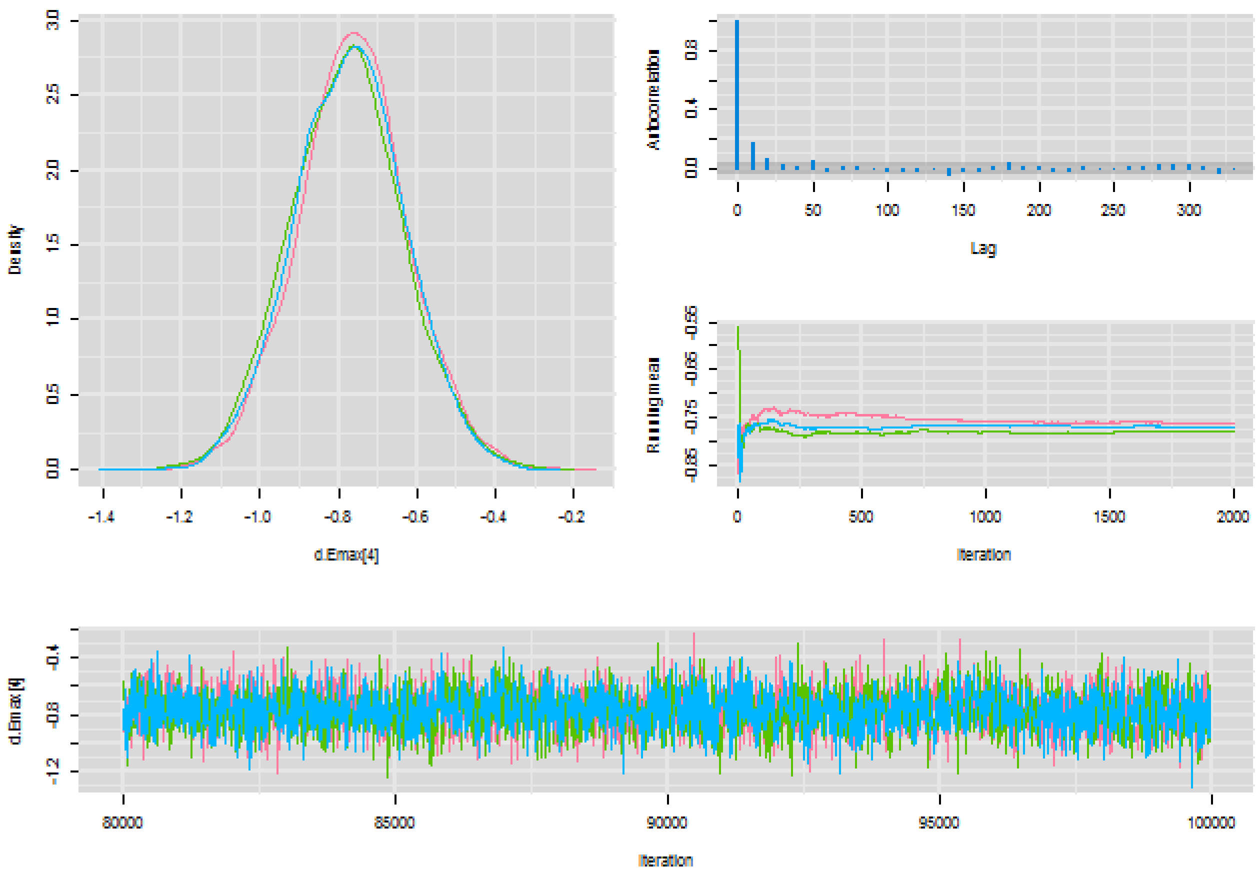

Diagnostics for d.Emax[5]

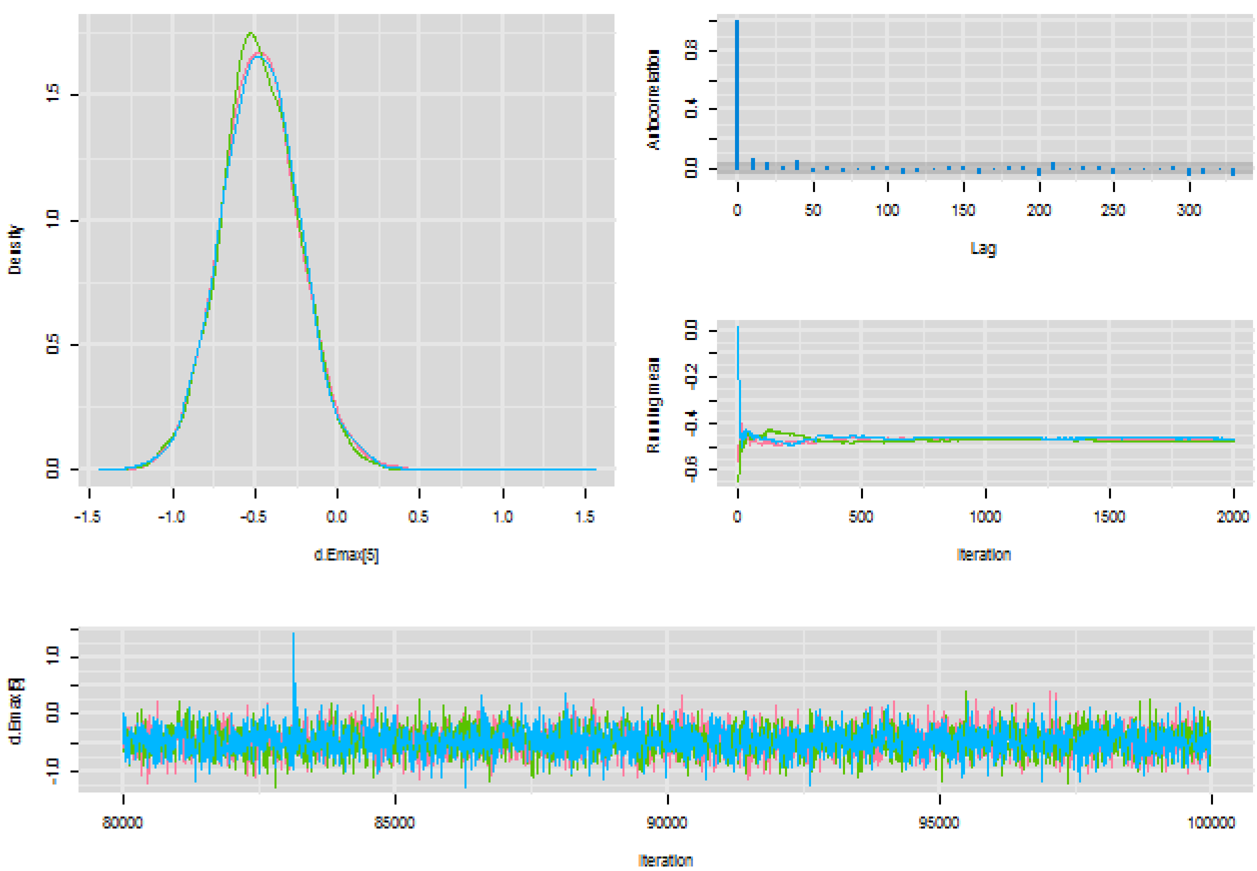

Diagnostics for d.Emax[6]

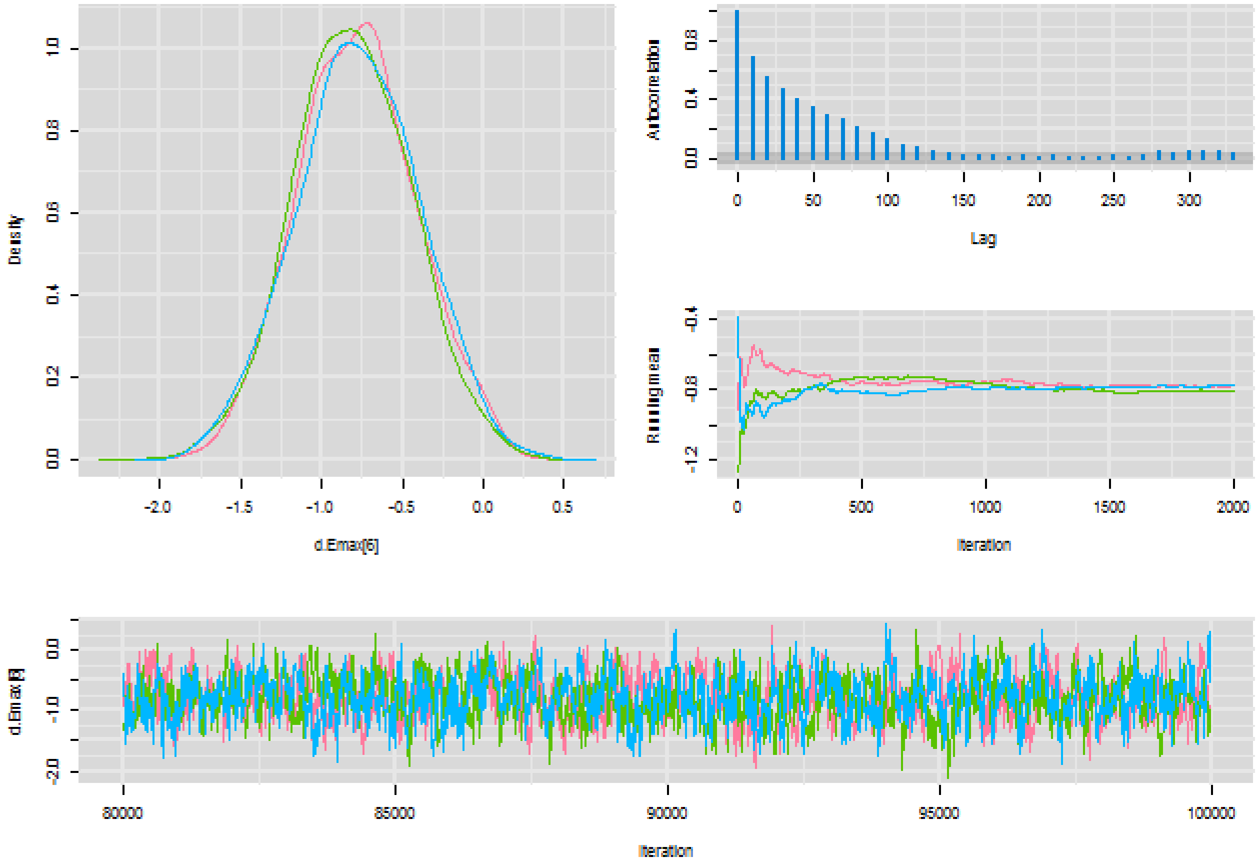

Diagnostics for d.Emax[7]

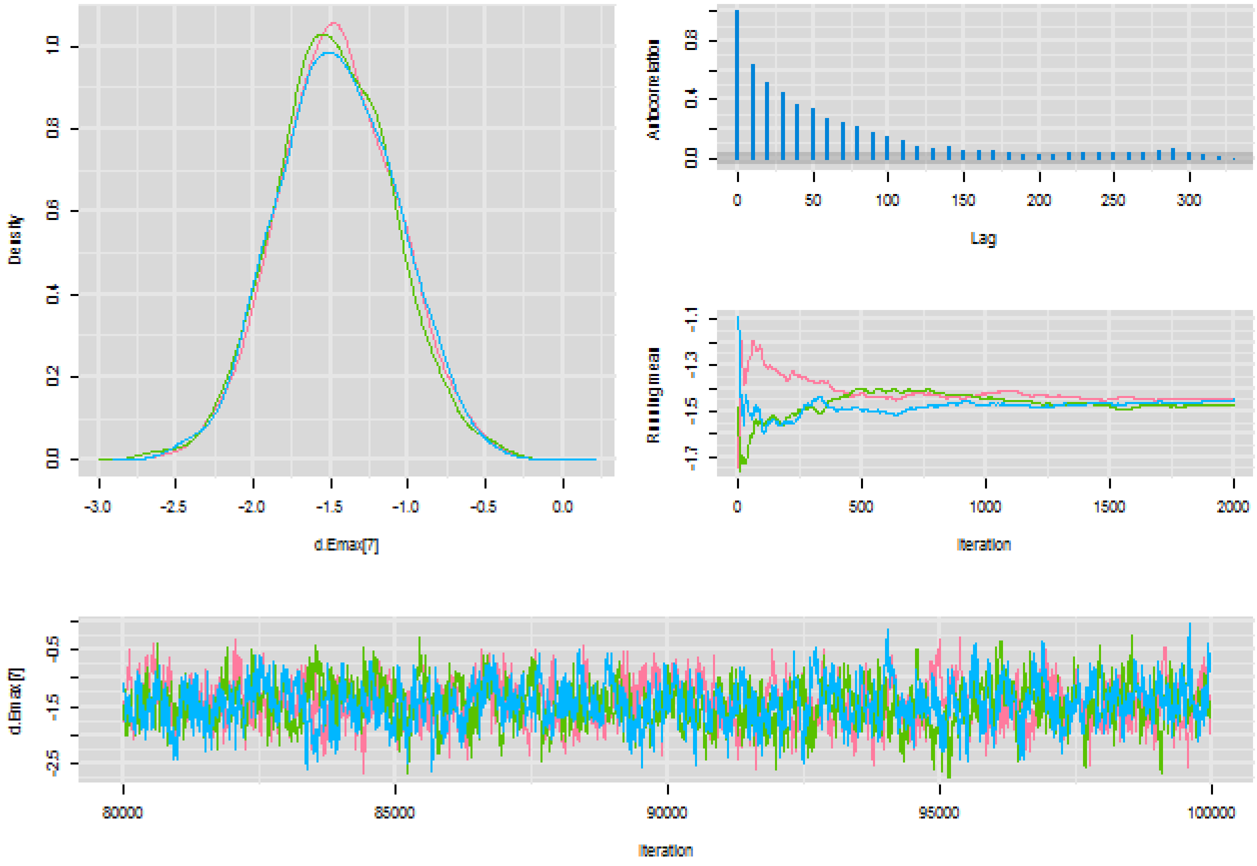

Diagnostics for d.Emax[8]

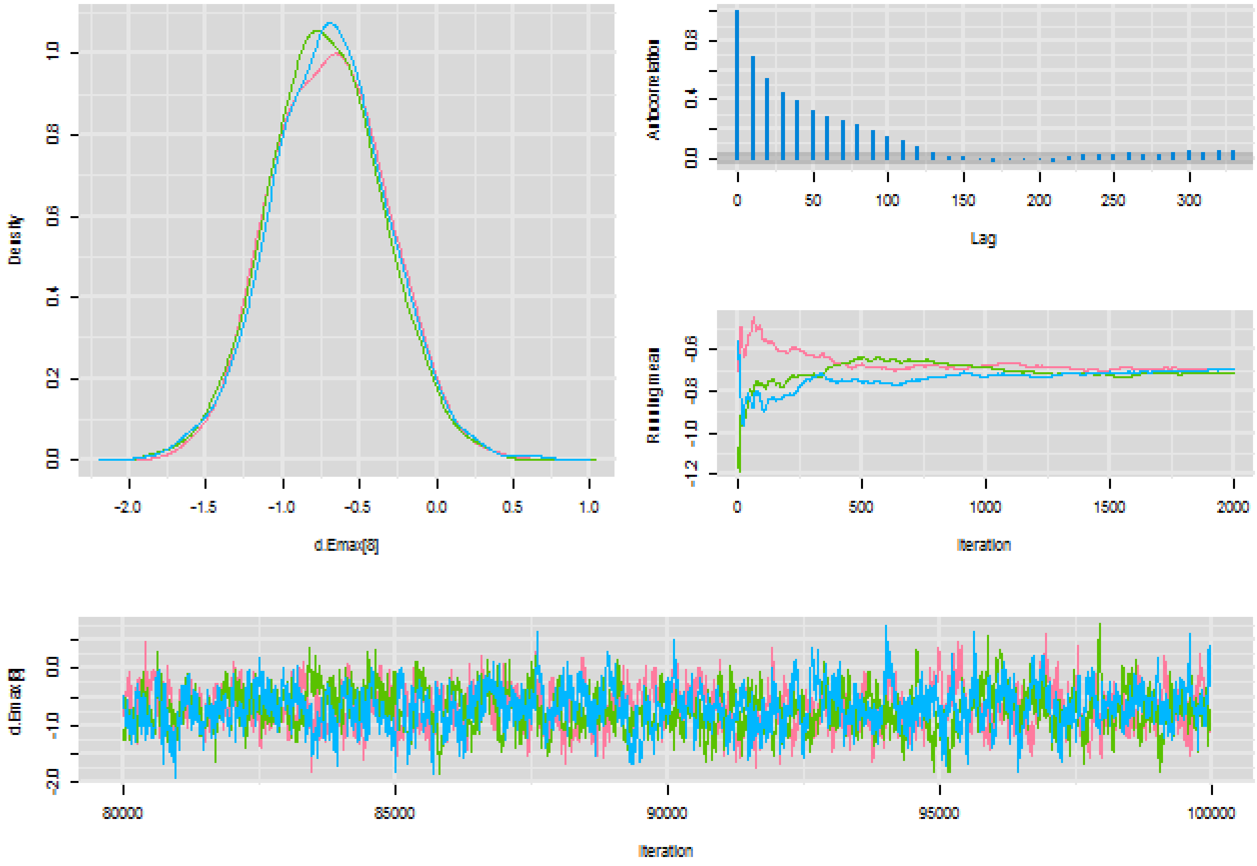

Diagnostics for d.Emax[9]

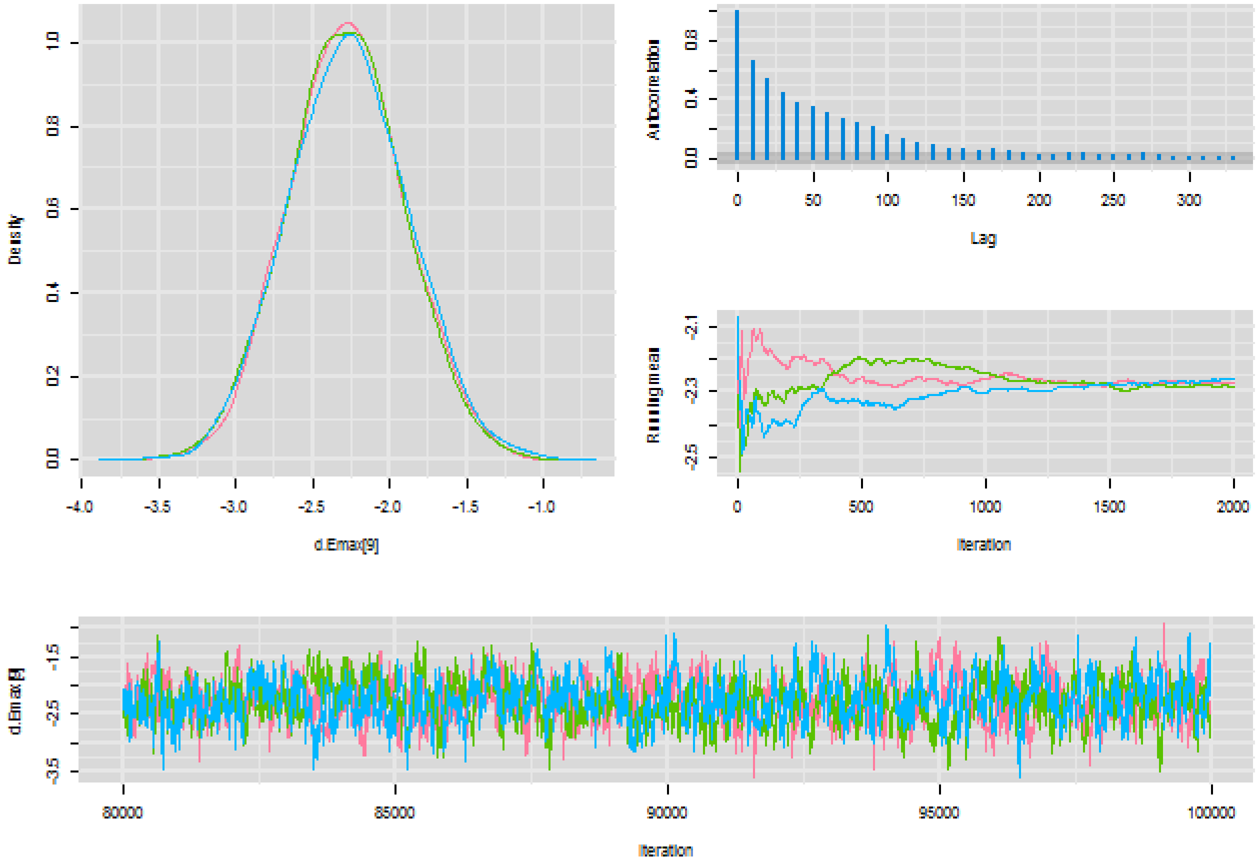

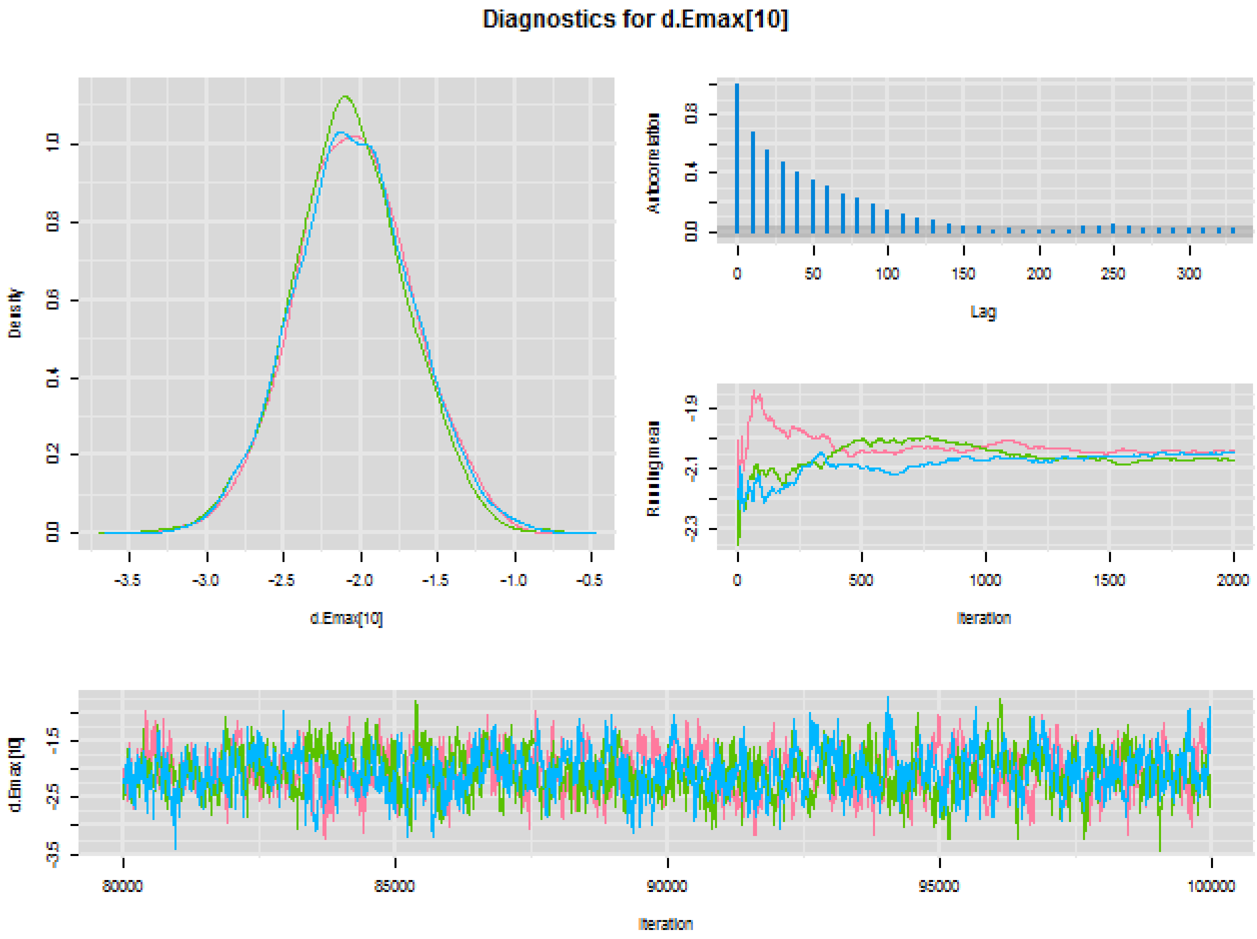
Diagnostics for d.Emax[10]
Density
d.Emax[10]
Autocorrelation
Lag
Running mean
Iteration
d.Emax[10]
Iteration

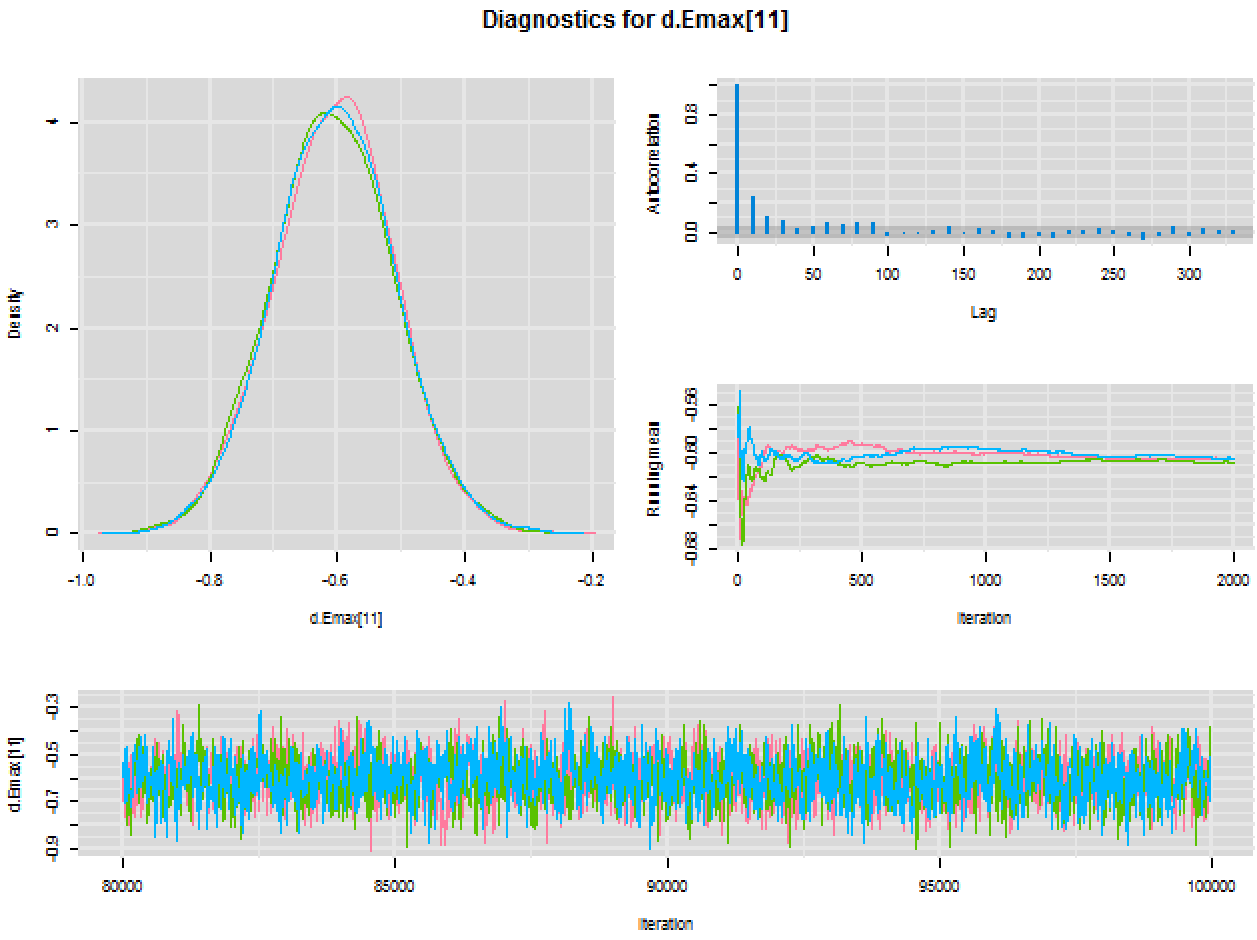
Diagnostics for d.Emax[11]
Density
d.Emax[11]
Autocorrelation
Lag
Running mean
Iteration
d.Emax[11]
Iteration

Diagnostics for d.Emax[12]

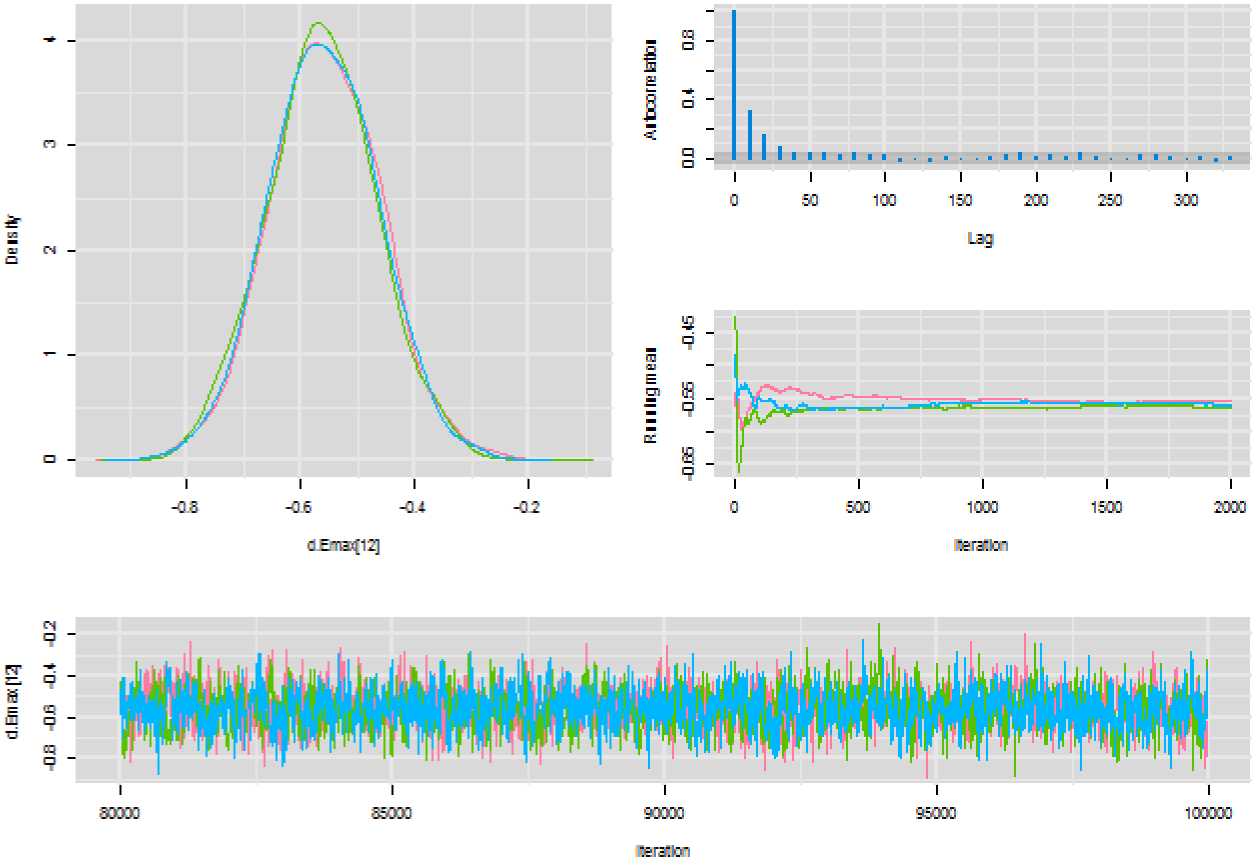

Diagnostics for d.Emax[13]

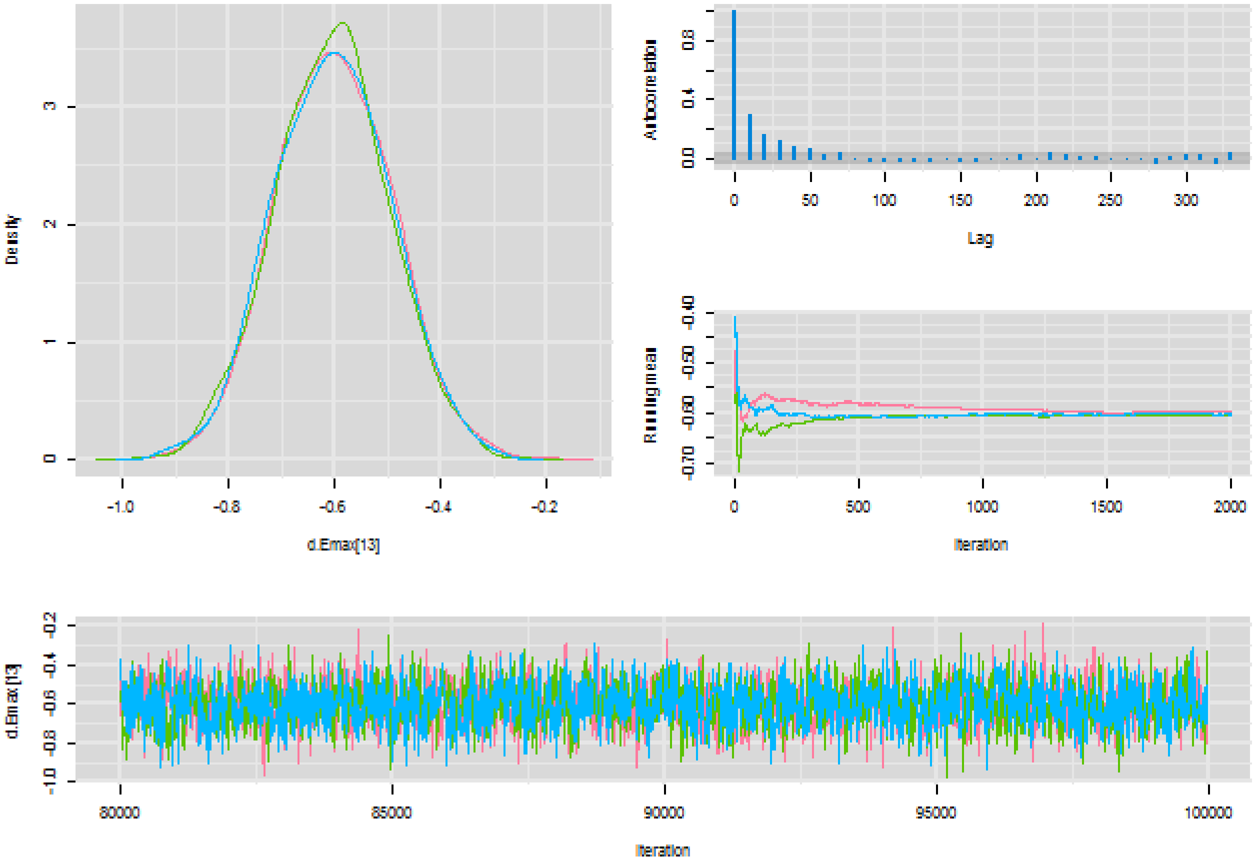

Diagnostics for d.Emax[14]

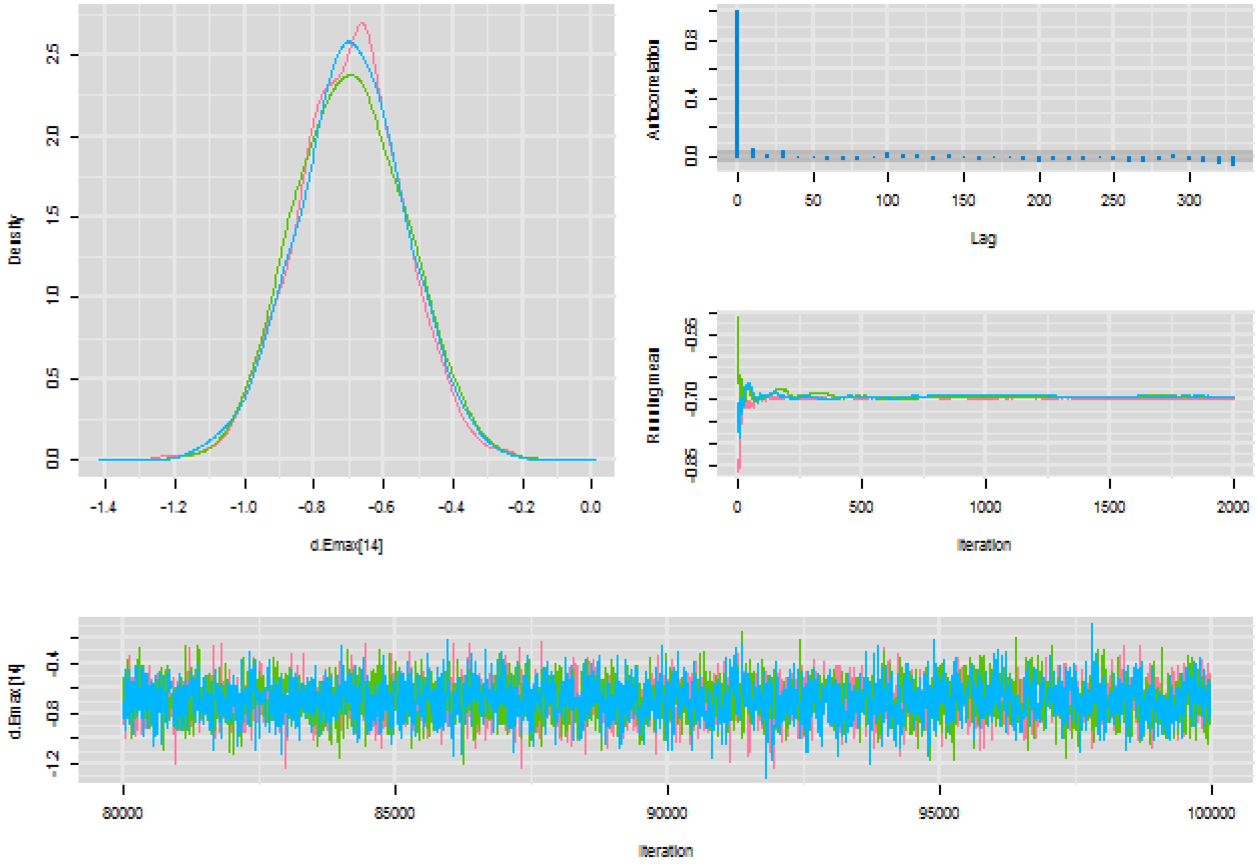

Diagnostics for d.Emax[15]

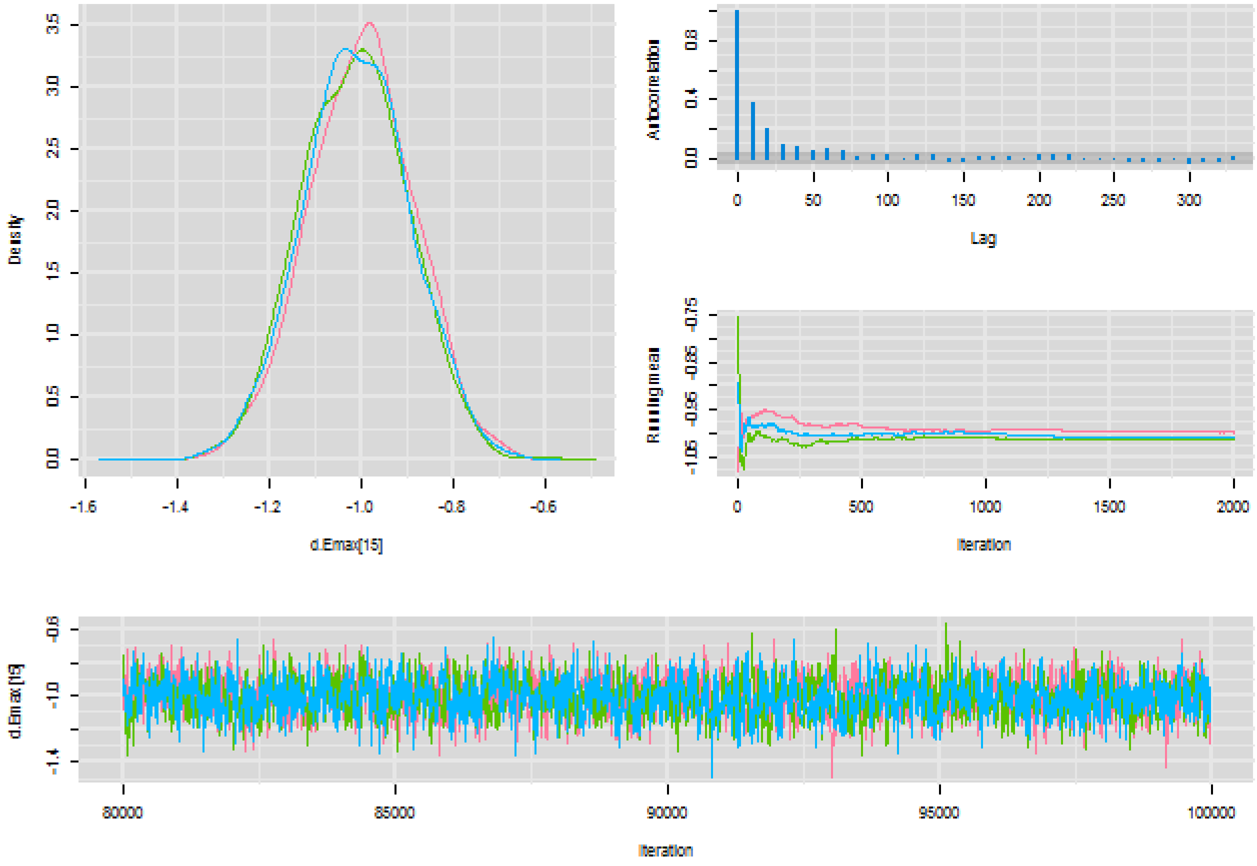

Diagnostics for d.Emax[16]

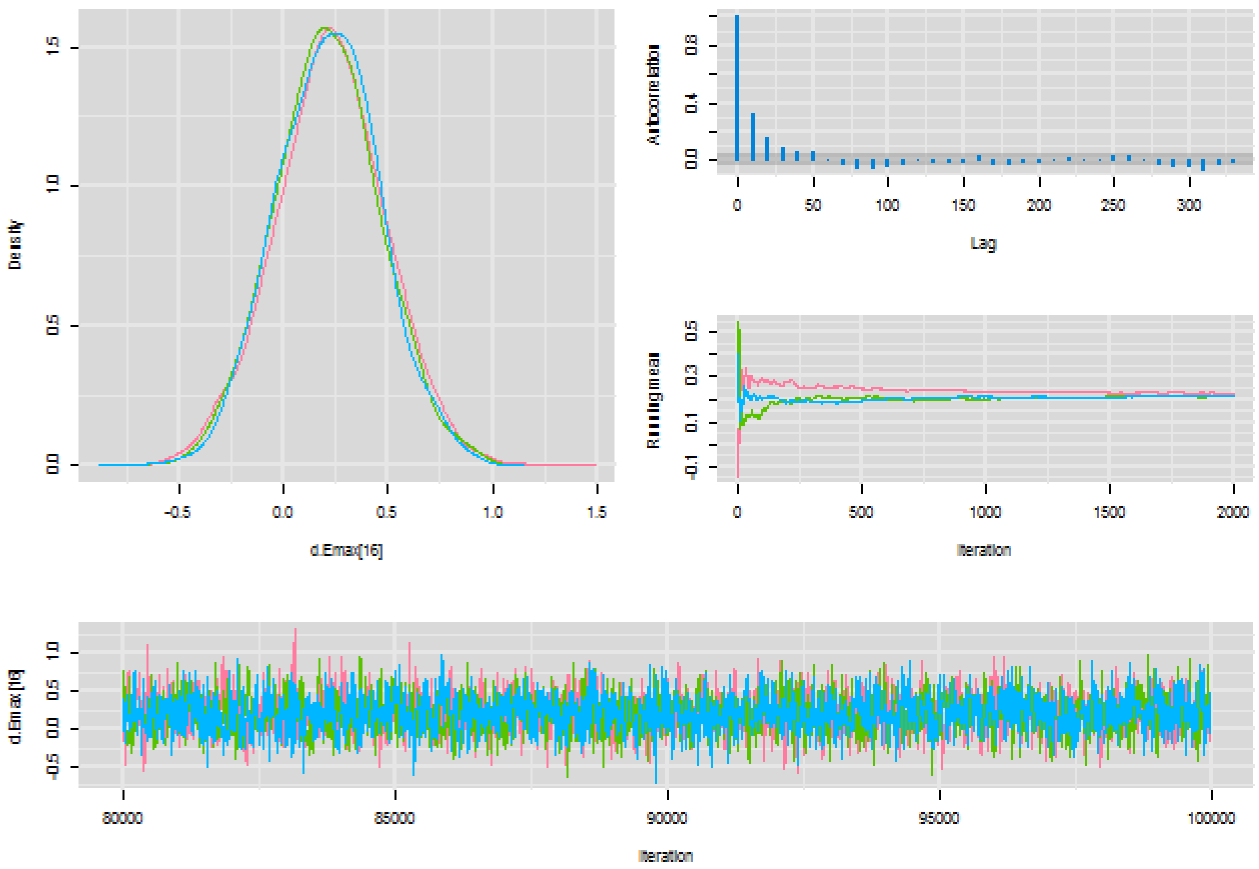

Diagnostics for d.Emax[17]

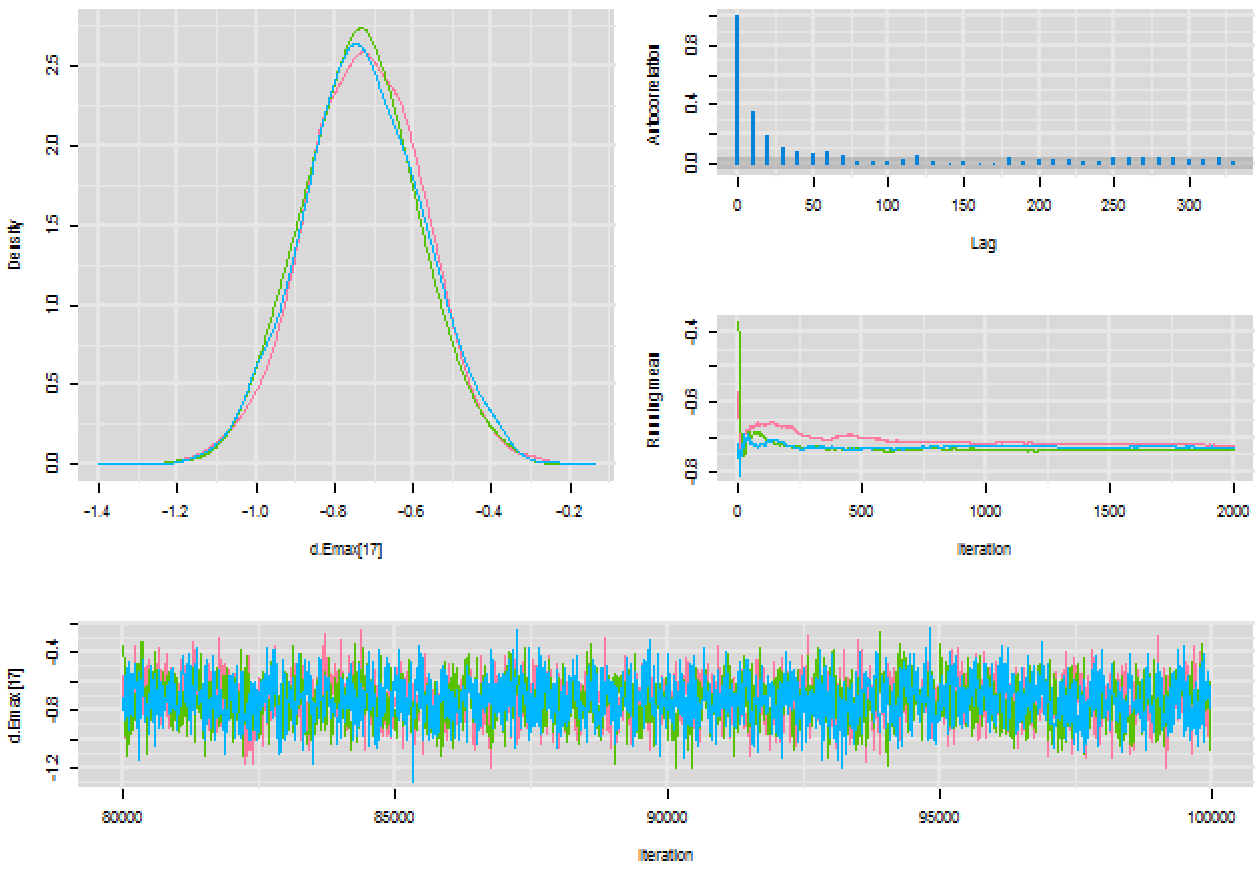

Diagnostics for d.Emax[18]

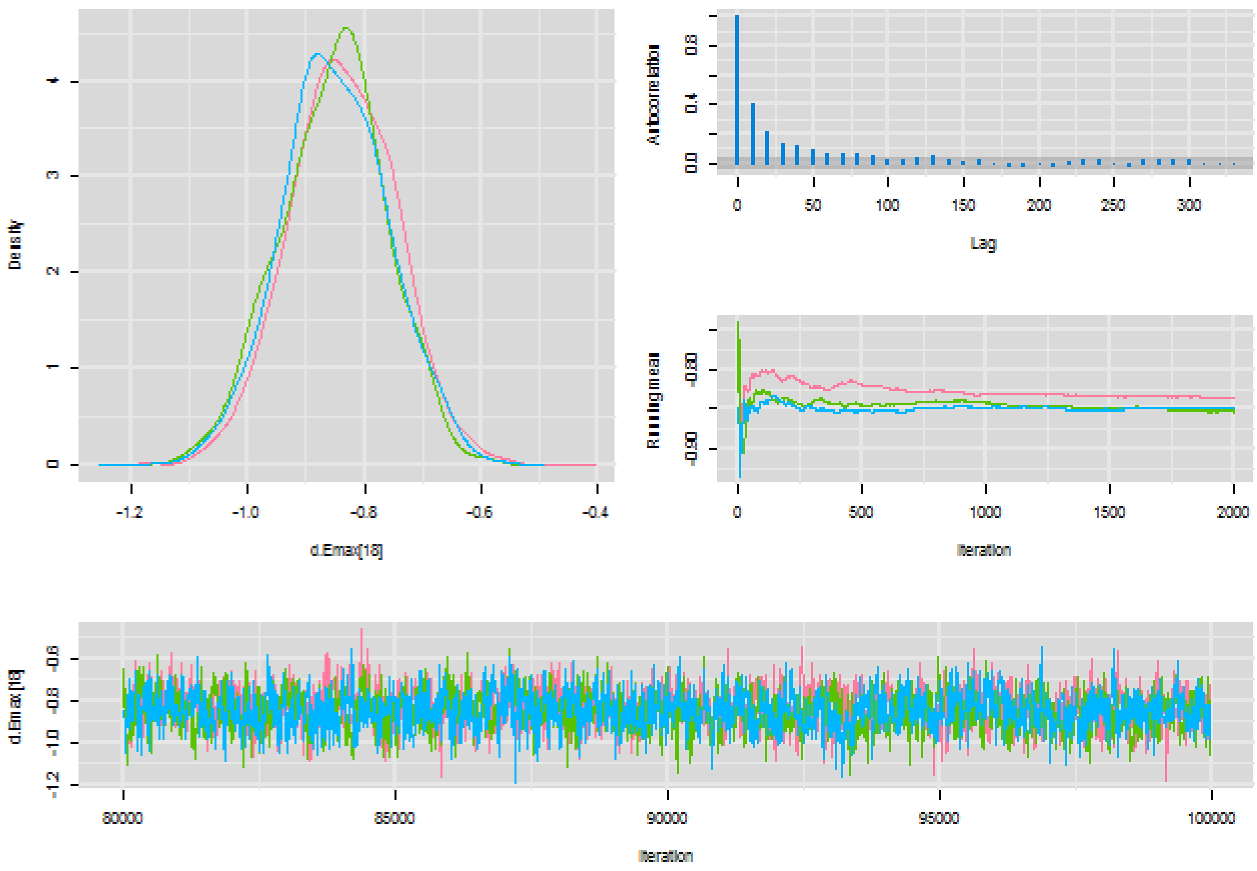

Diagnostics for d.Emax[19]

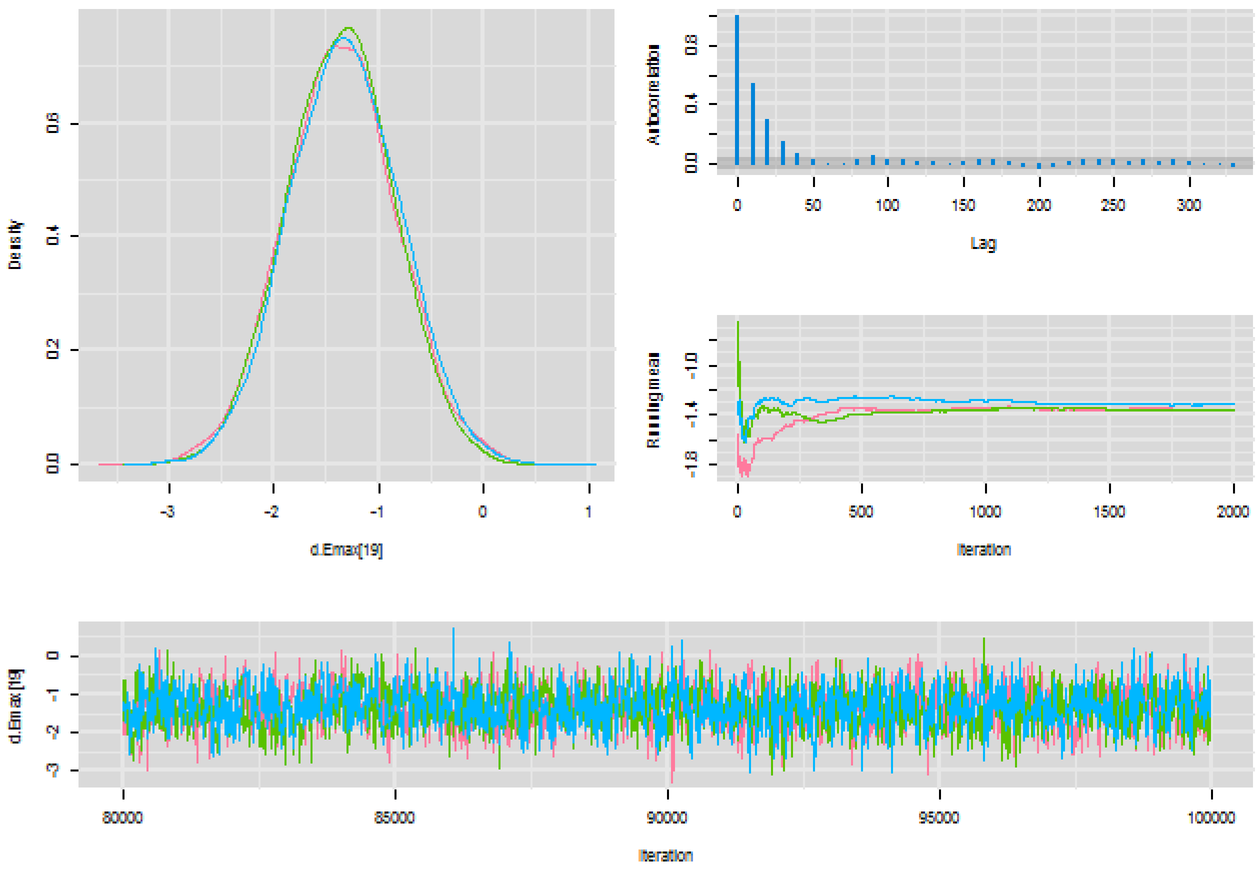

Diagnostics for d.Emax[20]

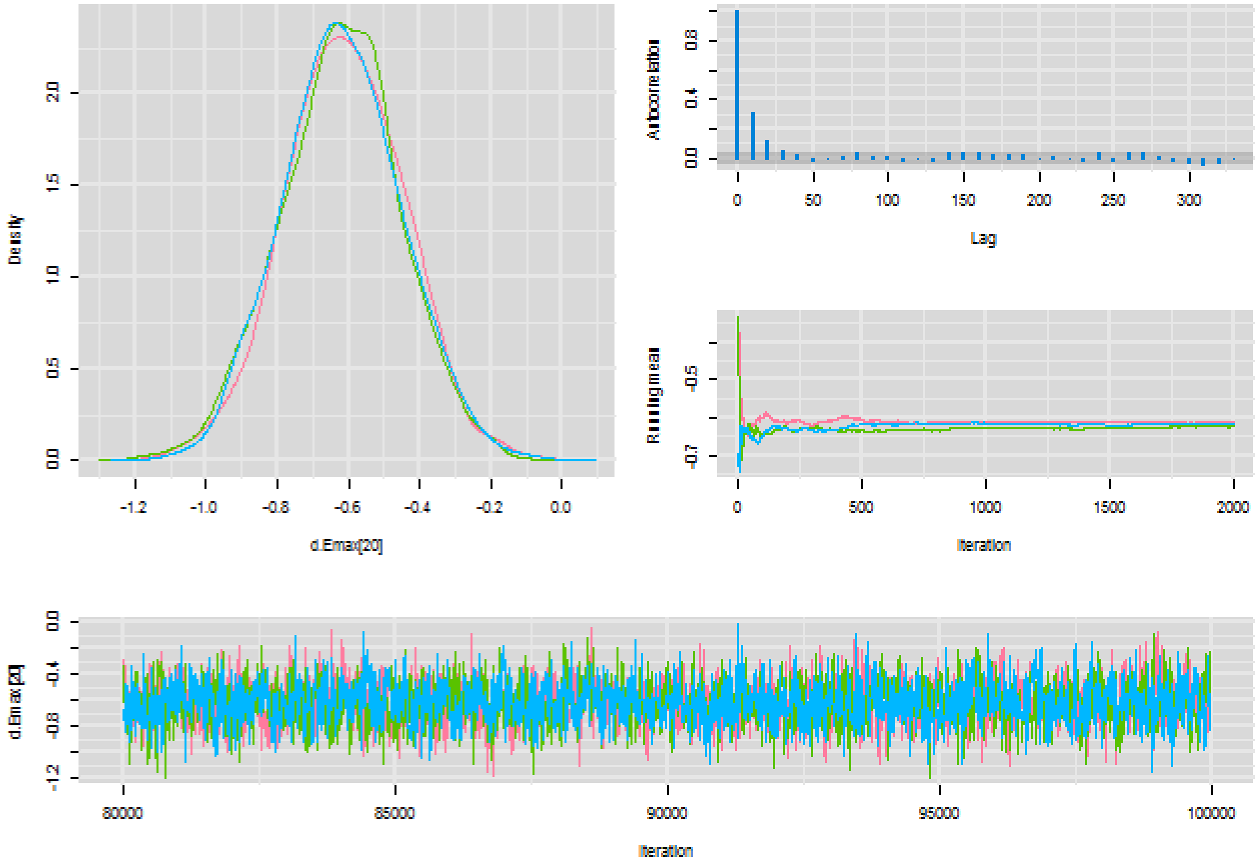

Diagnostics for d.Emax[21]

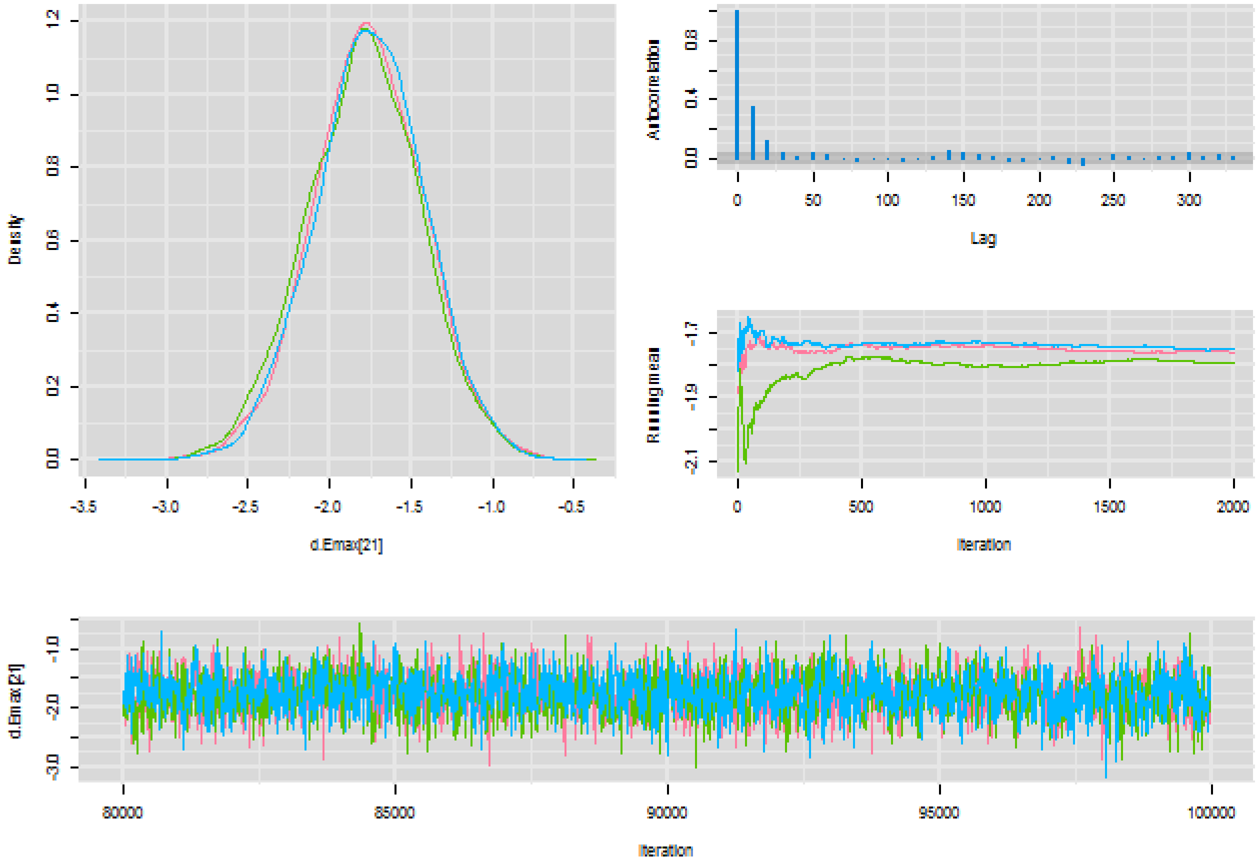

Diagnostics for d.Emax[22]

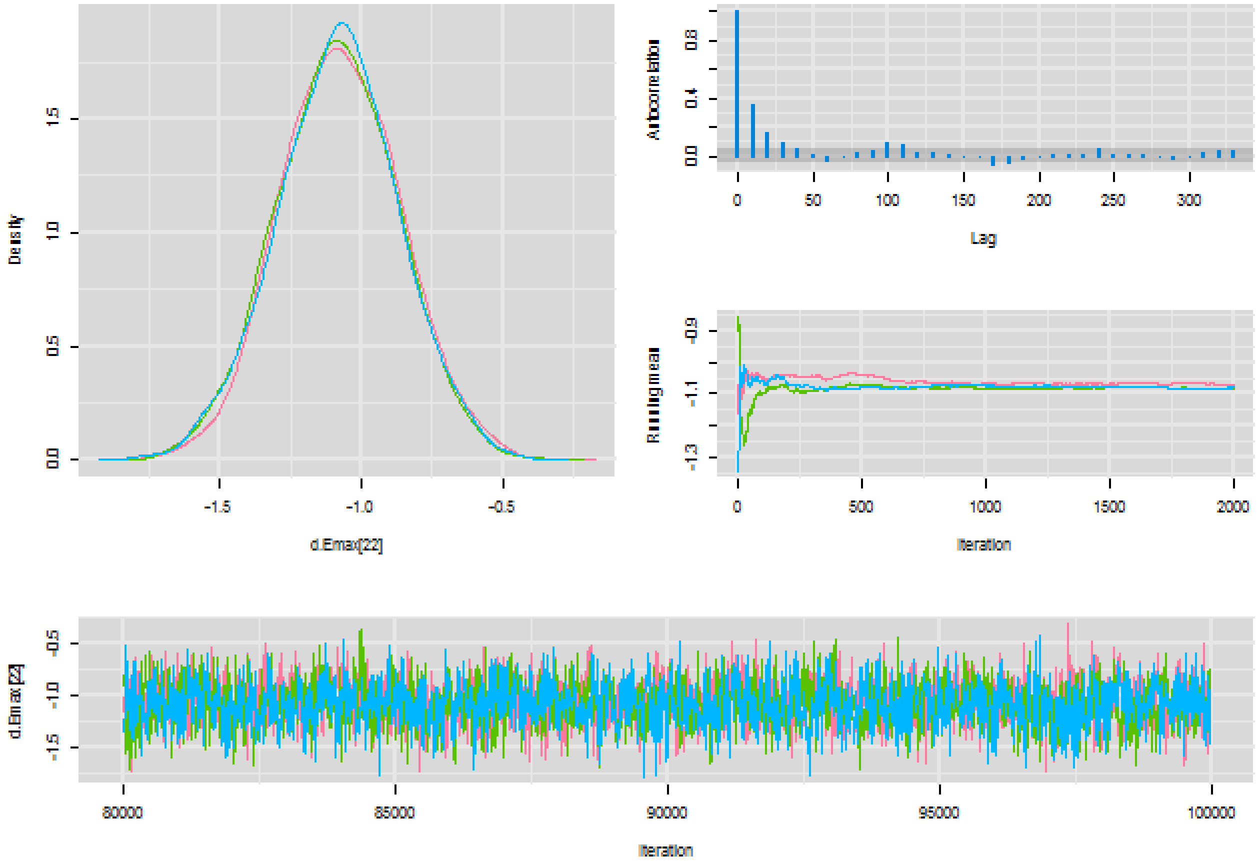

Diagnostics for d.Emax[23]

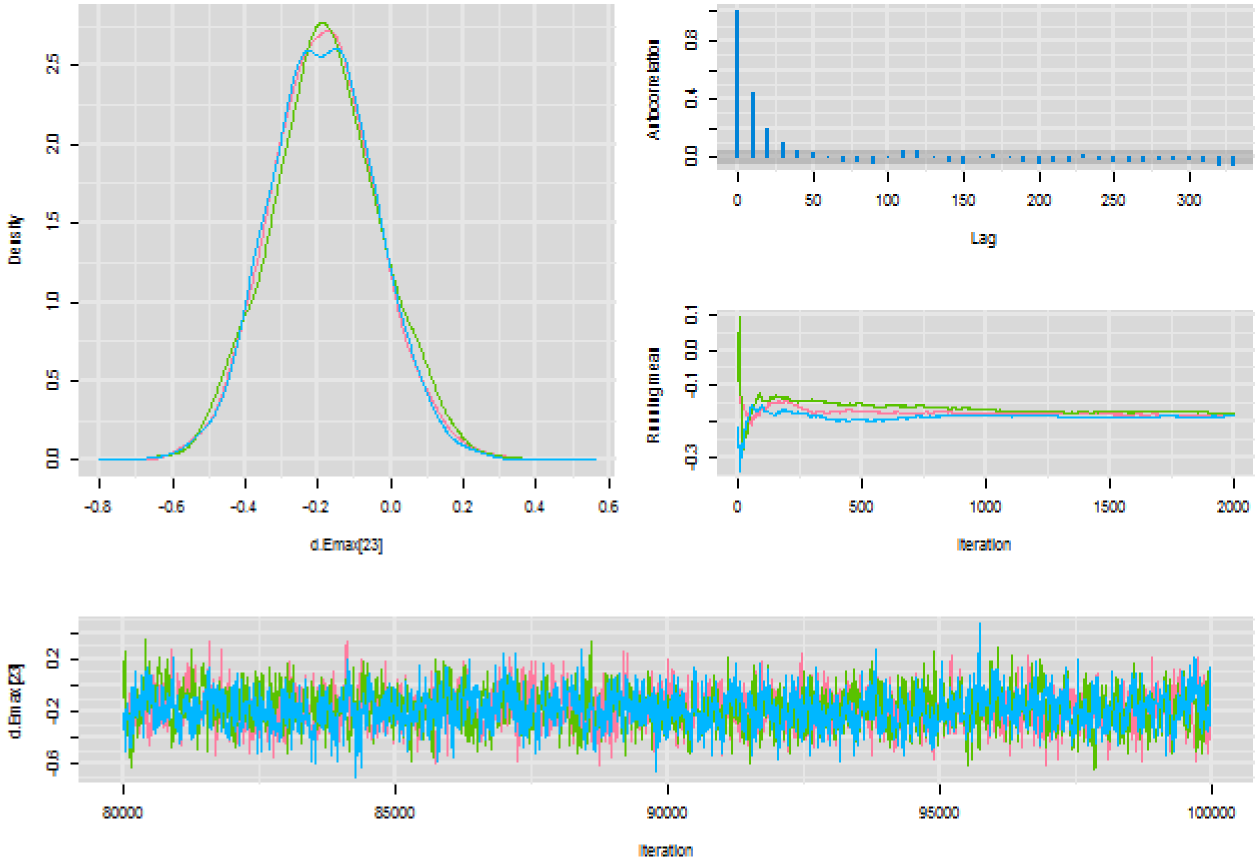

Diagnostics for d.Emax[24]

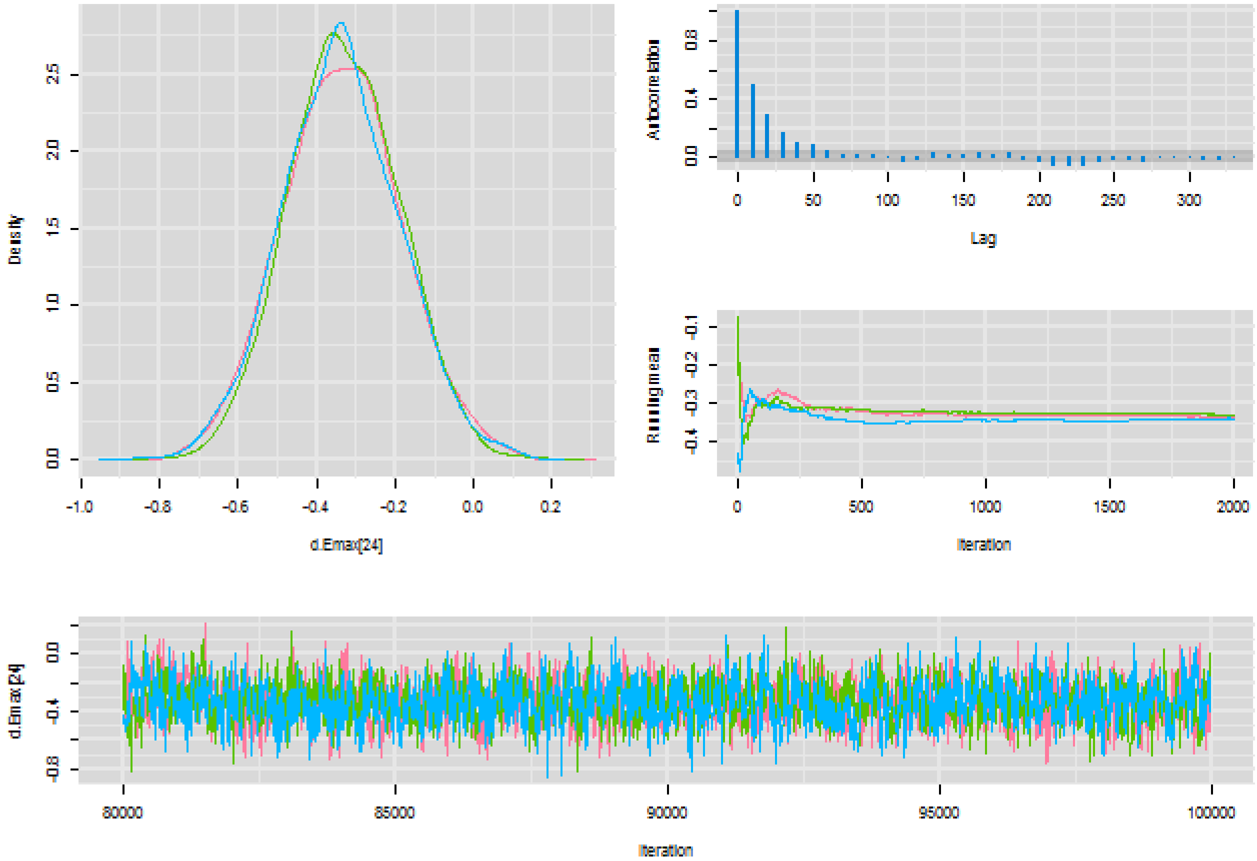

Diagnostics for d.Emax[25]

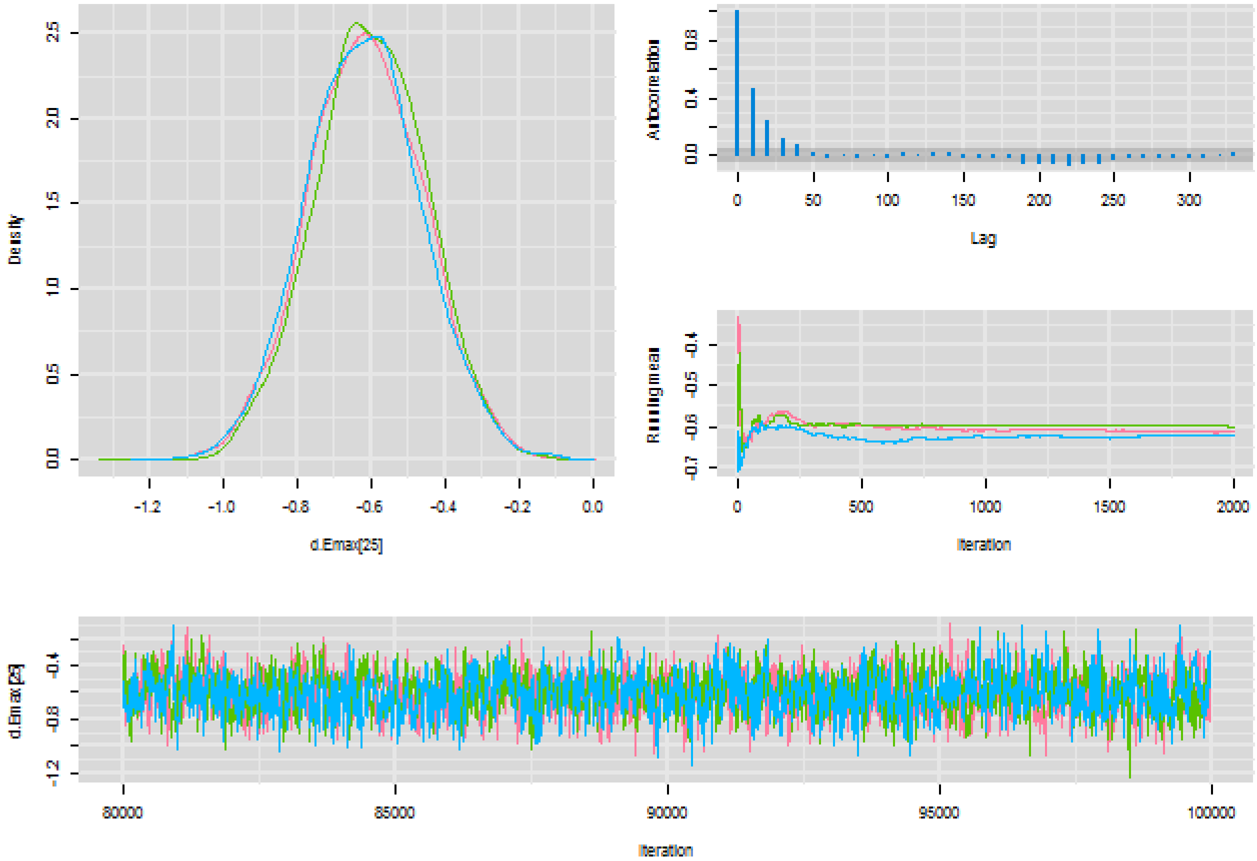

Diagnostics for d.Emax[26]

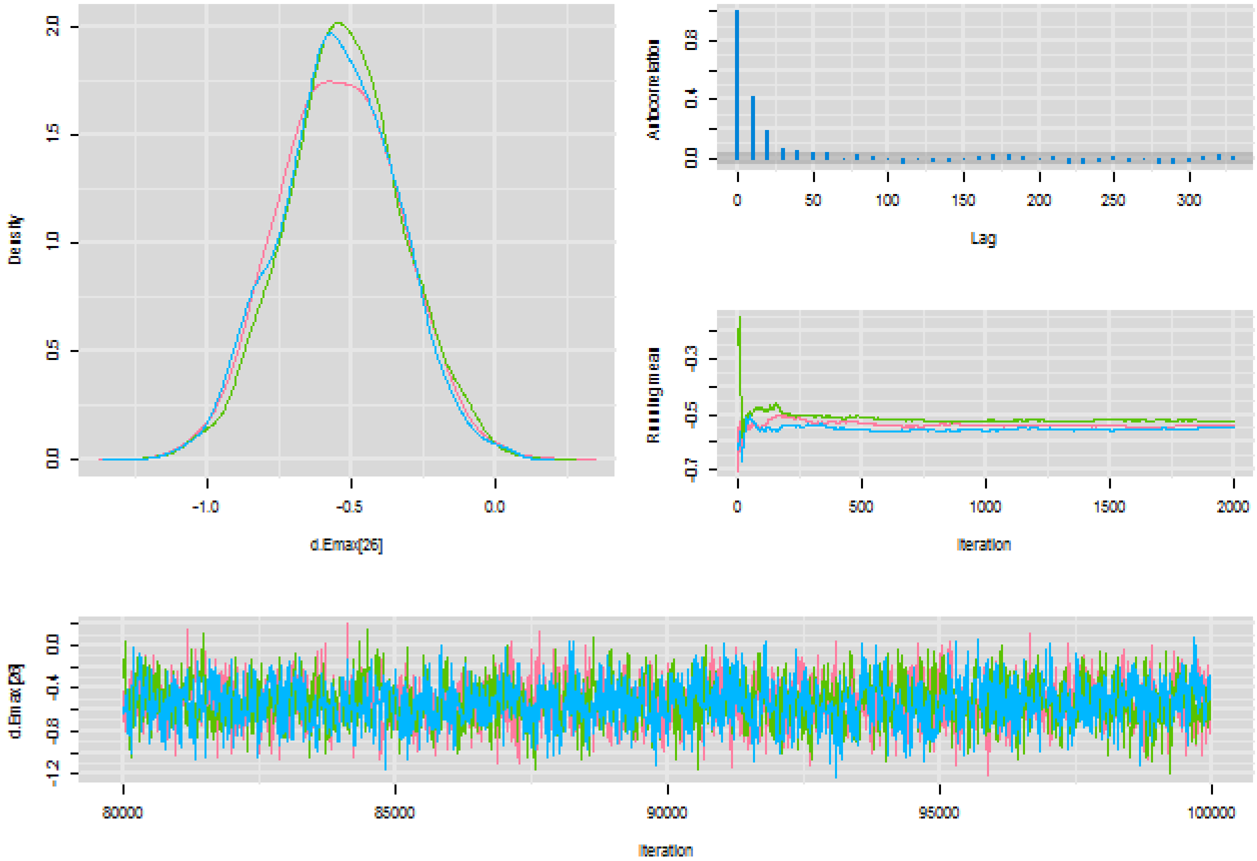

Diagnostics for d.Emax[27]

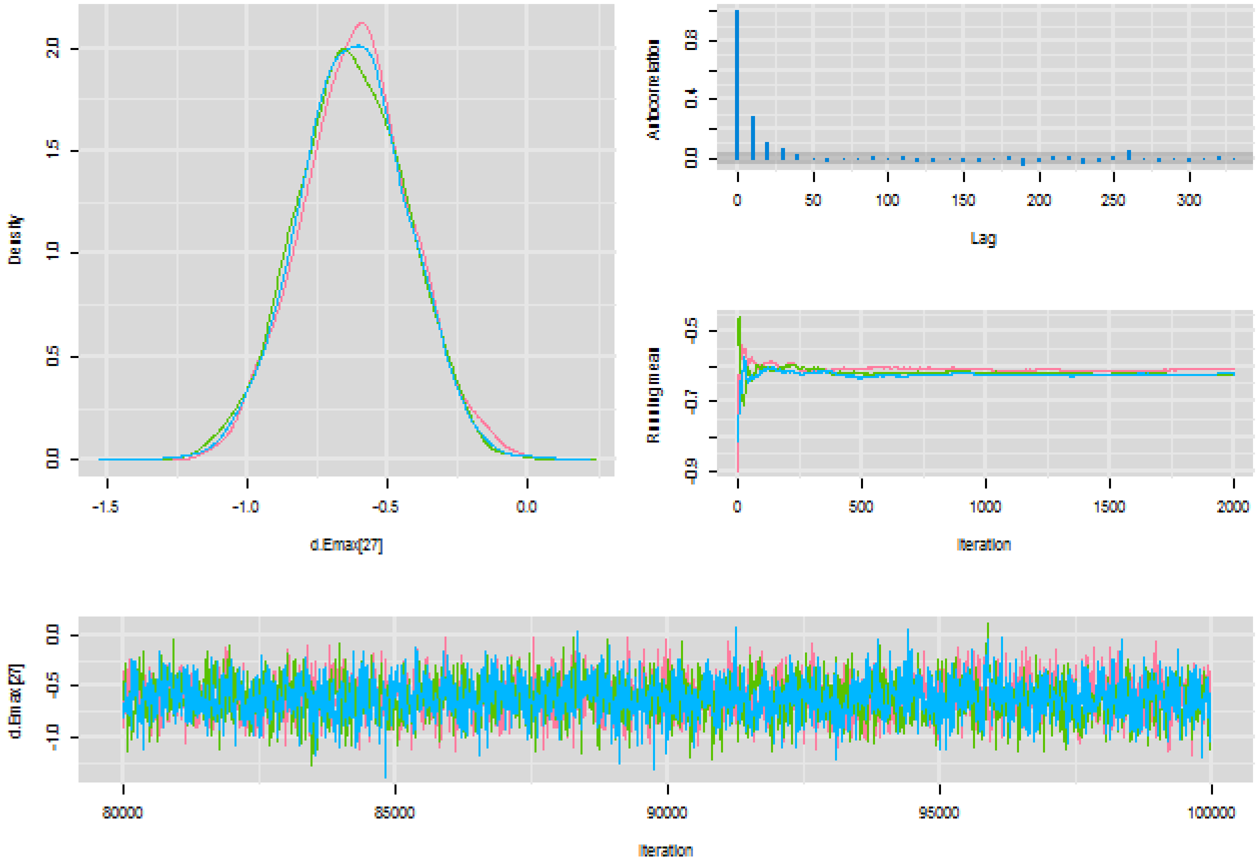

Diagnostics for d.Emax[28]

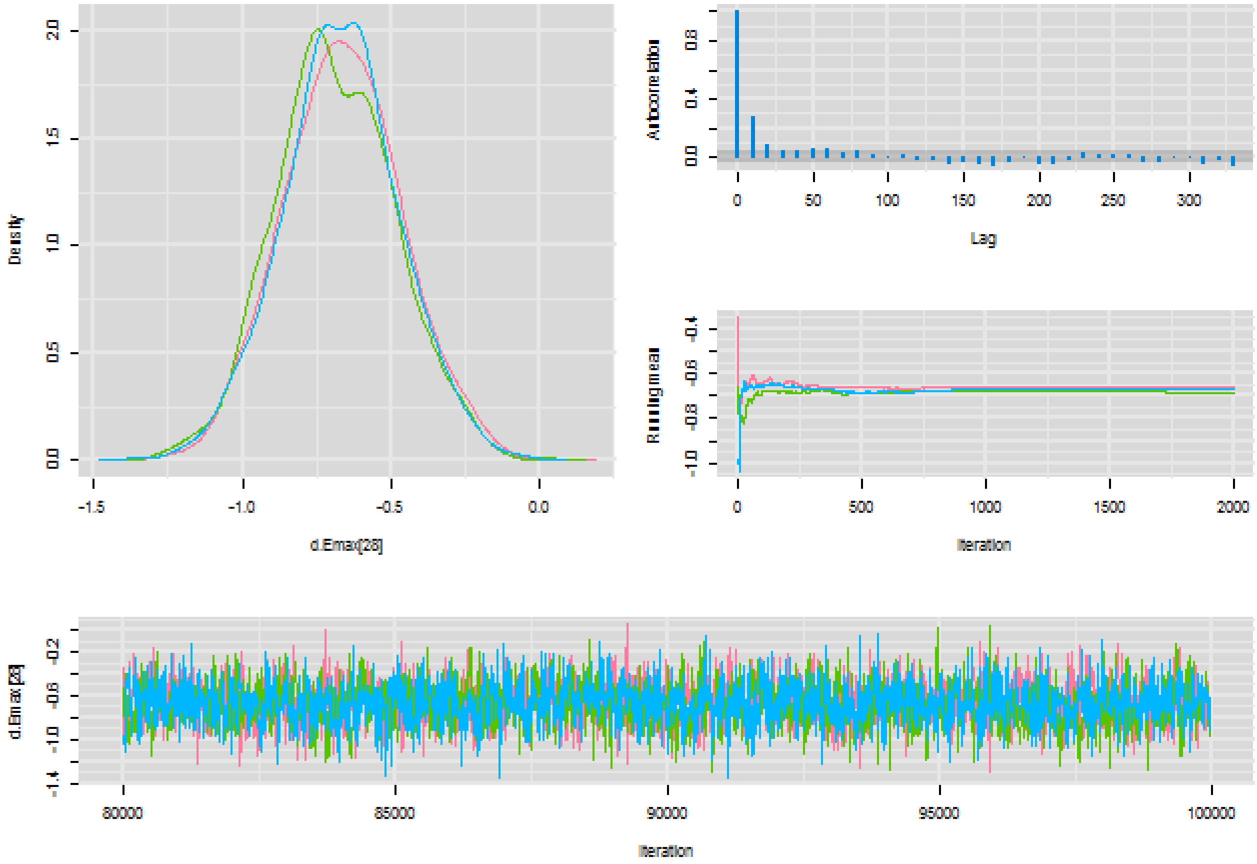

Diagnostics for d.Emax[29]

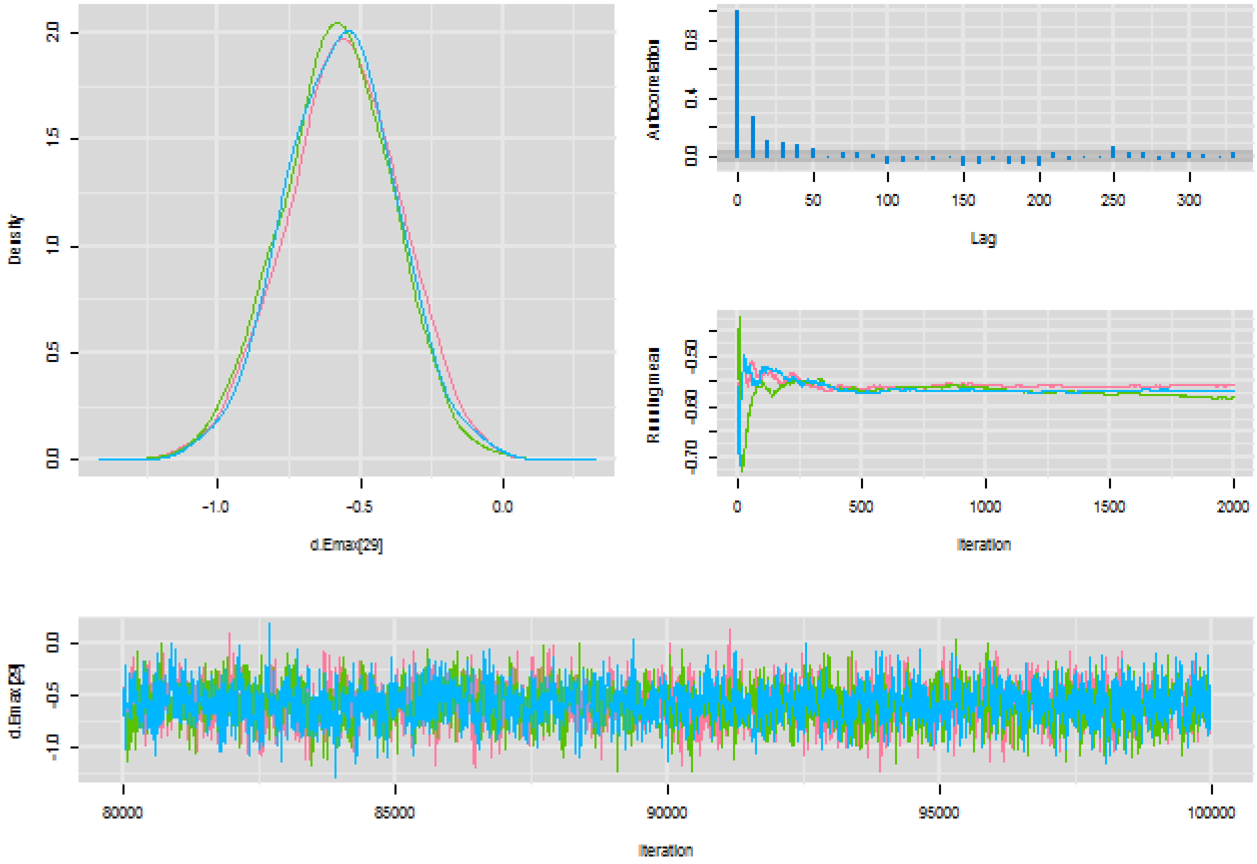

Plots for d.ET50

d.ET50[1]. Error in bw.SJ(x, method = "ste"): sample is too sparse to find TD

Diagnostics for d.ET50[2]

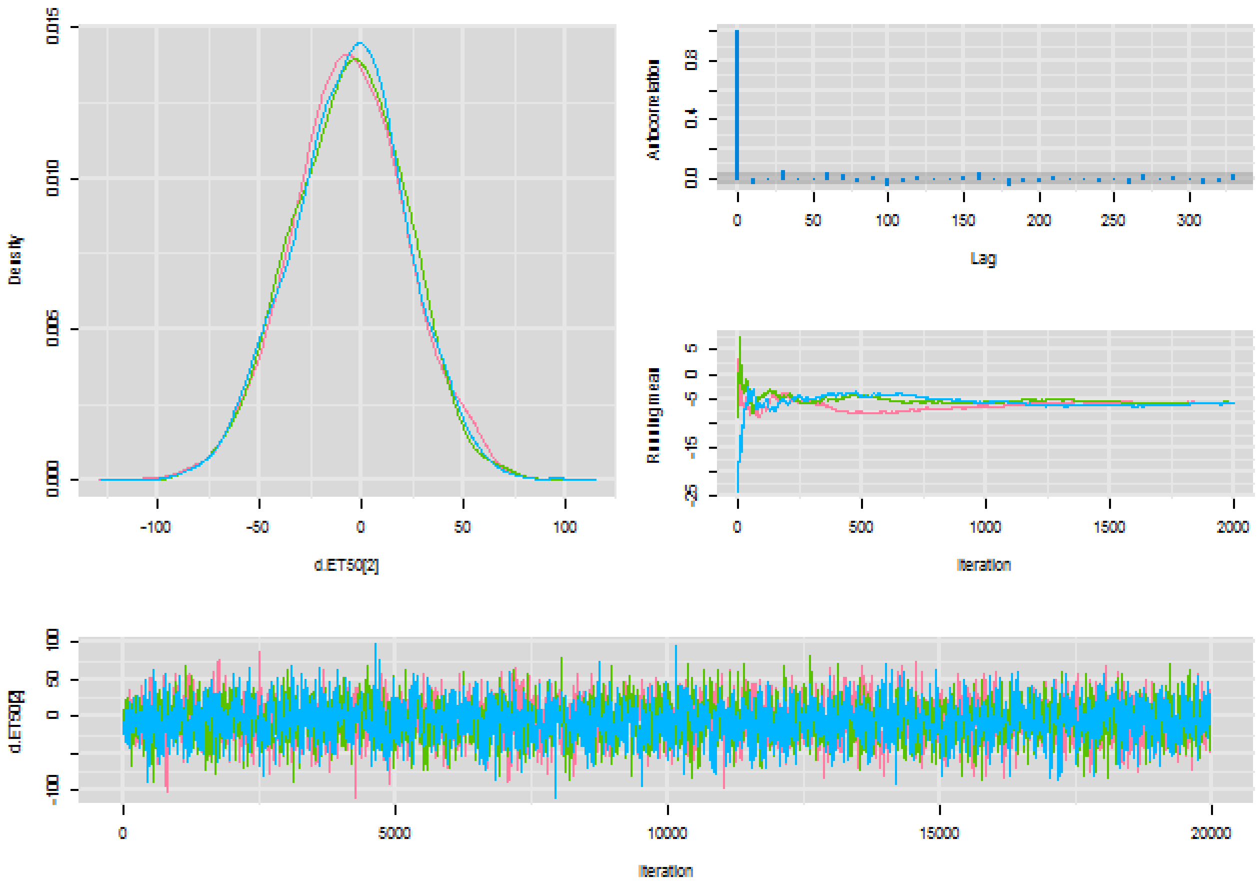

Diagnostics for d.ET50[3]

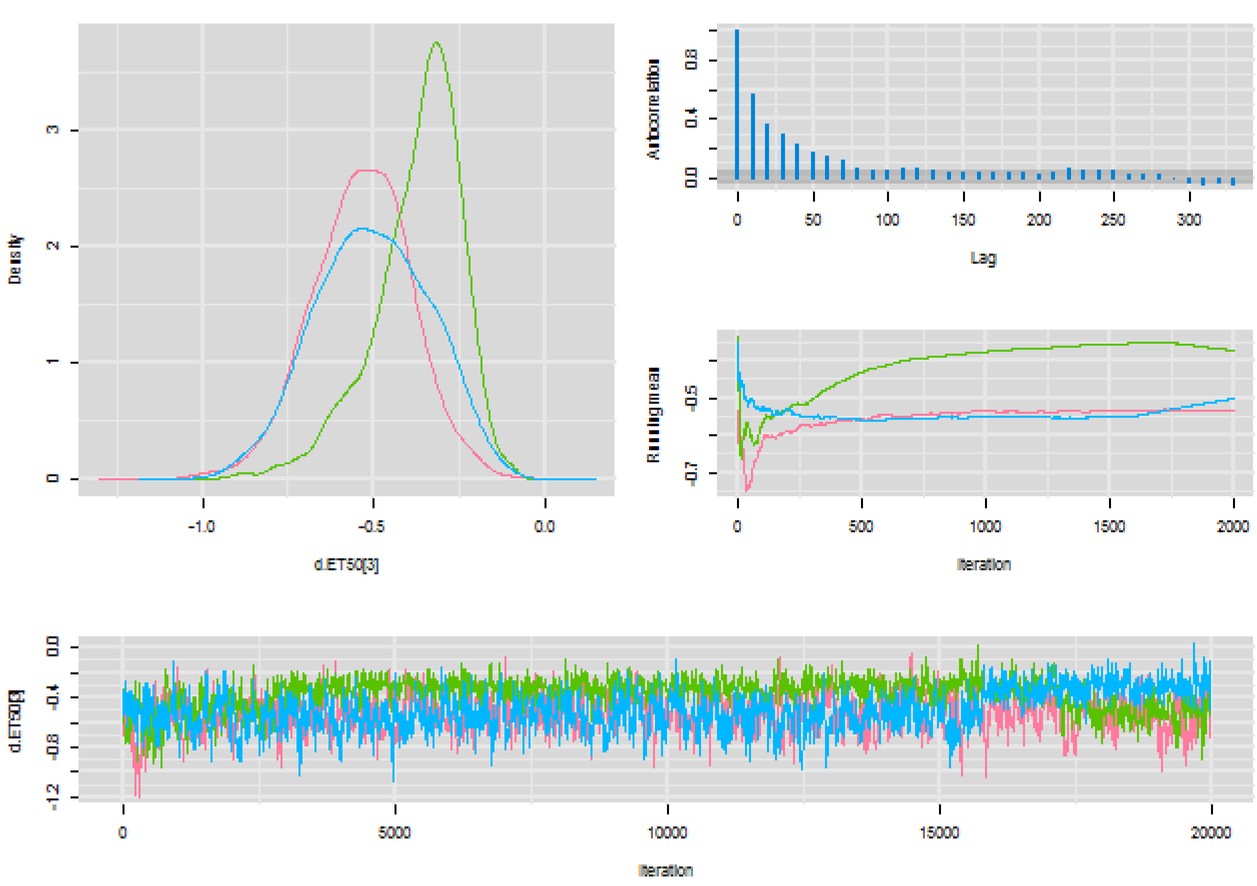

Diagnostics for d.ET50[4]

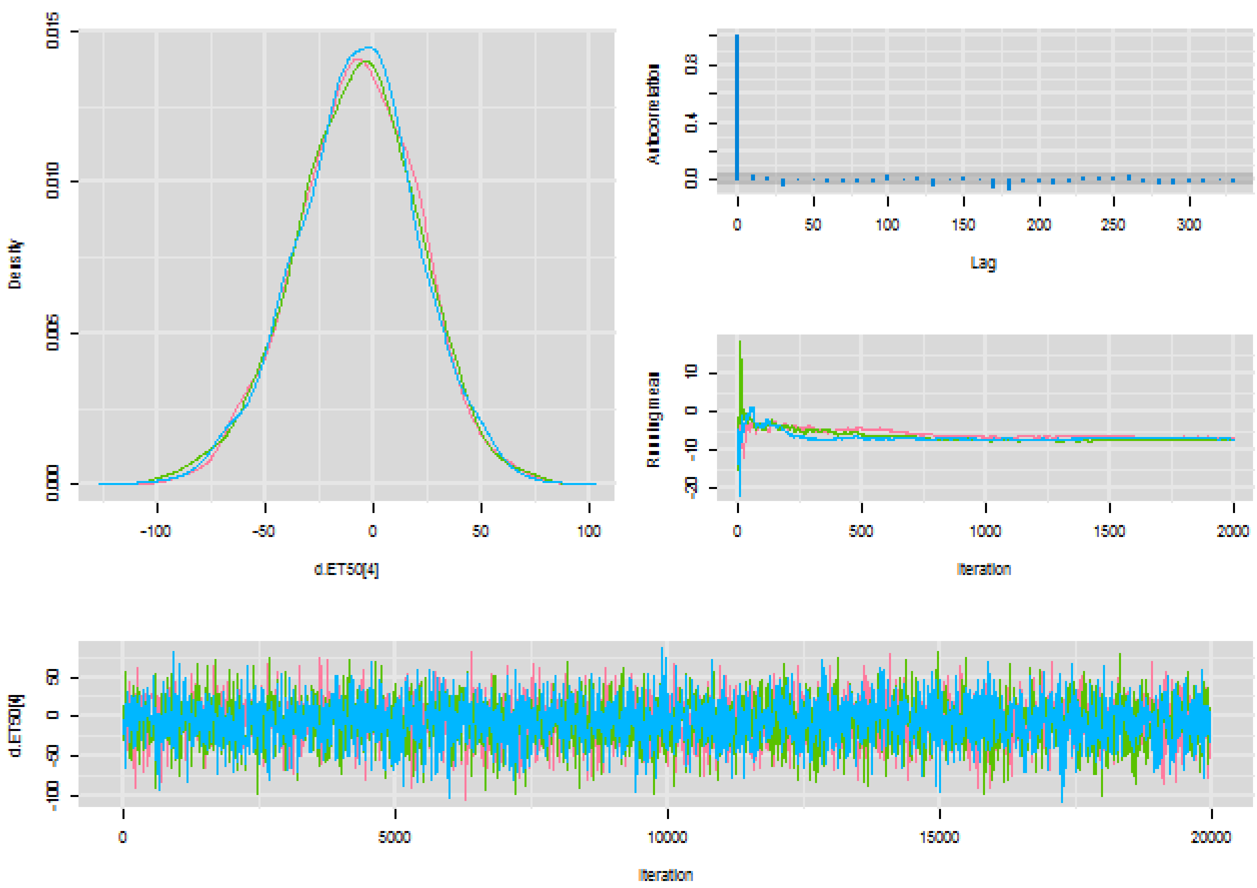

Diagnostics for d.ET50[5]

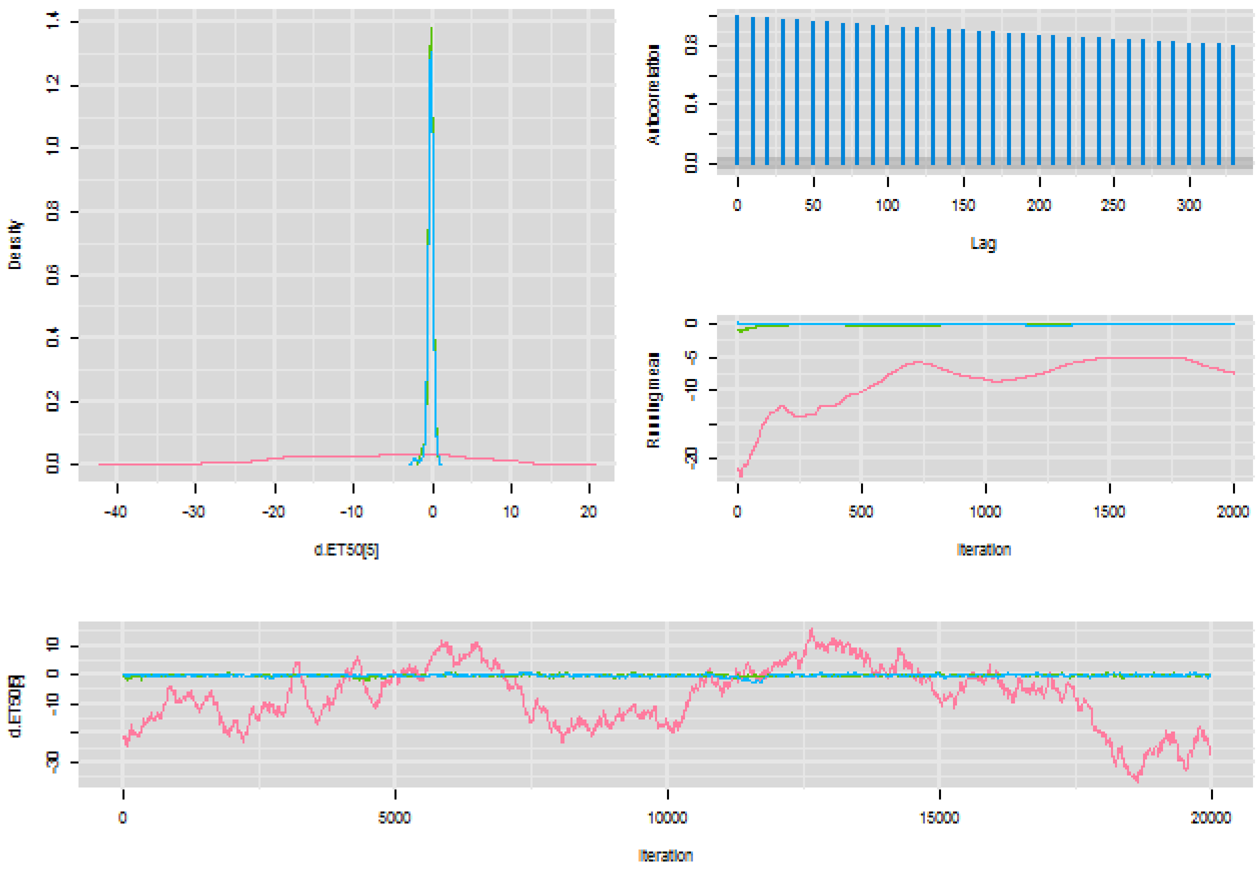

Diagnostics for d.ET50[6]

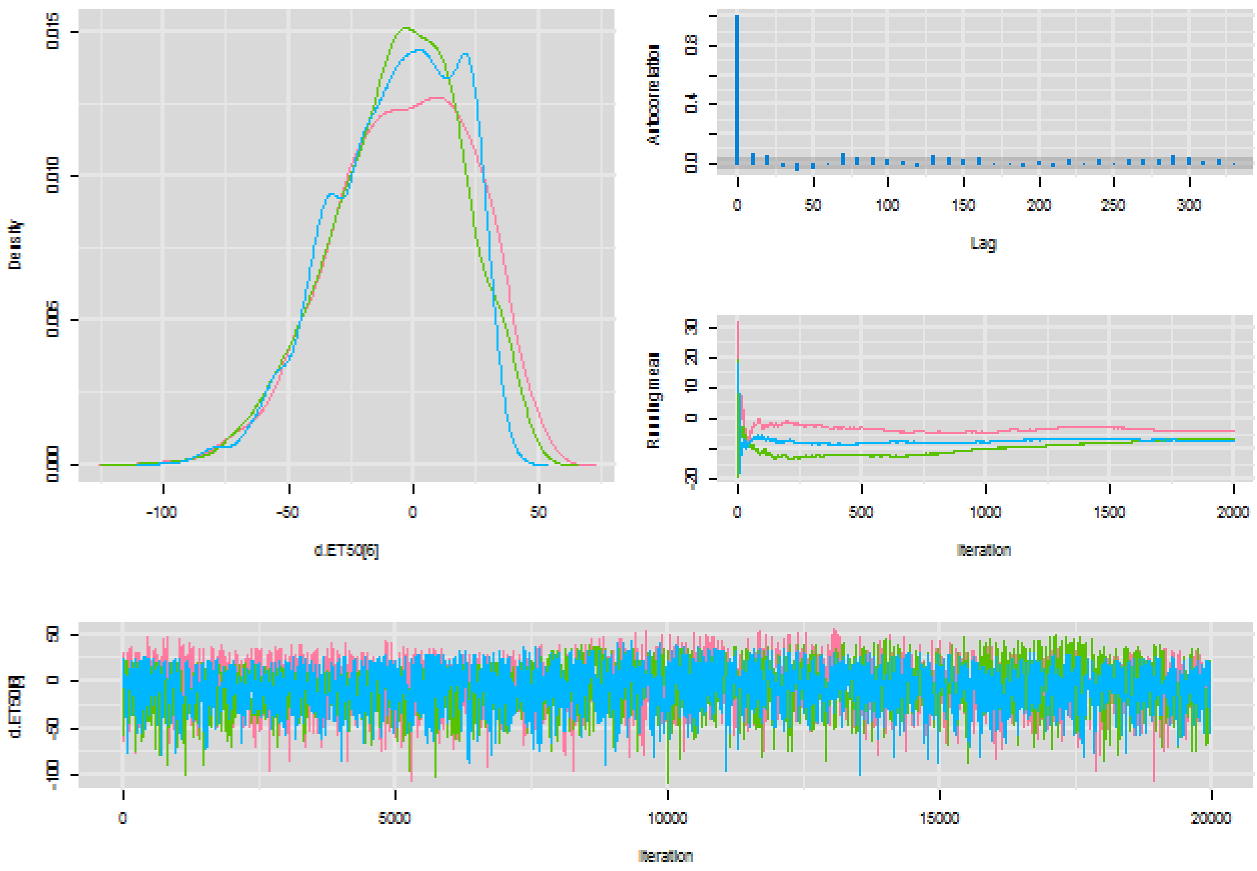

Diagnostics for d.ET50[7]

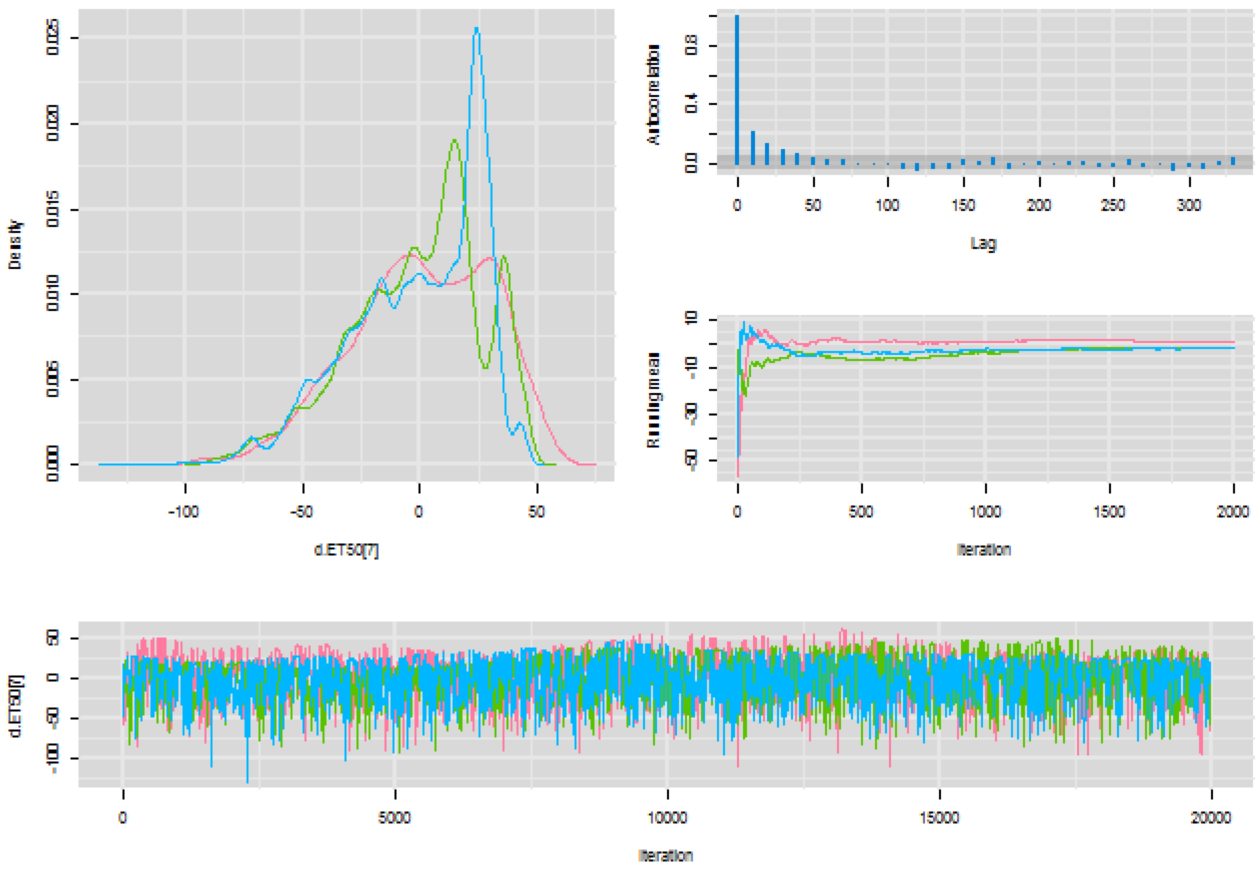

Diagnostics for d.ET50[8]

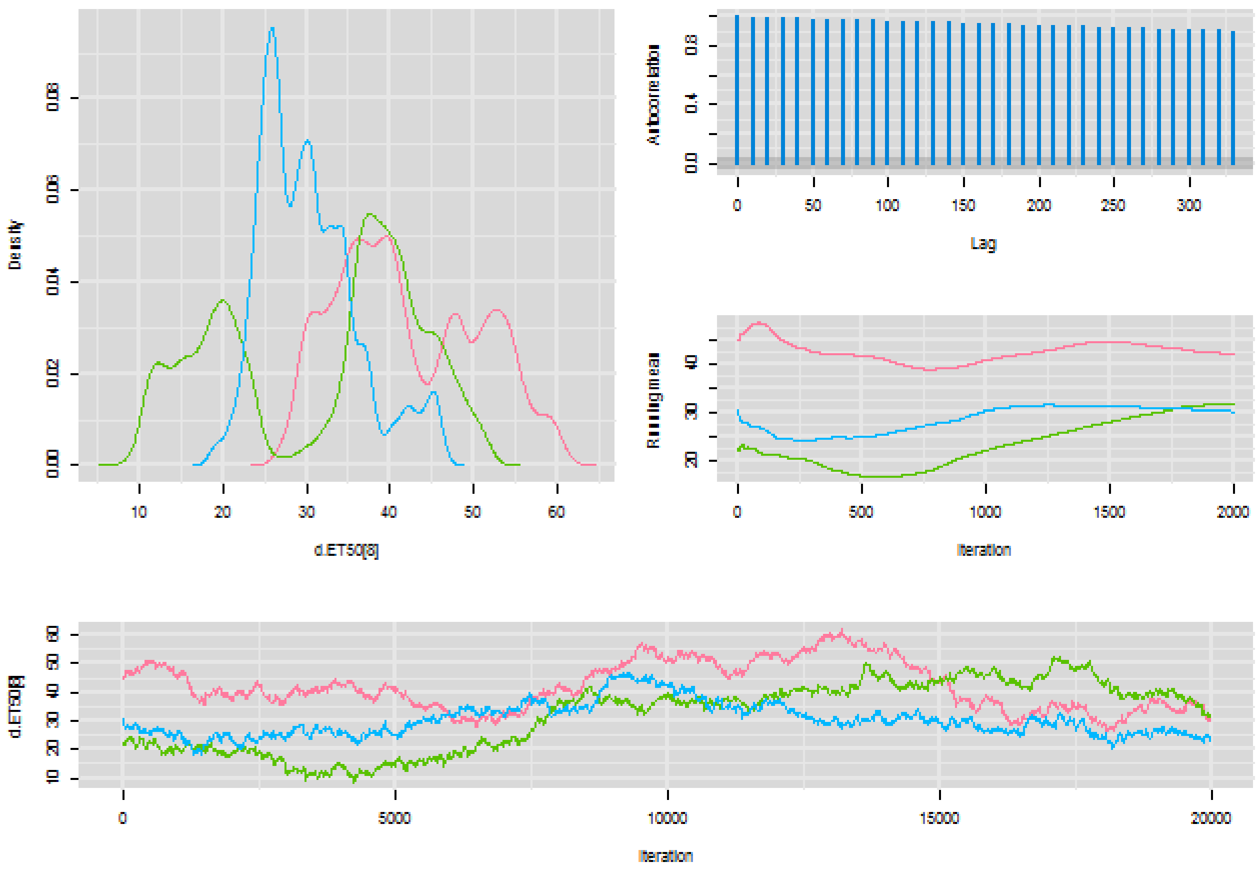

Diagnostics for d.ET50[9]

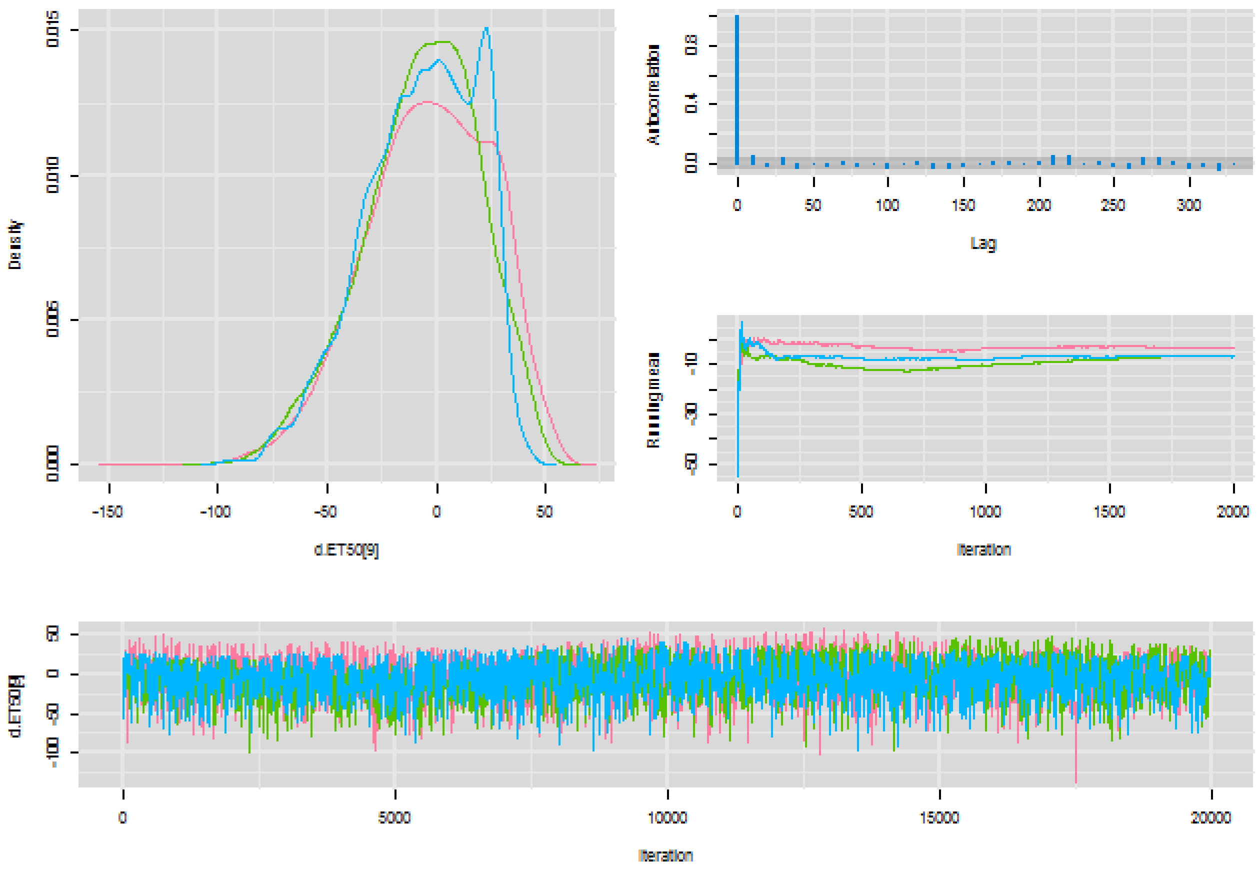

Diagnostics for d.ET50[10]

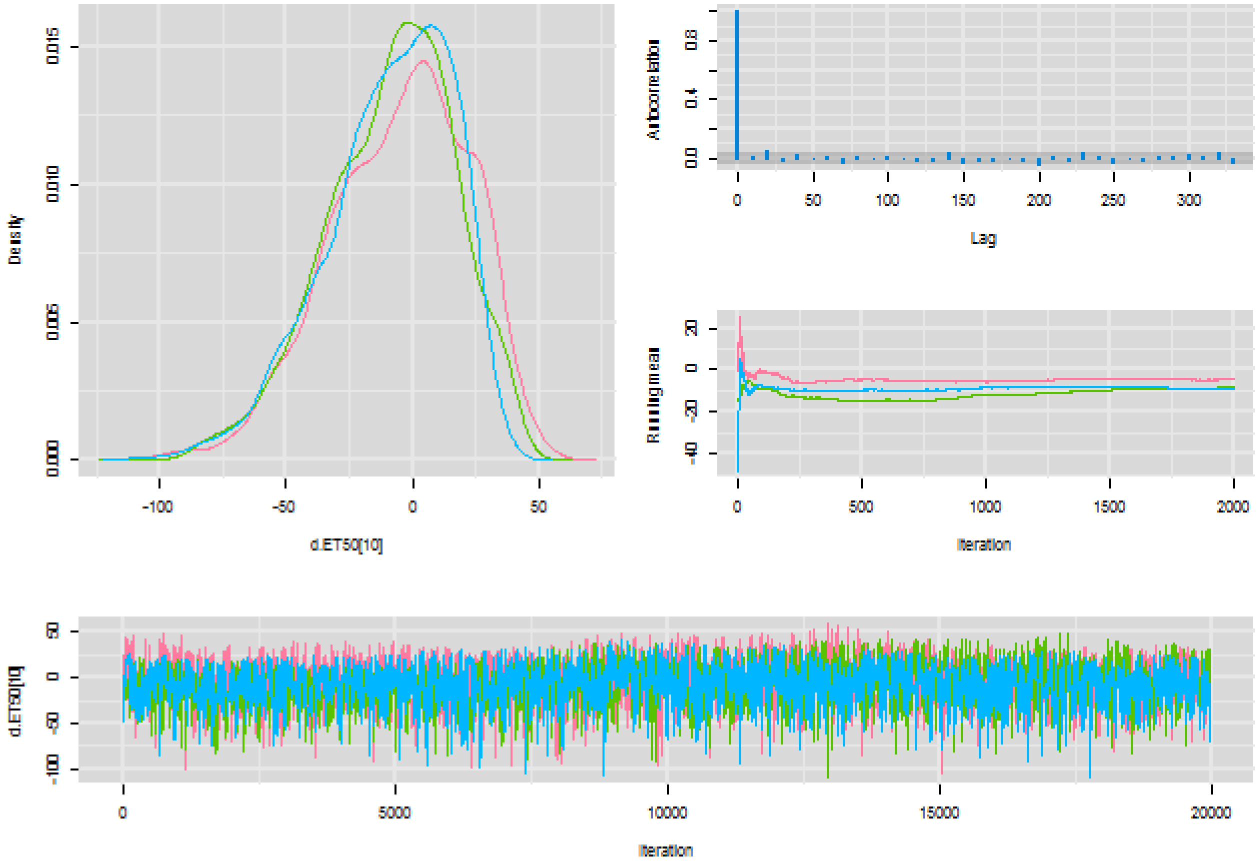

Diagnostics for d.ET50[11]

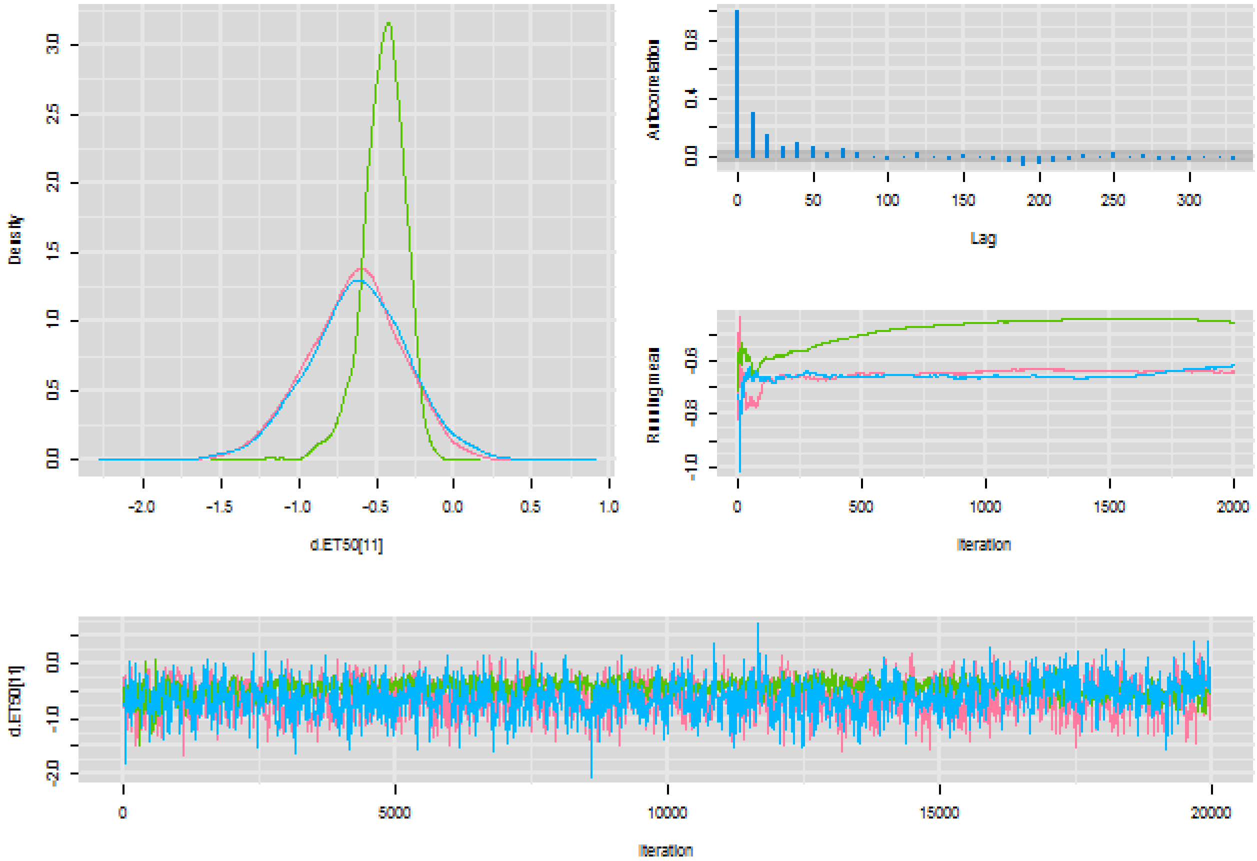

Diagnostics for d.ET50[12]

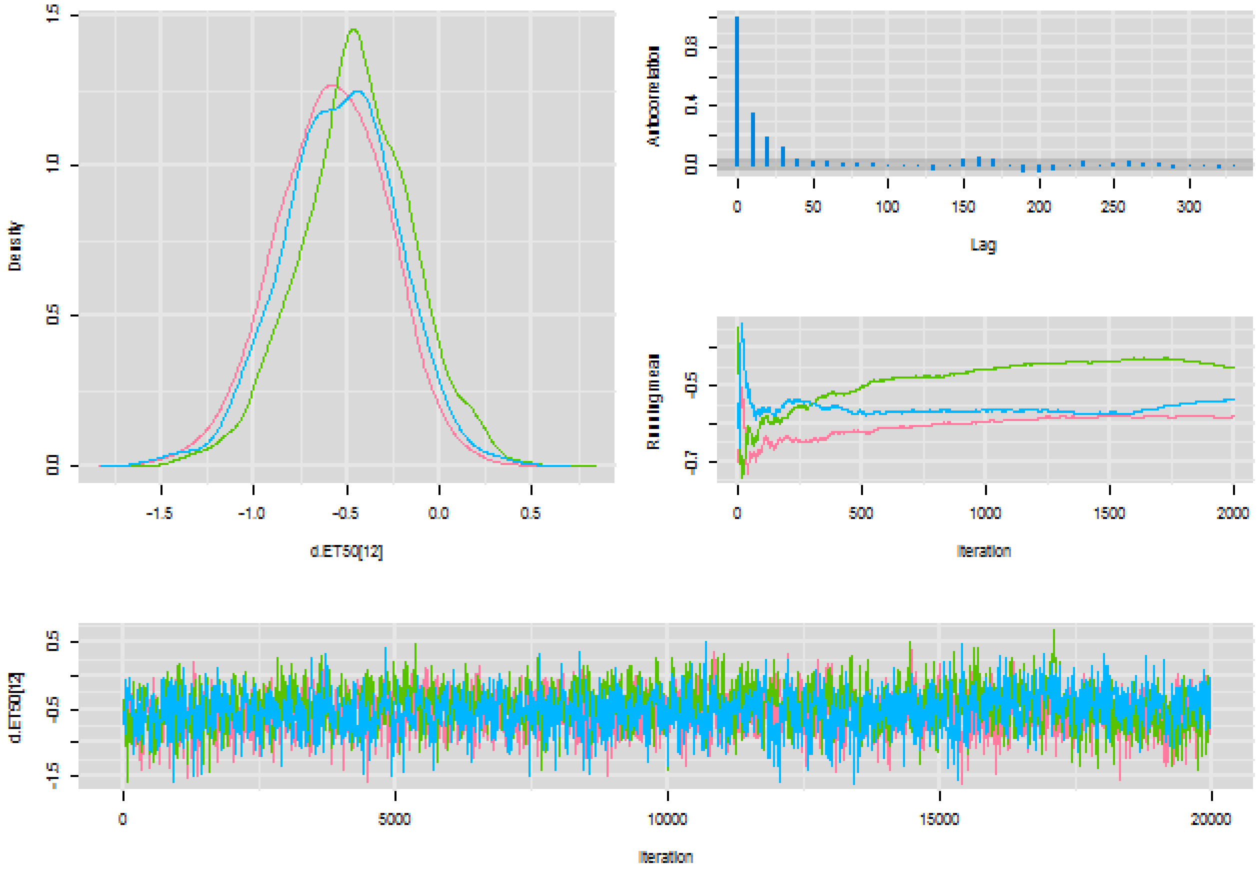

Diagnostics for d.ET50[13]

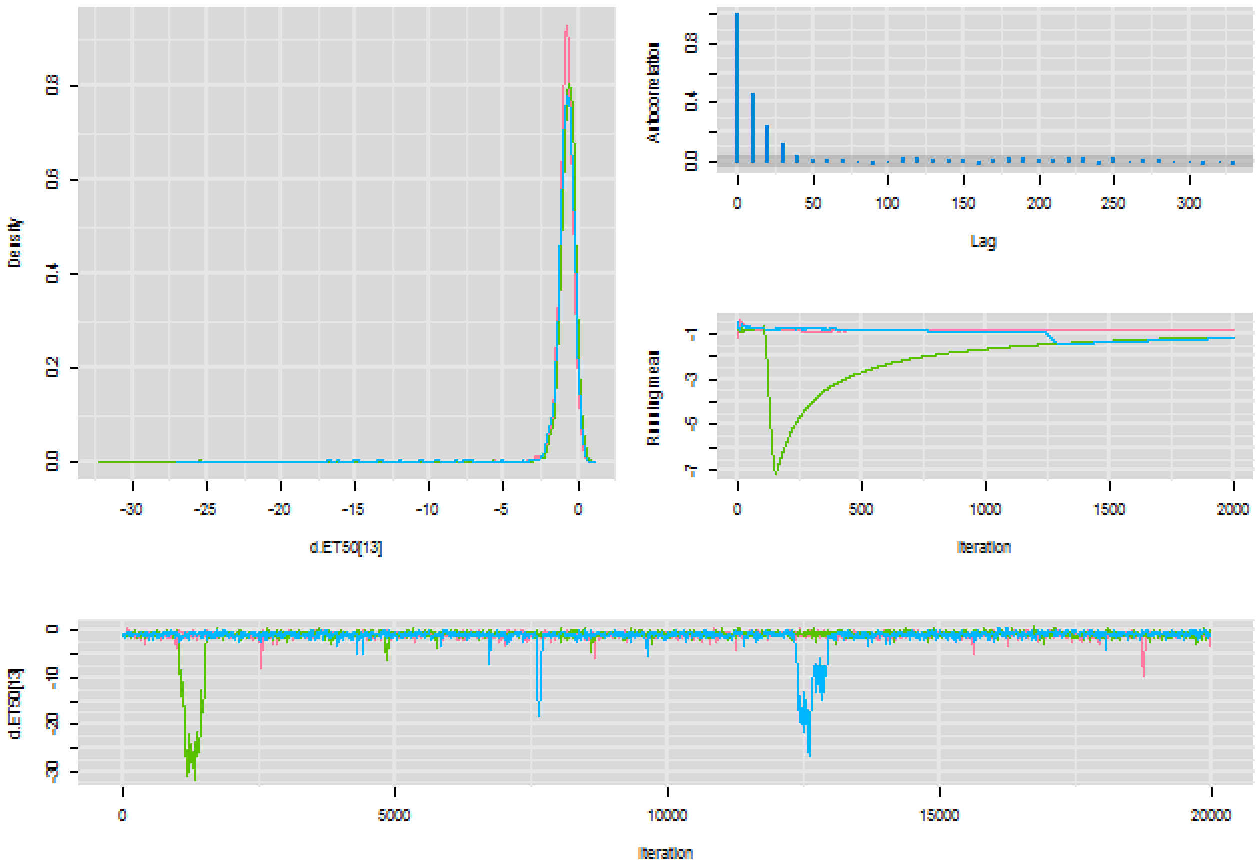

Diagnostics for d.ET50[14]

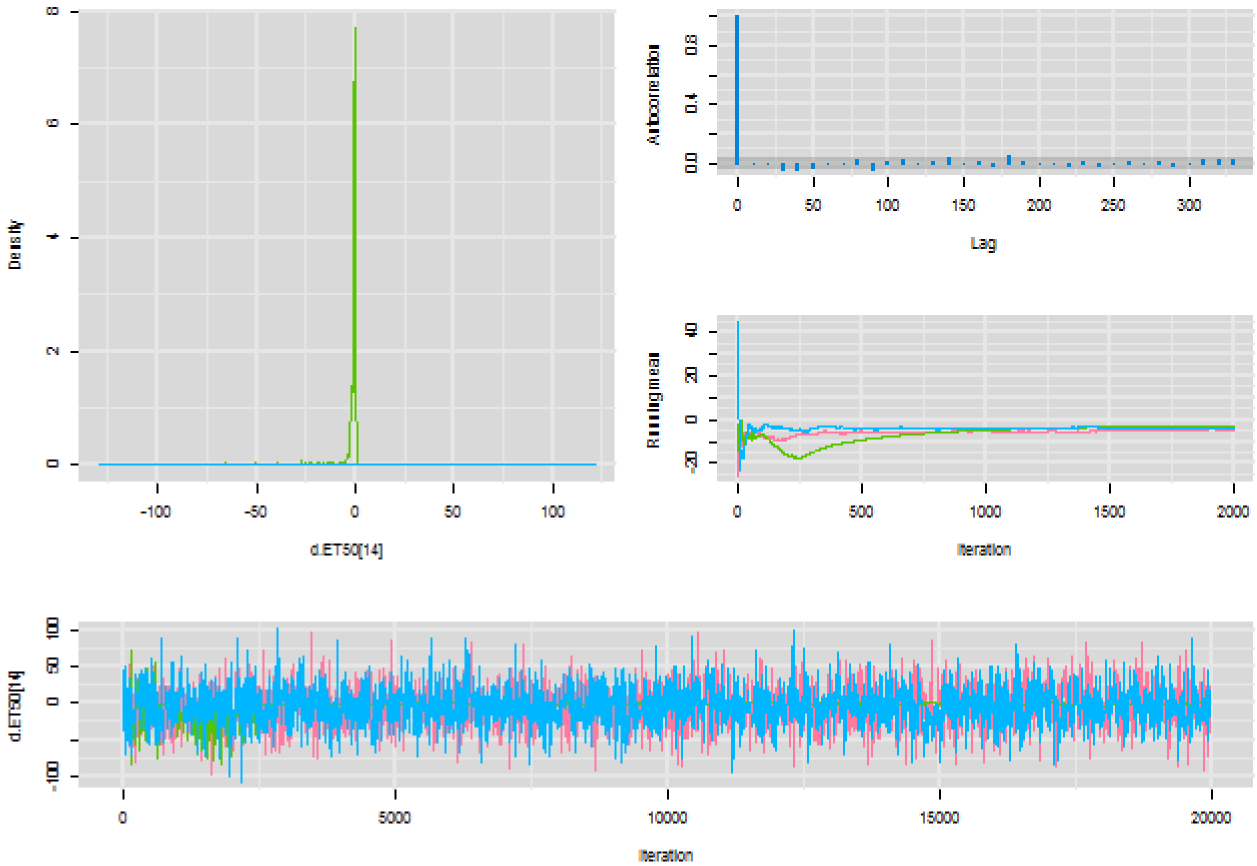

Diagnostics for d.ET50[15]

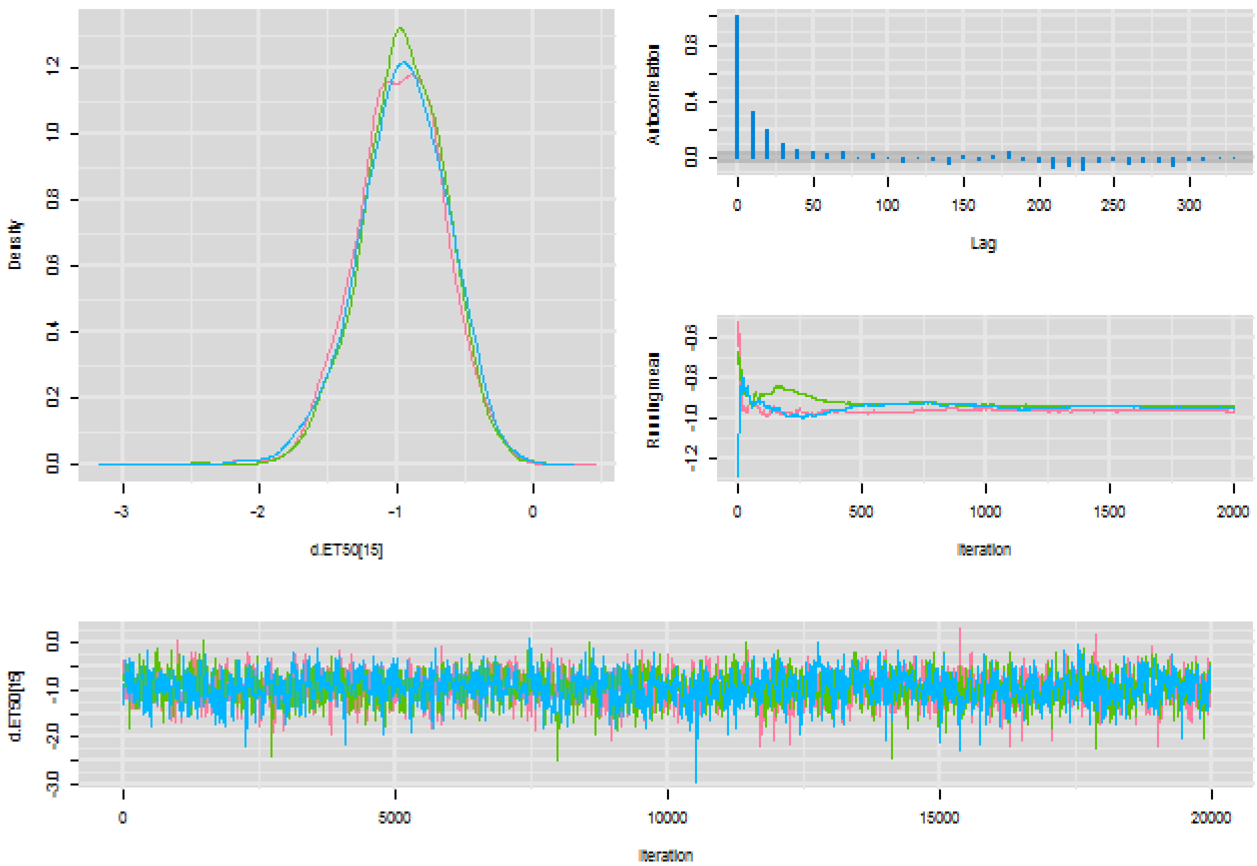

Diagnostics for d.ET50[16]

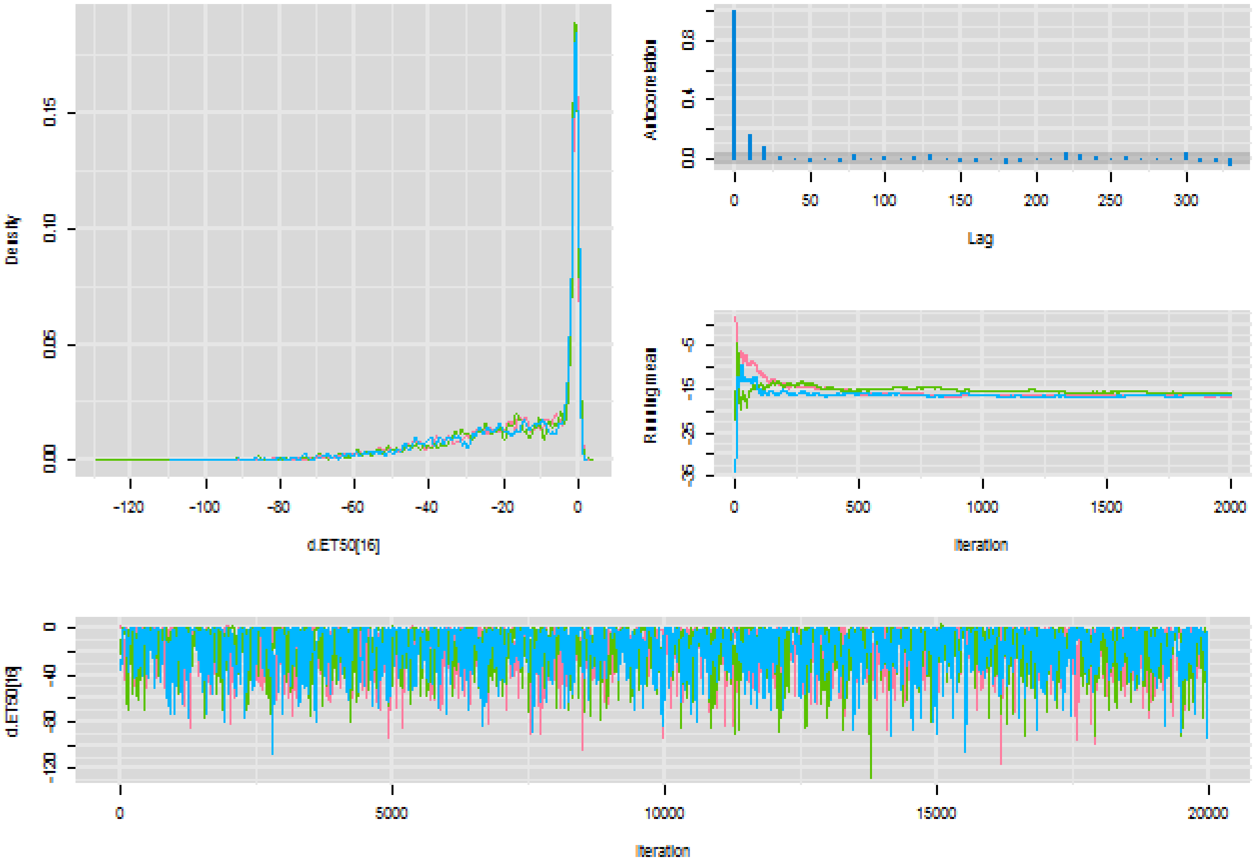

Diagnostics for d.ET50[17]

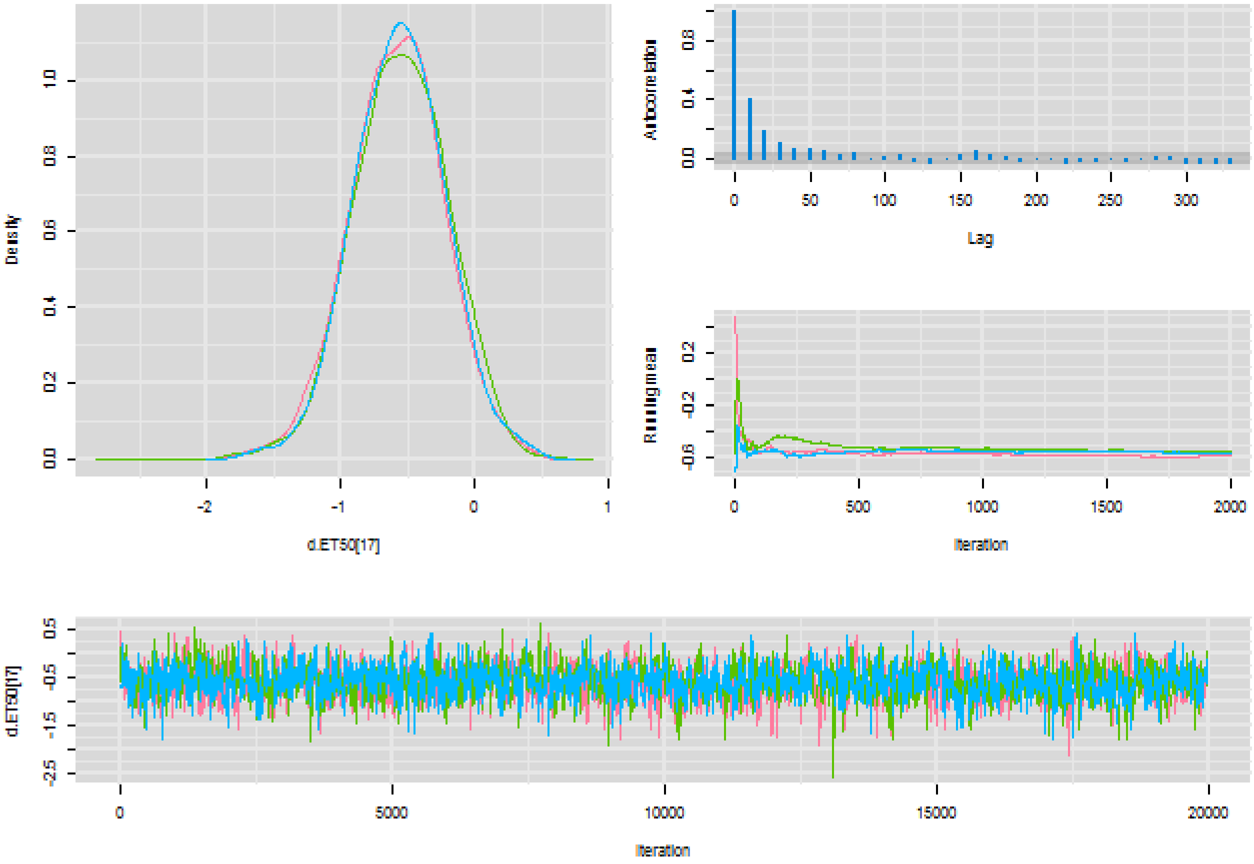

Diagnostics for d.ET50[18]

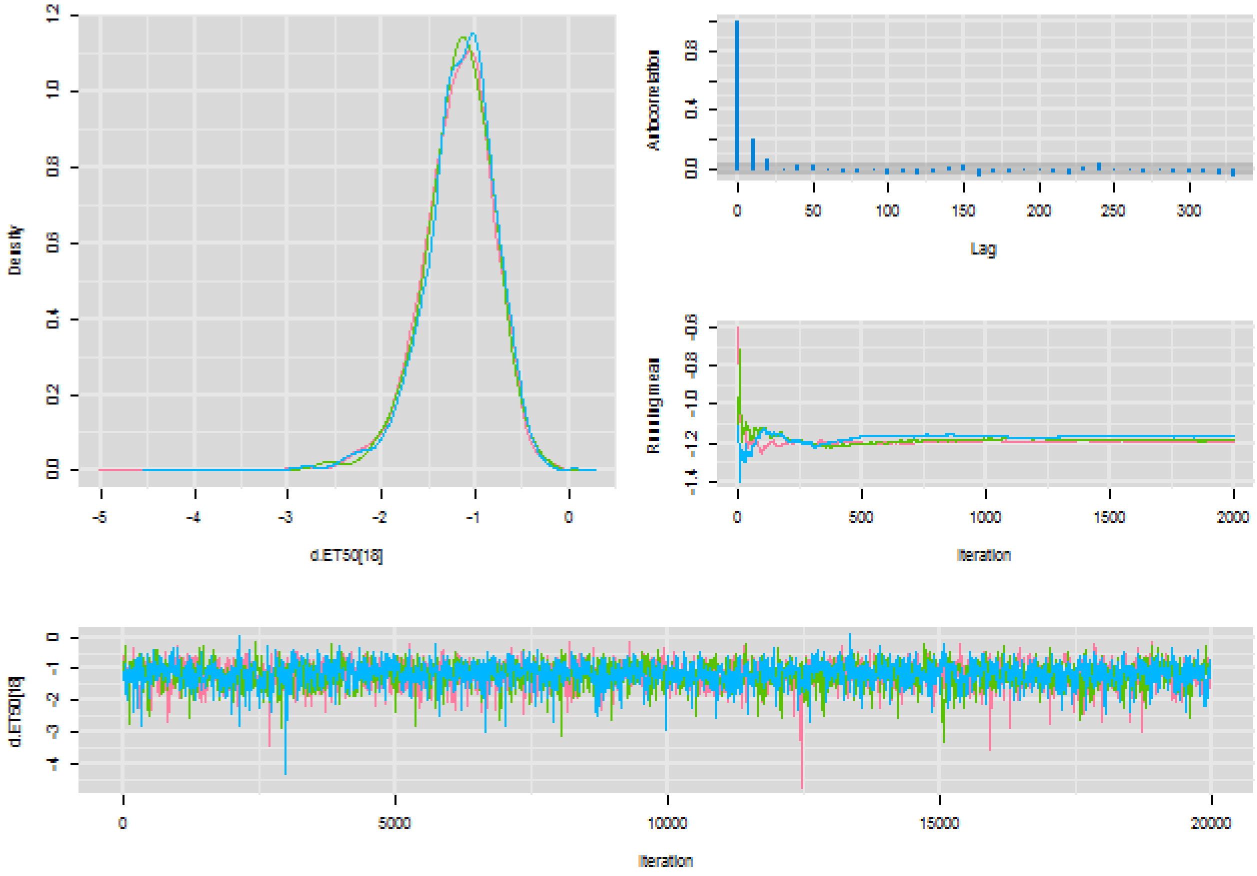

Diagnostics for d.ET50[19]

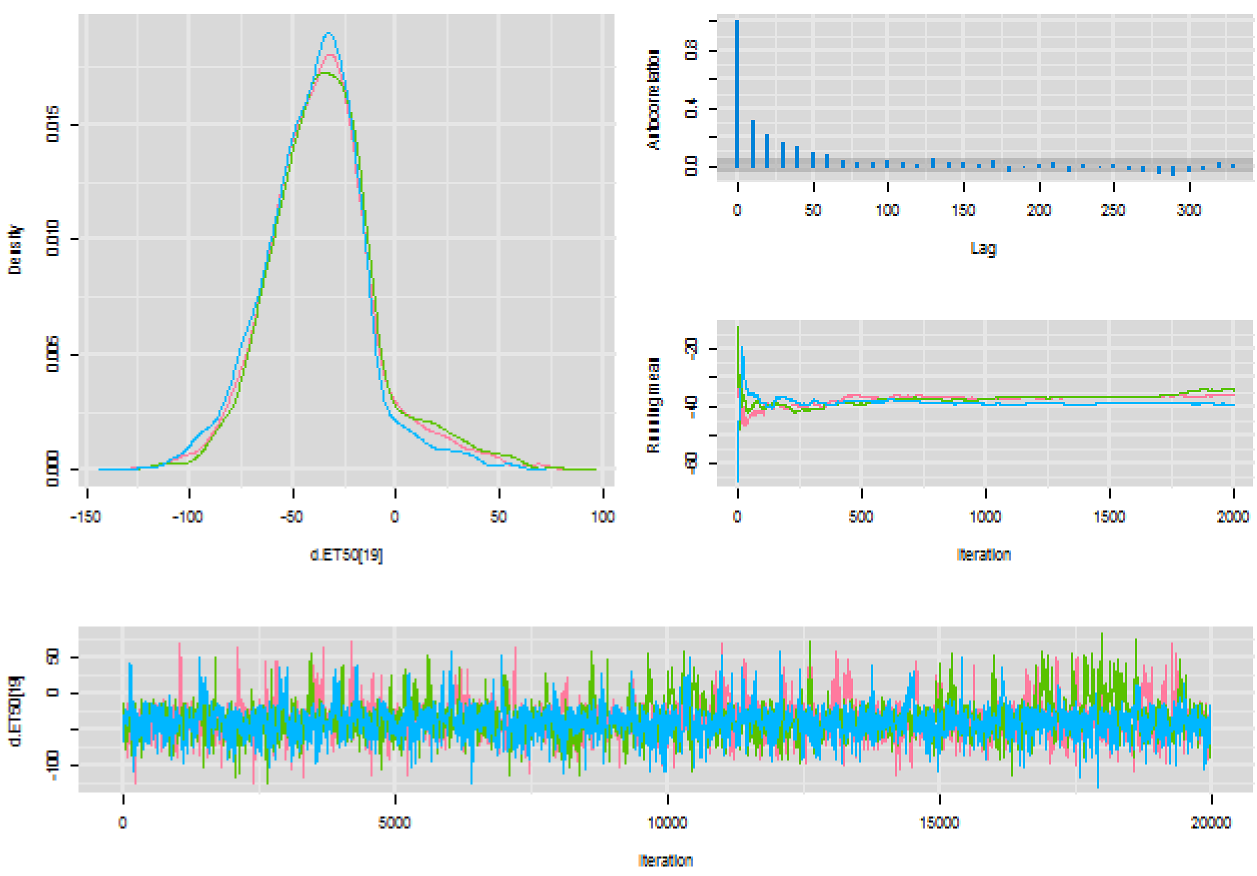

Diagnostics for d.ET50[20]

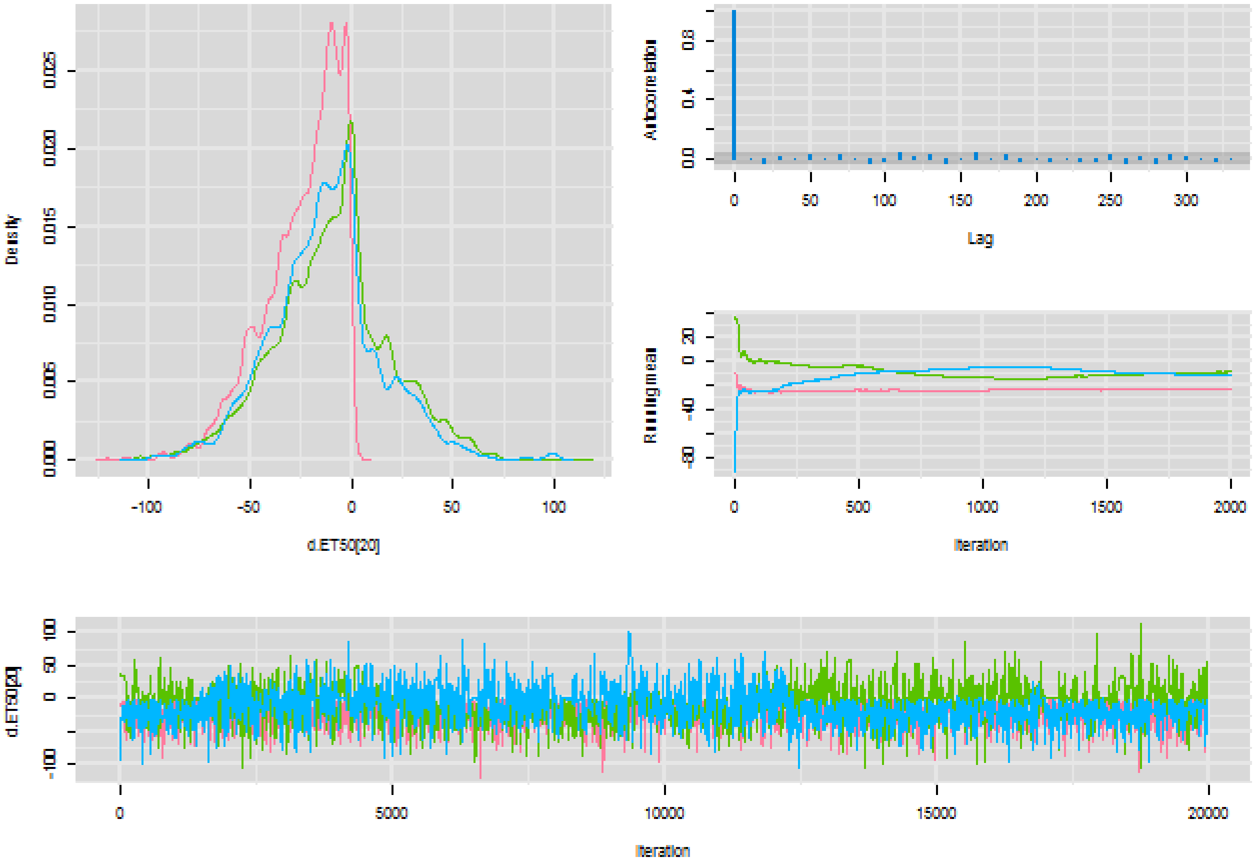

Diagnostics for d.ET50[21]

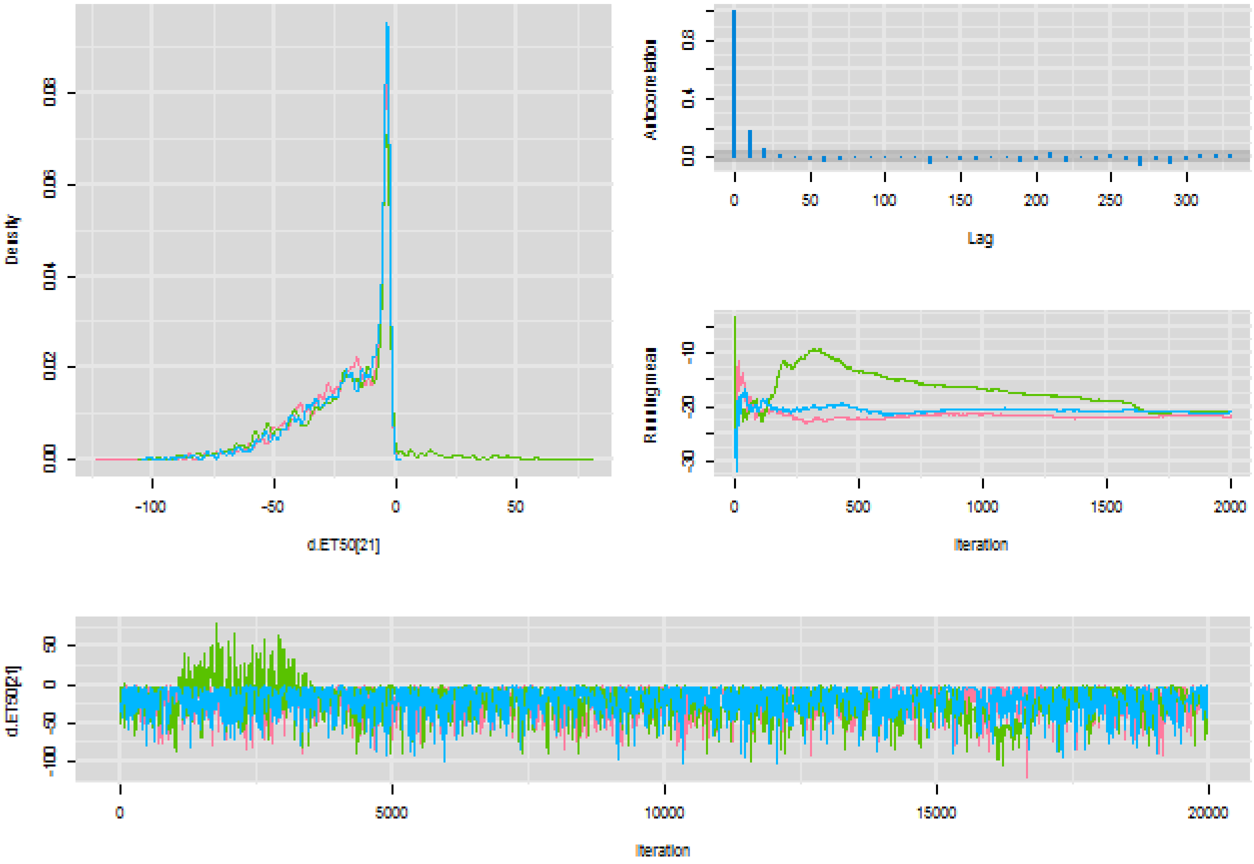

Diagnostics for d.ET50[22]

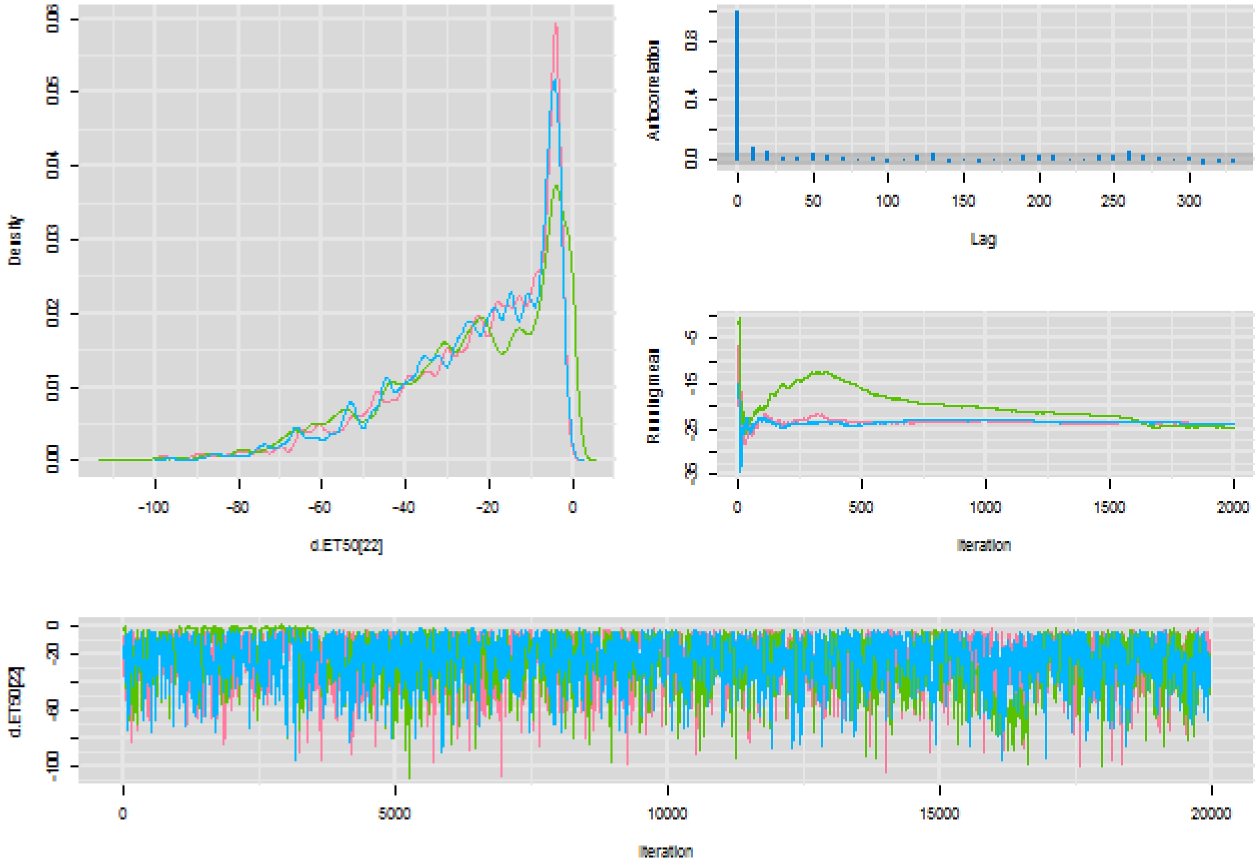

Diagnostics for d.ET50[23]

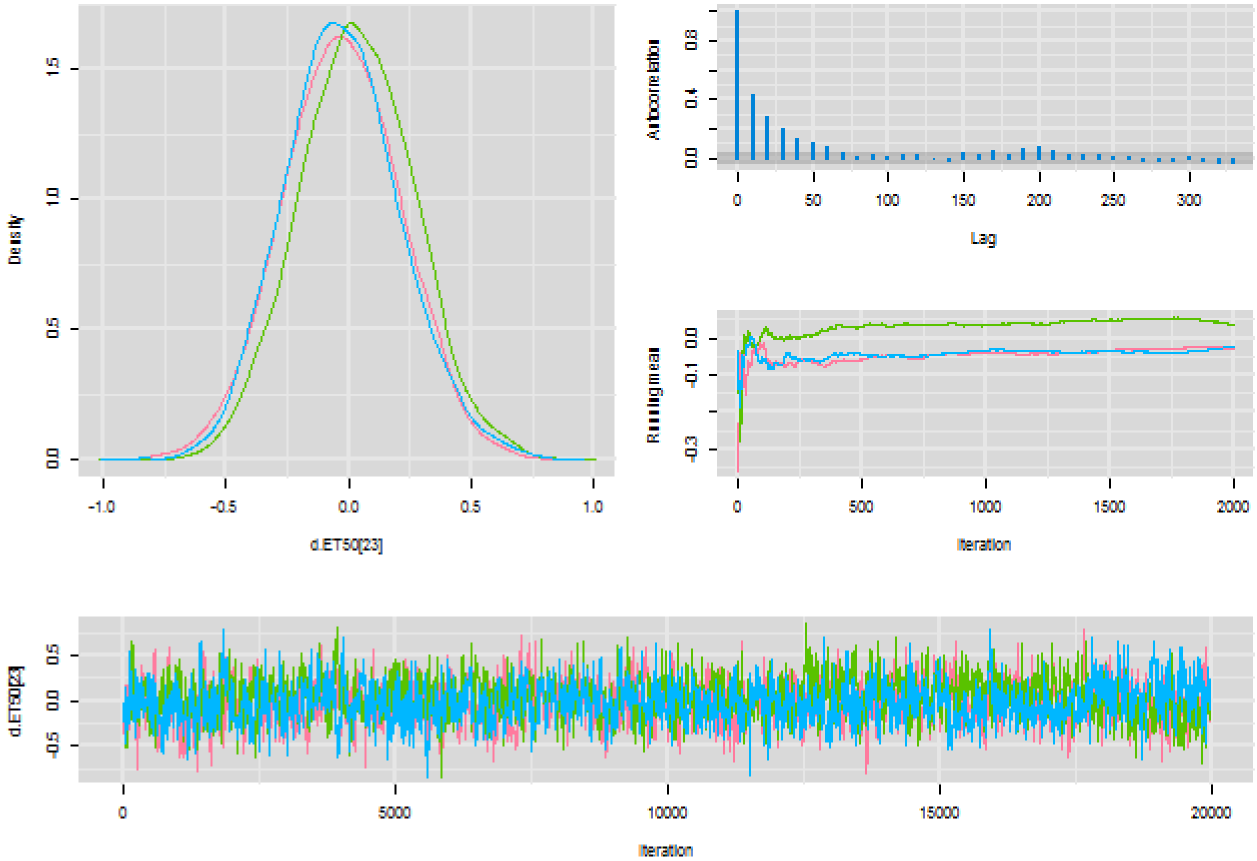

Diagnostics for d.ET50[24]

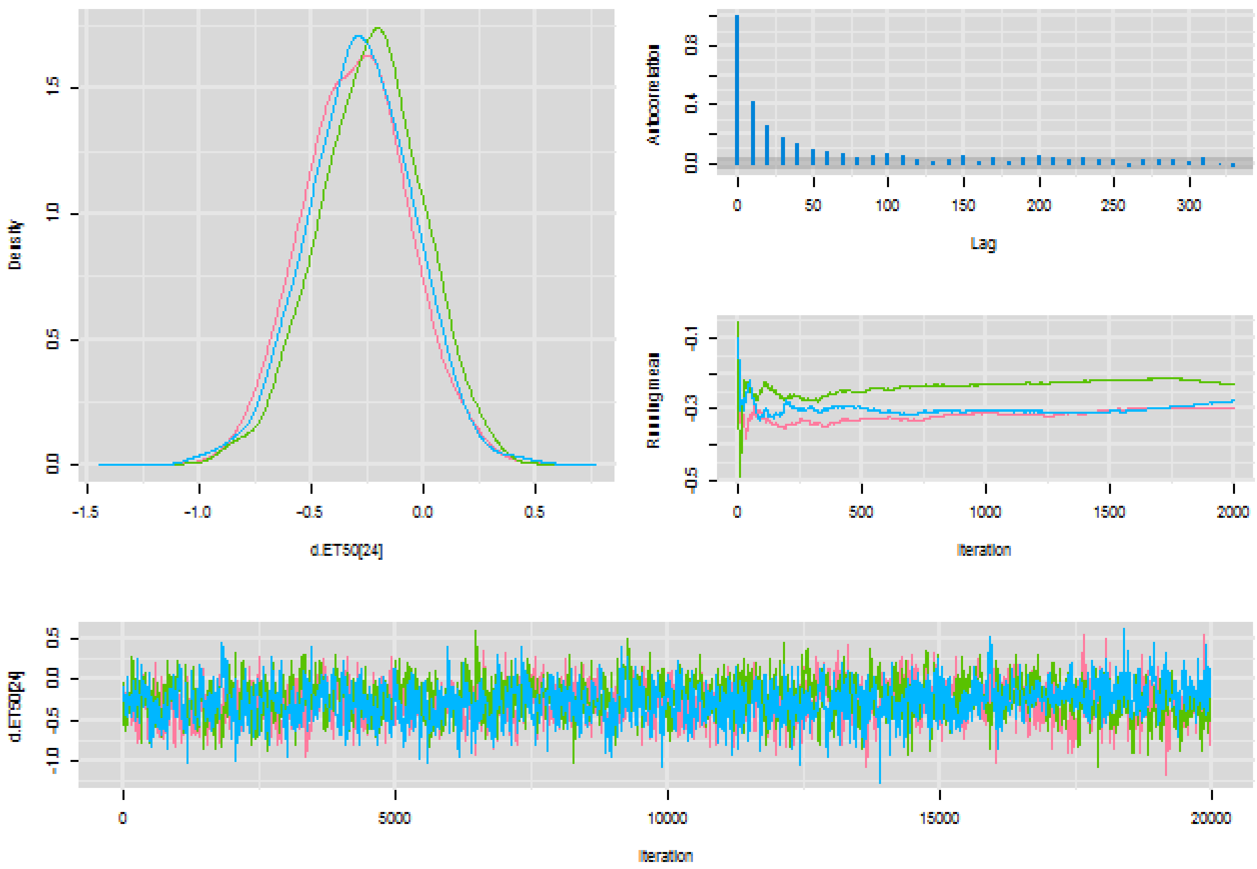

Diagnostics for d.ET50[25]

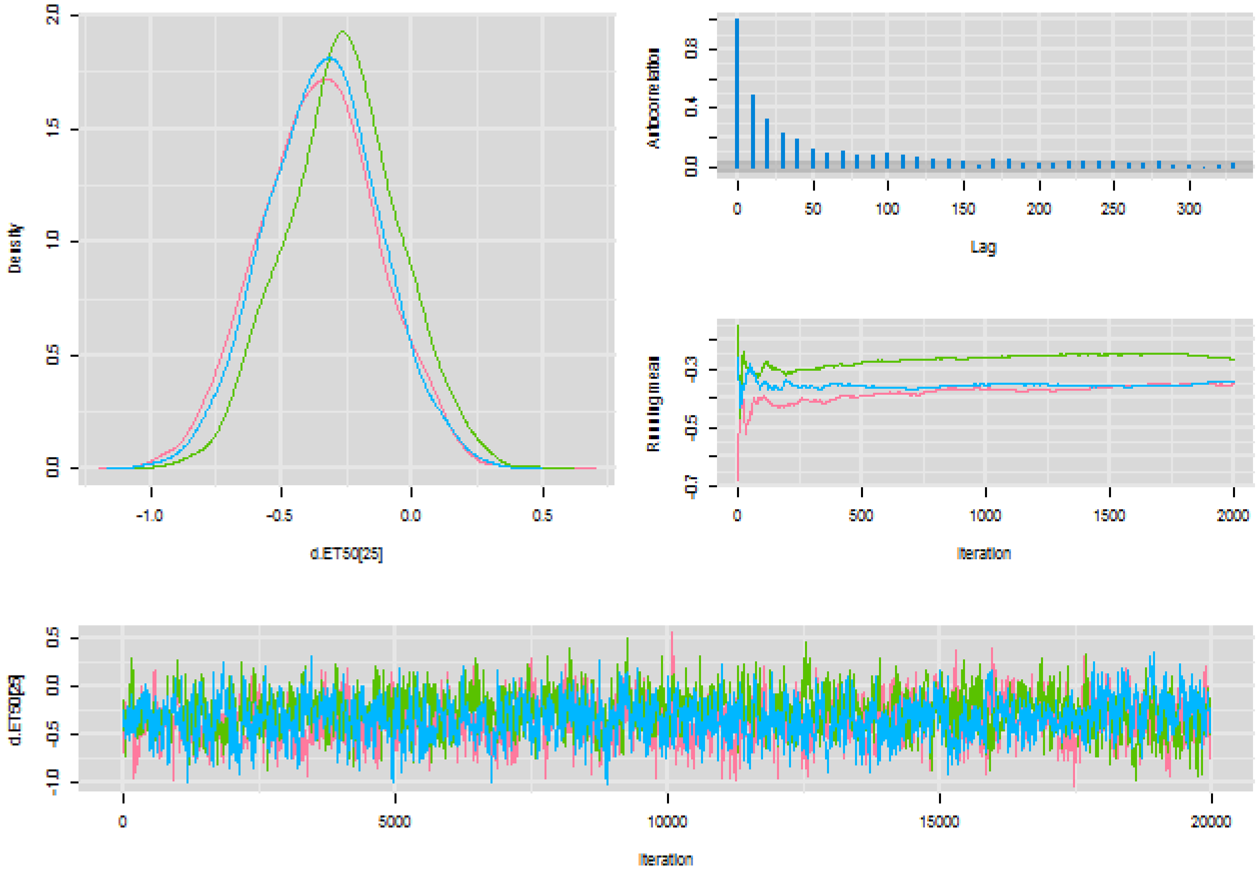

Diagnostics for d.ET50[26]

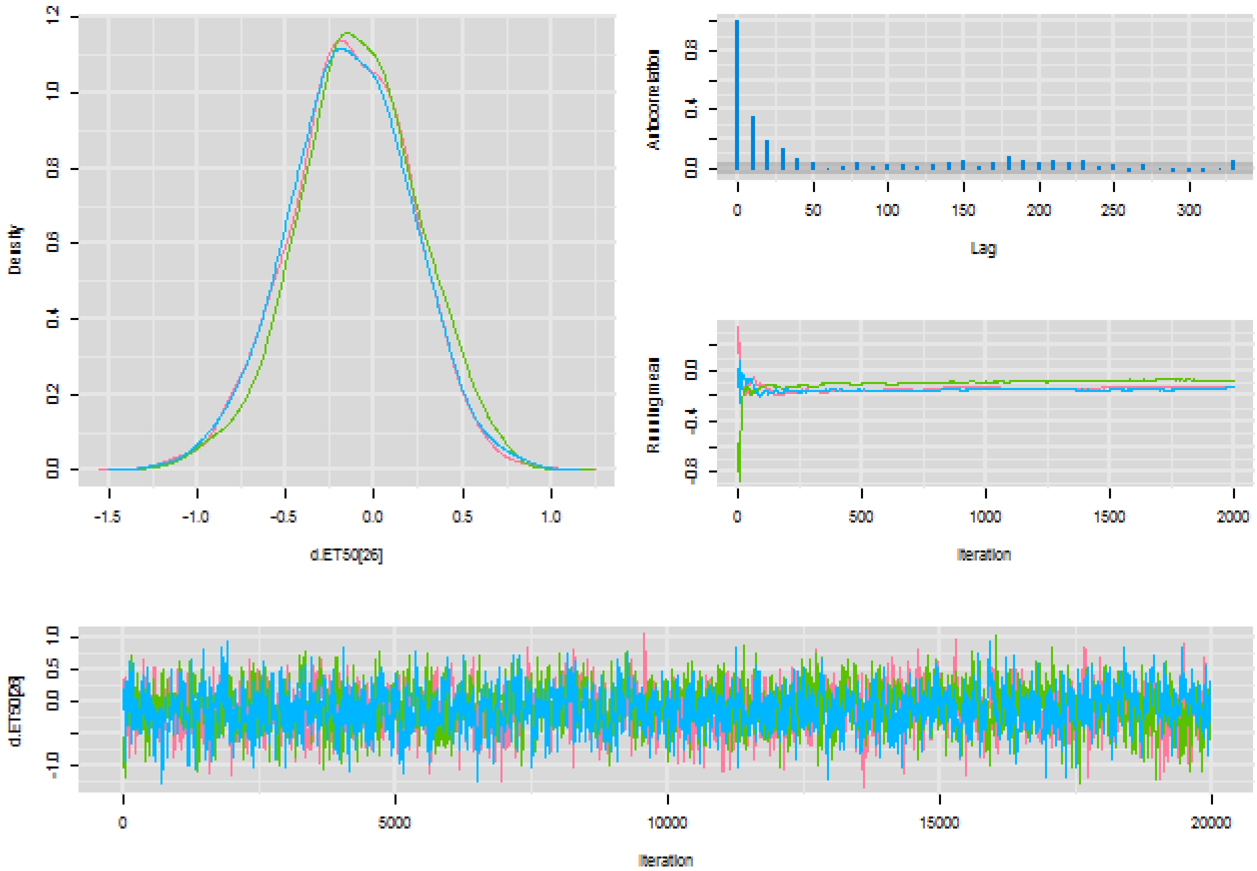

Diagnostics for d.ET50[27]

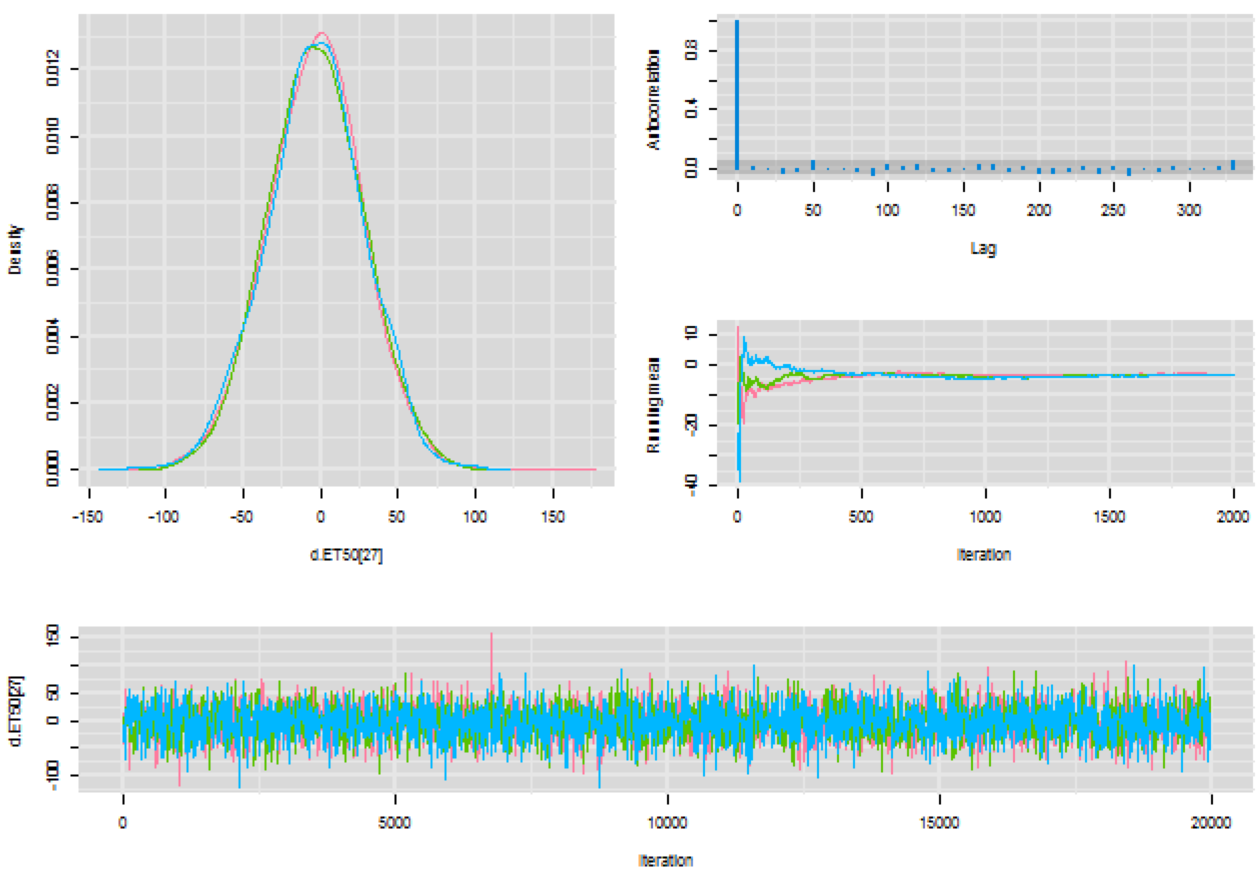

Diagnostics for d.ET50[28]

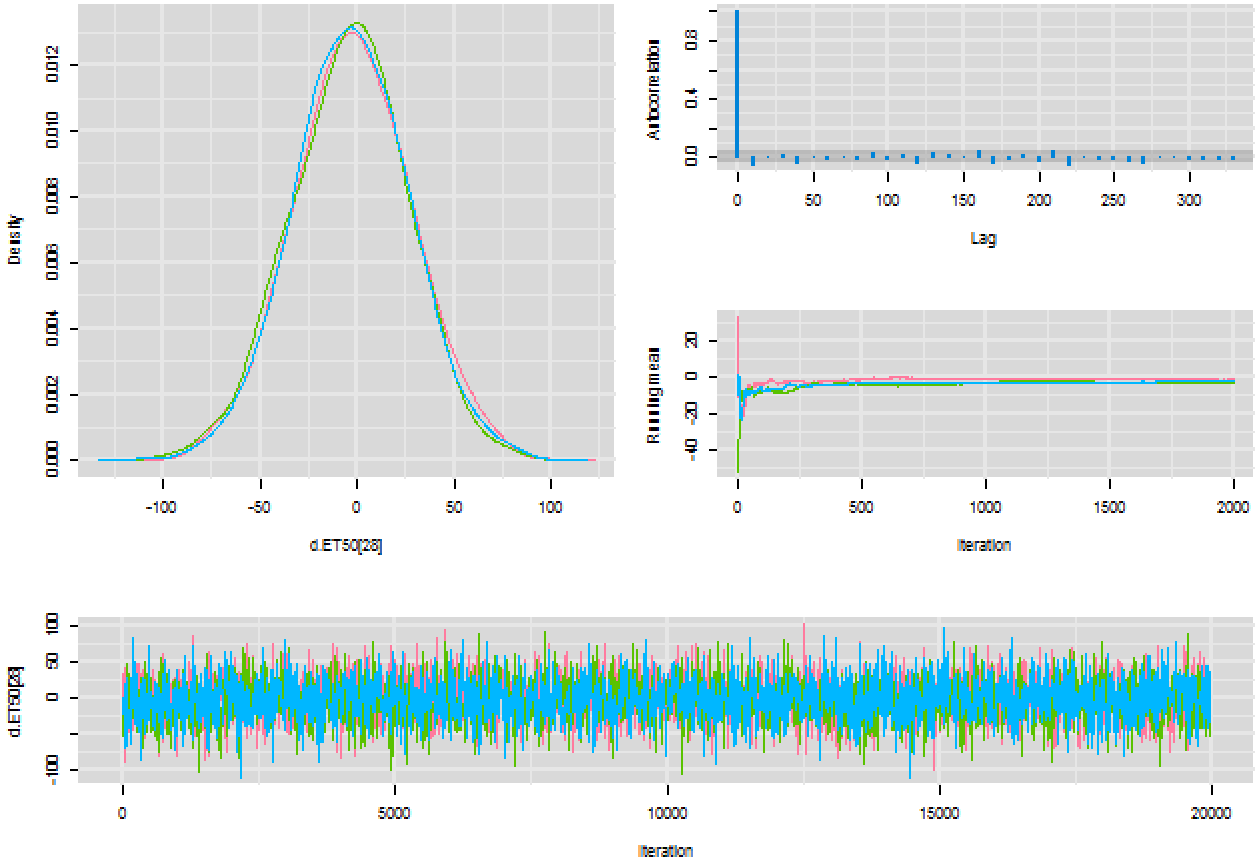

Diagnostics for d.ET50[29]

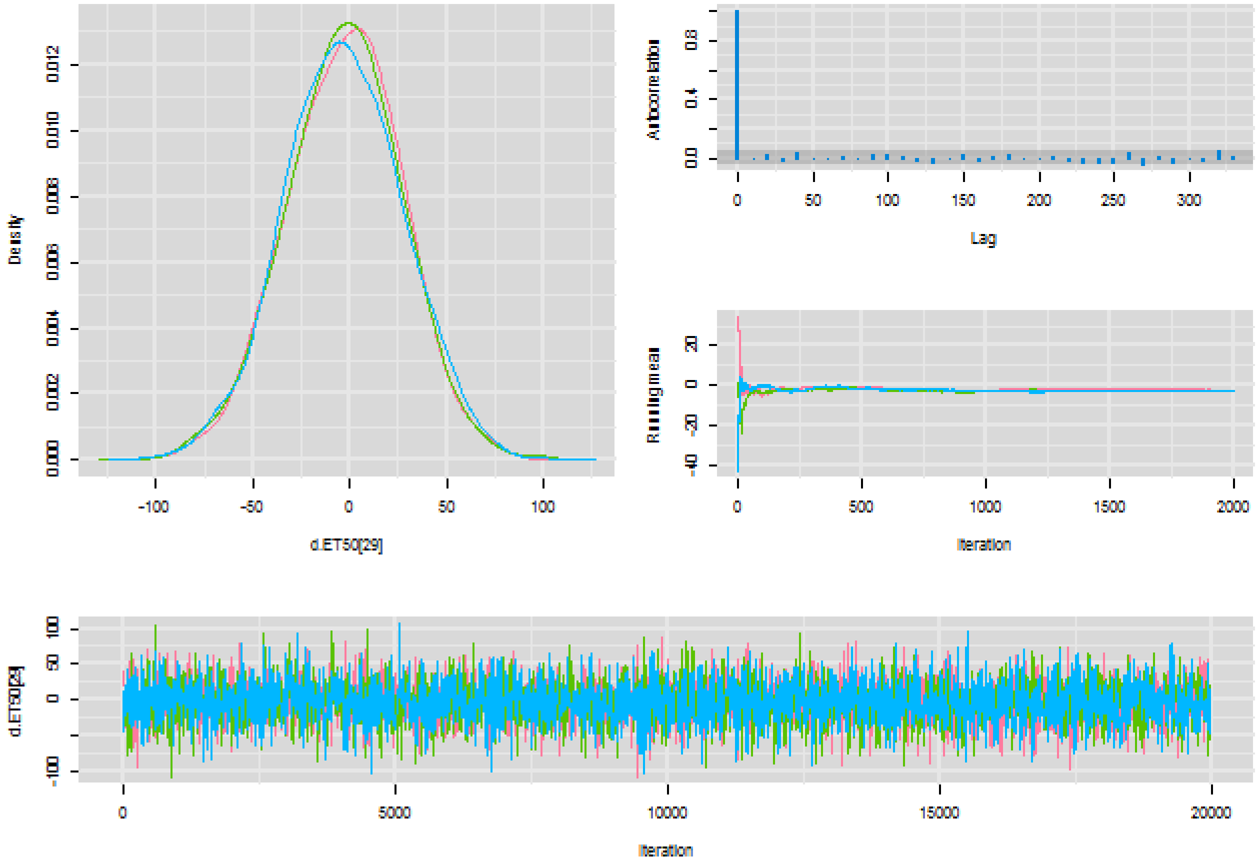

Plots for deviance

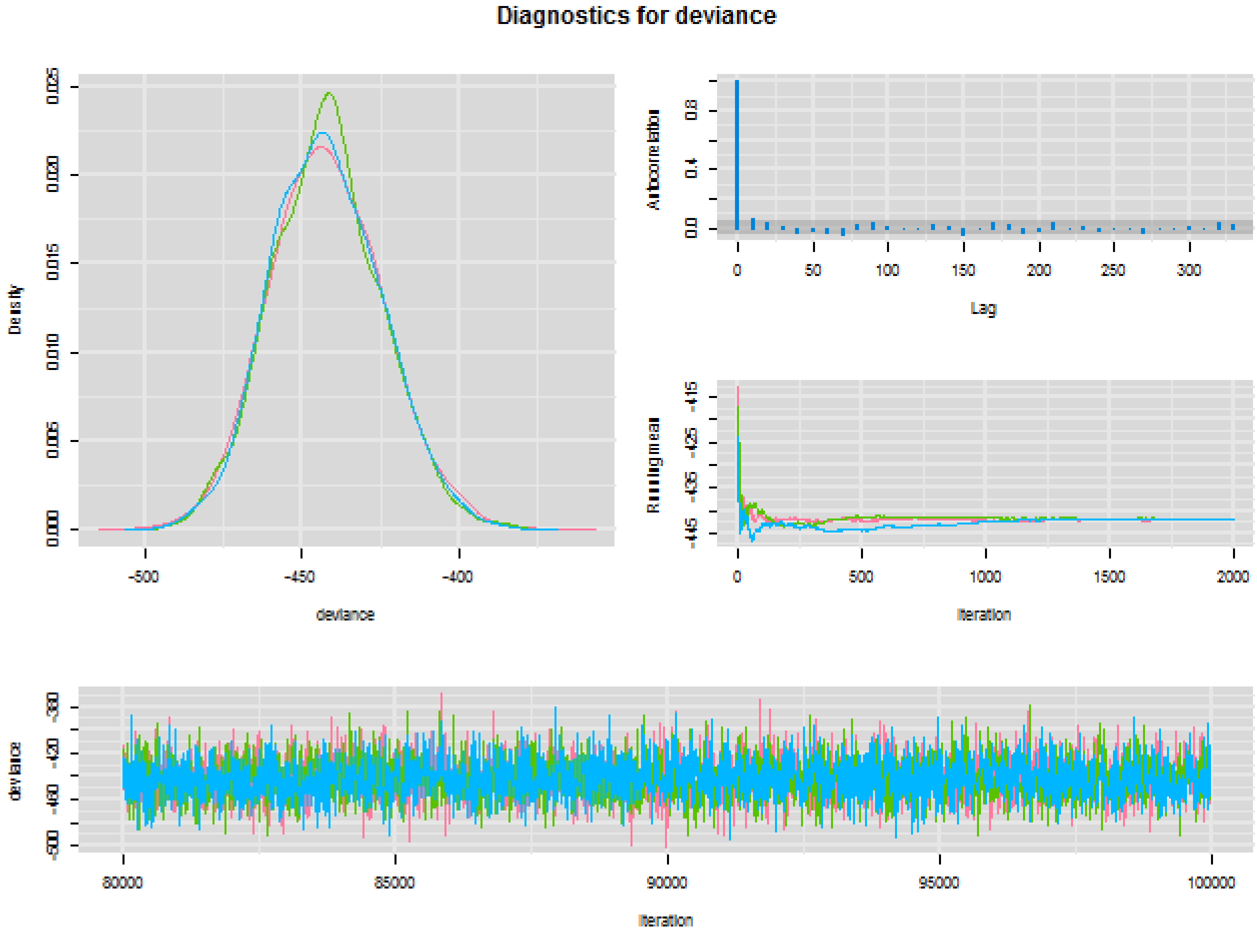

Supplement: Supplementary file 4 — Data S4: Supporting Information [file JRSM-10-267-s004.pdf]
