# Supplementary figures and images for: Modelling time‐course relationships with multiple treatments: Model‐based network meta‐analysis for continuous summary outcomes
Source: Res Synth Methods. 2019 May 29;10(2):267–86. doi: 10.1002/jrsm.1351 (PMC6563489; doi:10.1002/jrsm.1351)

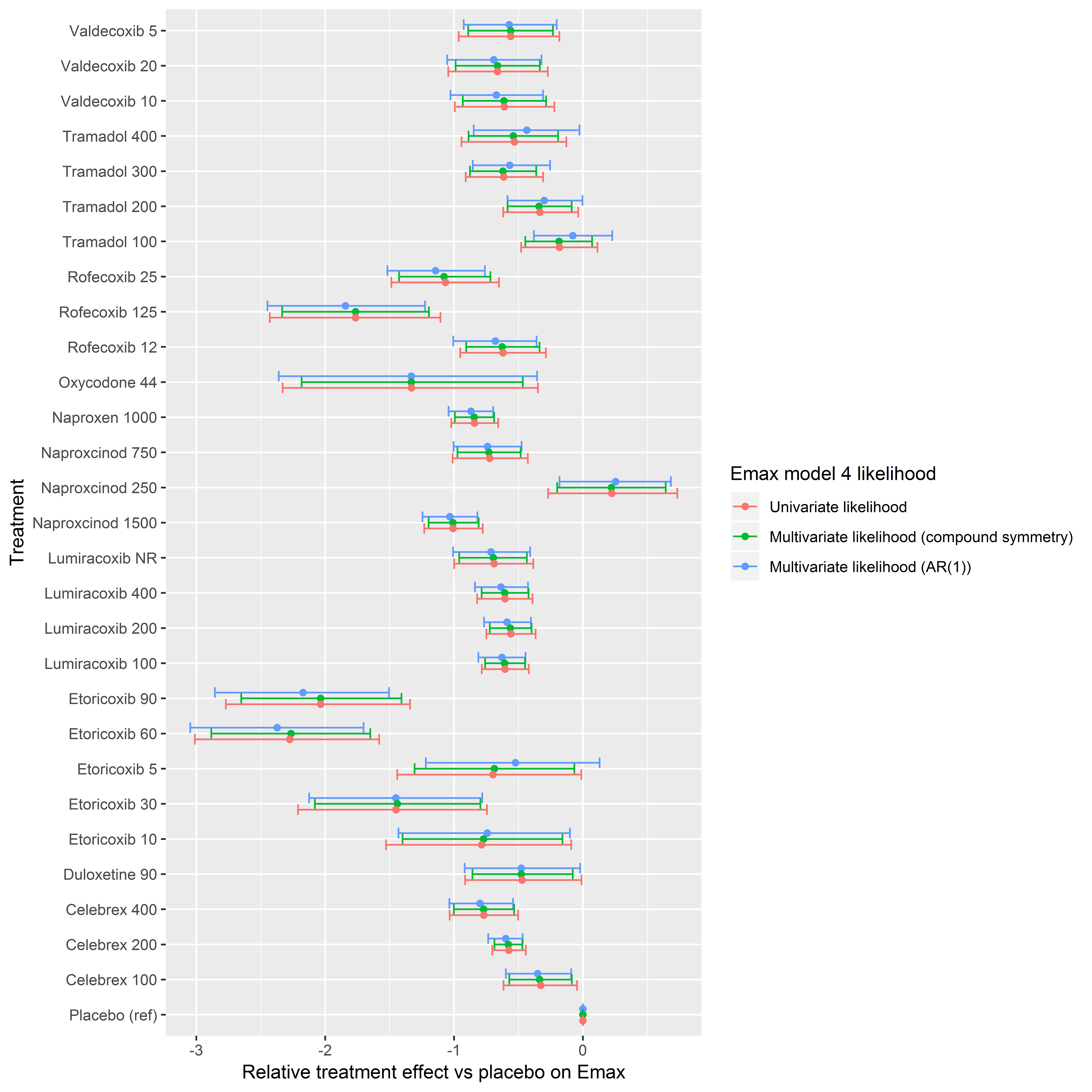

Supplement: Supplementary file 29 — Data S29: Supporting Information [file JRSM-10-267-s029.tif]

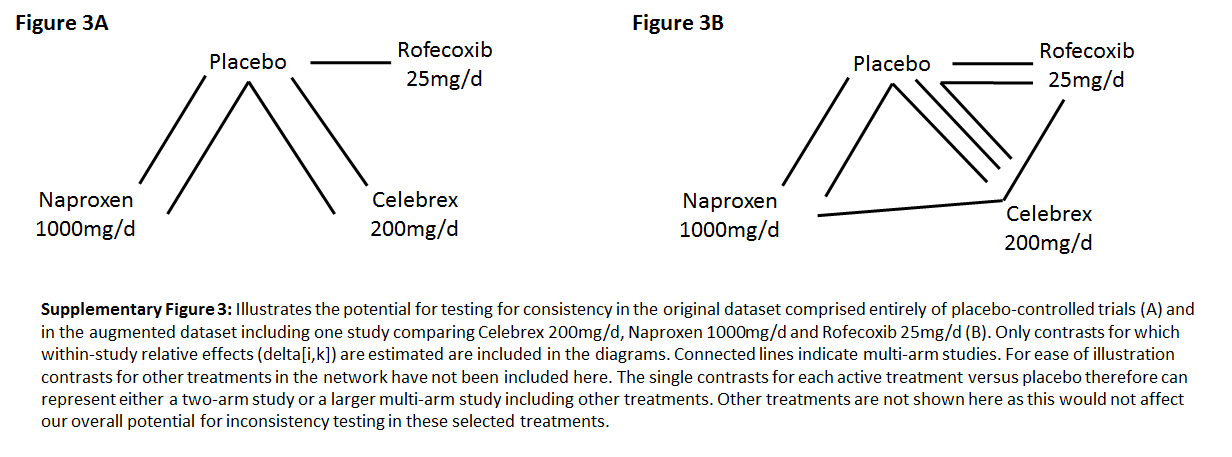

Supplement: Supplementary file 30 — Supplementary Figure 3: Illustrates the potential for testing for consistency in the original dataset comprised entirely of placebo‐controlled trials (A) and in the augmented dataset including one study comparing Celebrex 200 mg/d, Naproxen 1000 mg/d, and Rofecoxib 25 mg/d (B). Only contrasts for which within‐study relative effects (delta [i,k]) are estimated are included in the diagrams. Connected lines indicated multi‐arm studies. For ease of illustration, contrasts for other treatments in the network have not been included here. The single contrasts for each active treatment versus placebo therefore can represent either a two‐arm study or a larger multi‐arm study including other treatments. Other treatments are not shown here as this would not affect our overall potential for inconsistency testing in these selected treatments. [file JRSM-10-267-s030.tif]

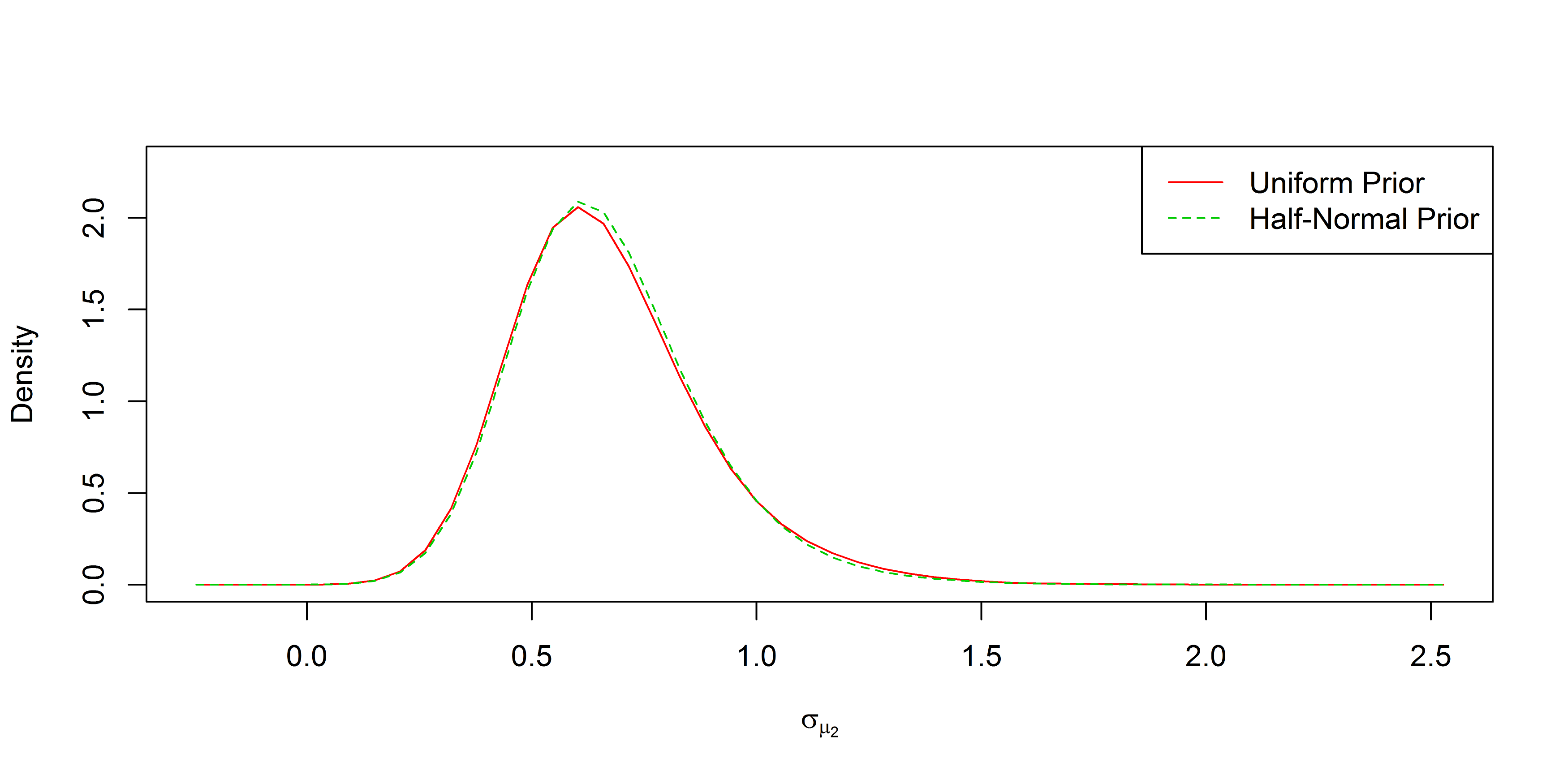

Supplement: Supplementary file 31 — Data S31: Supporting Information [file JRSM-10-267-s031.tif]

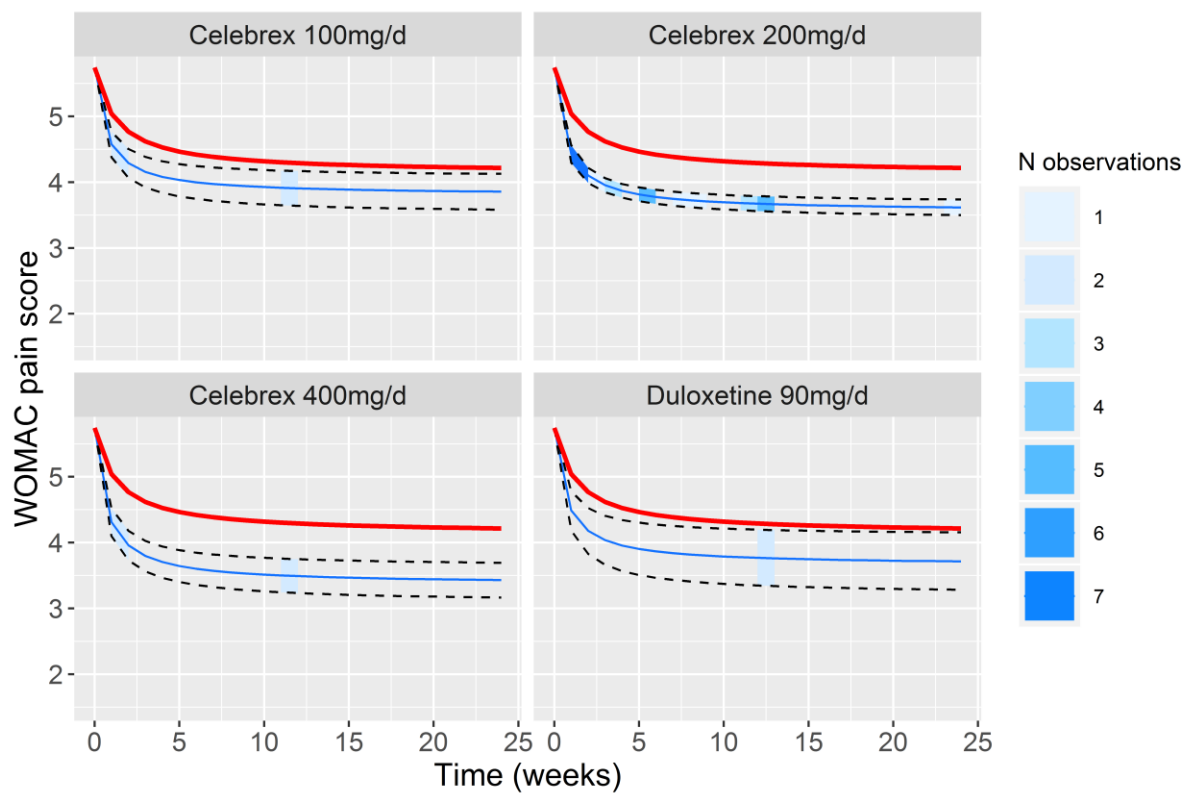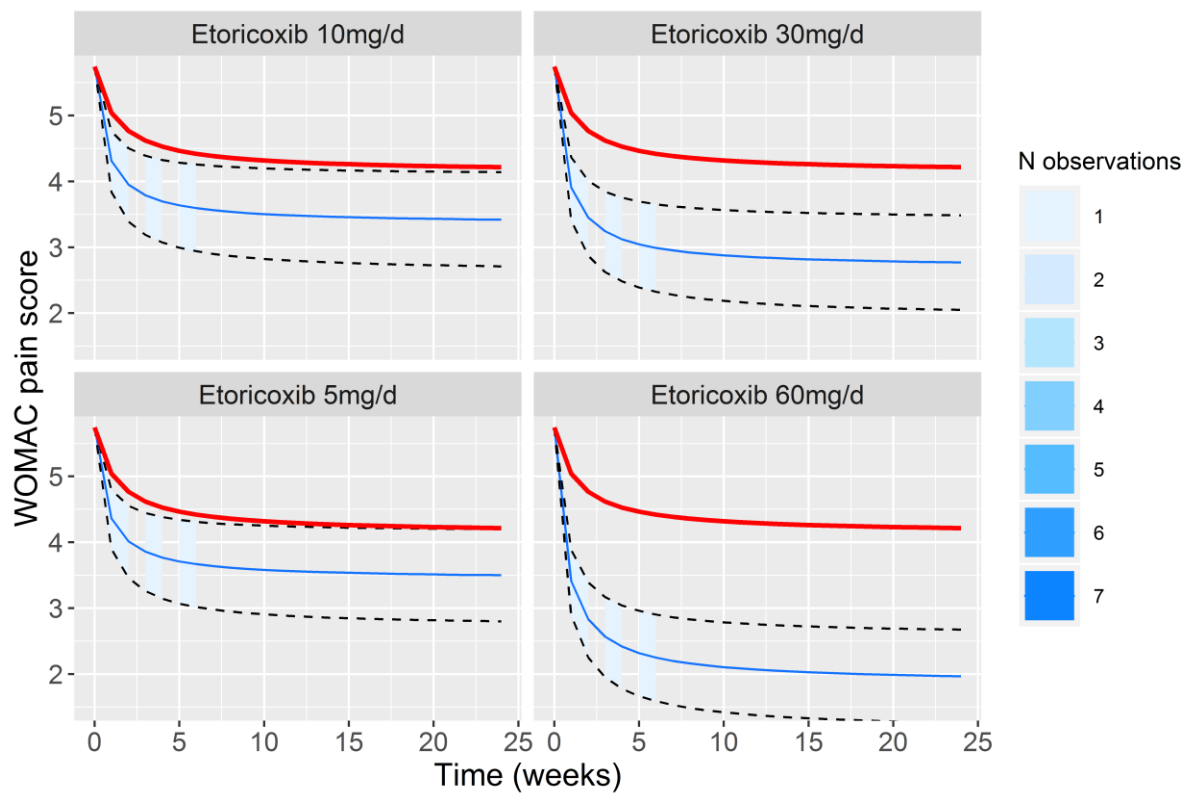

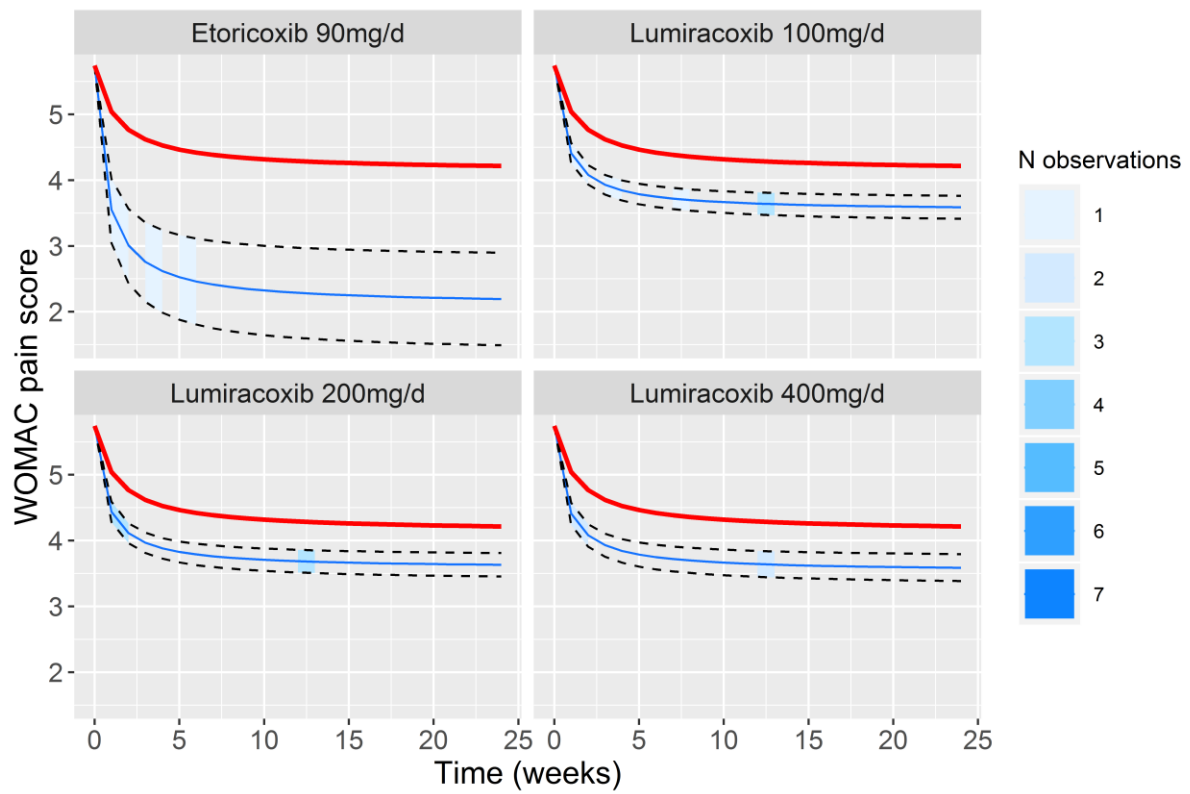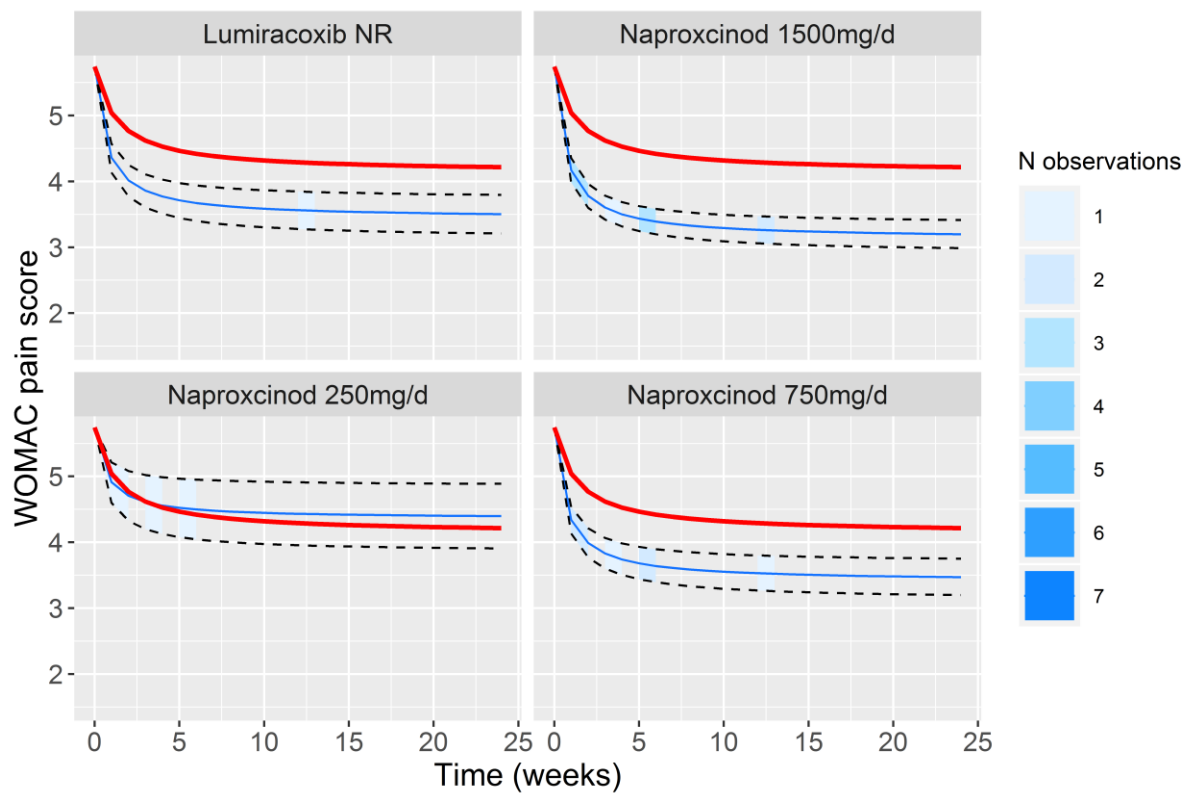

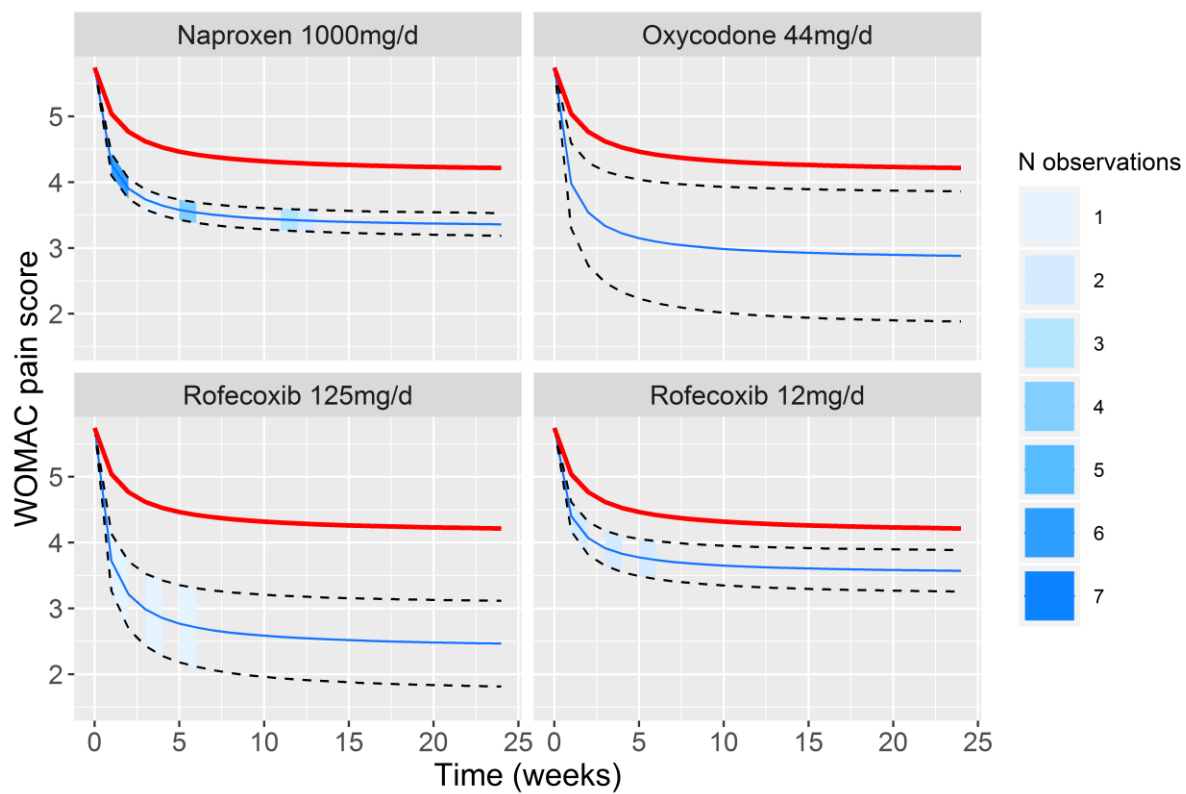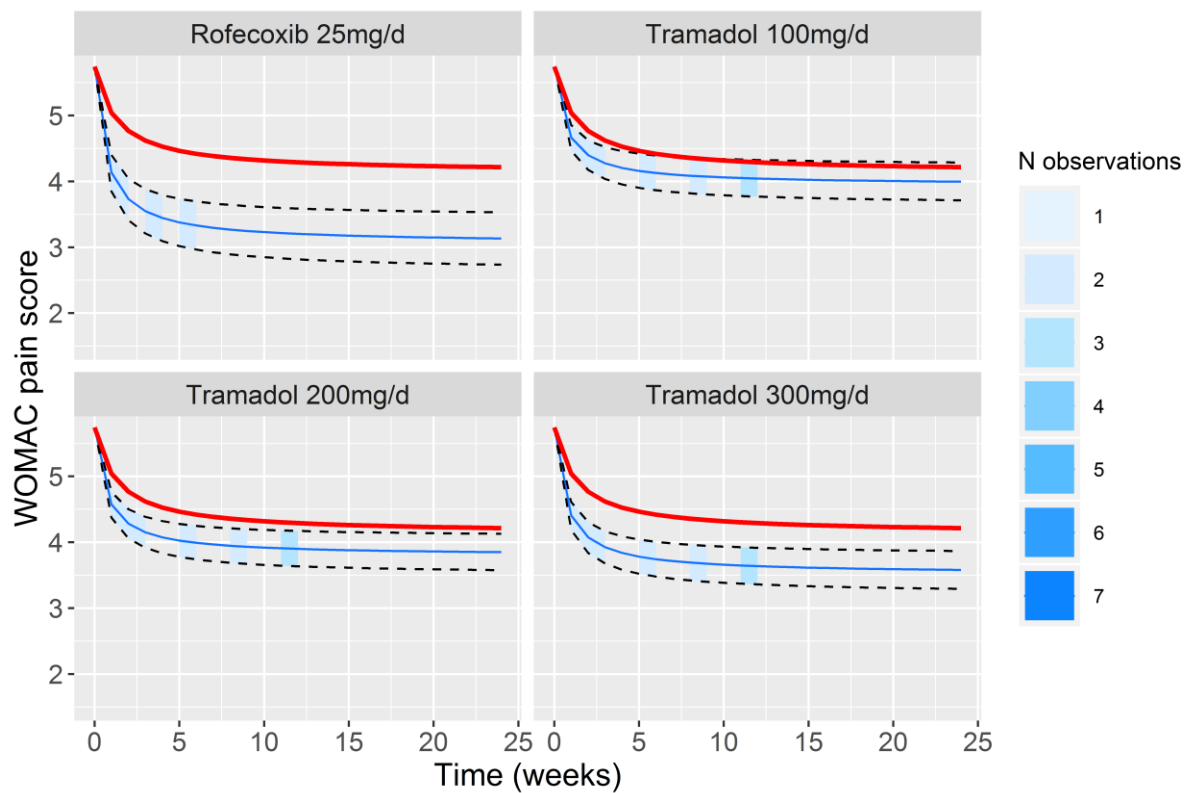

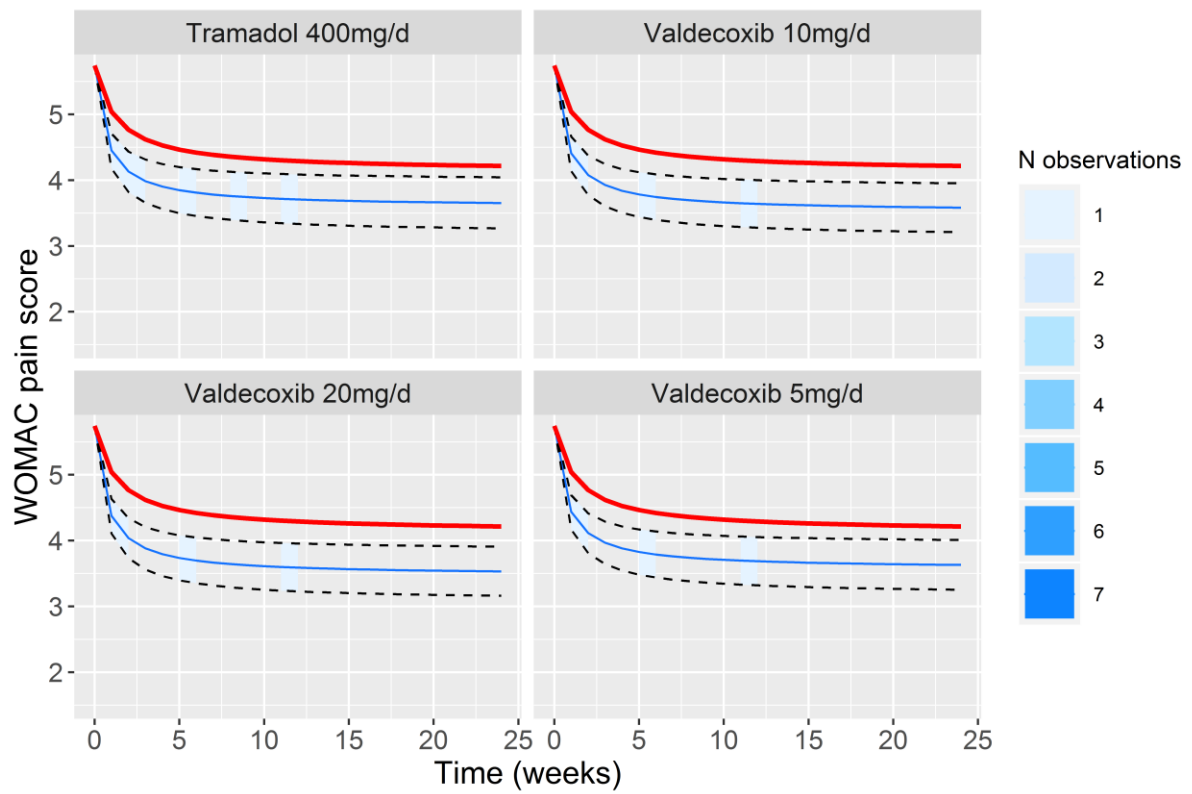

Supplement: Supplementary file 32 — Data S32: Supporting Information [file JRSM-10-267-s032.pdf]
